# New Variant of Multidrug-Resistant *Salmonella enterica* Serovar Typhimurium Associated with Invasive Disease in Immunocompromised Patients in Vietnam

**DOI:** 10.1128/mBio.01056-18

**Published:** 2018-09-04

**Authors:** Alison E. Mather, Tu Le Thi Phuong, Yunfeng Gao, Simon Clare, Subhankar Mukhopadhyay, David A. Goulding, Nhu Tran Do Hoang, Ha Thanh Tuyen, Nguyen Phu Huong Lan, Corinne N. Thompson, Nguyen Hoang Thu Trang, Juan Carrique-Mas, Ngo Tri Tue, James I. Campbell, Maia A. Rabaa, Duy Pham Thanh, Katherine Harcourt, Ngo Thi Hoa, Nguyen Vinh Trung, Constance Schultsz, Gabriel G. Perron, John E. Coia, Derek J. Brown, Chinyere Okoro, Julian Parkhill, Nicholas R. Thomson, Nguyen Van Vinh Chau, Guy E. Thwaites, Duncan J. Maskell, Gordon Dougan, Linda J. Kenney, Stephen Baker

**Affiliations:** a Department of Veterinary Medicine, University of Cambridge, Cambridge, United Kingdom; b Wellcome Trust Sanger Institute, Cambridge, United Kingdom; c The Hospital for Tropical Diseases, Wellcome Trust Major Overseas Programme, Oxford University Clinical Research Unit, Ho Chi Minh City, Vietnam; d Mechanobiology Institute, National University of Singapore, Singapore, Singapore; e The Hospital for Tropical Diseases, Ho Chi Minh City, Vietnam; f Centre for Tropical Medicine and Global Health, Nuffield Department of Clinical Medicine, Oxford University, Oxford, United Kingdom; g Department of Medical Microbiology, Academic Medical Center, University of Amsterdam, Amsterdam, The Netherlands; h Department of Biology, Bard College, Annandale-on-Hudson, New York, USA; i Scottish Microbiology Reference Laboratories, Glasgow Royal Infirmary, Glasgow, United Kingdom; j The Department of Medicine, University of Cambridge, Cambridge, United Kingdom; k The London School of Hygiene and Tropical Medicine, London, United Kingdom; l Jesse Brown Veterans Affairs Medical Center, University of Illinois—Chicago, Chicago, Illinois, USA; m Department of Microbiology and Immunology, University of Illinois—Chicago, Chicago, Illinois, USA; Pasteur Institute

**Keywords:** *Salmonella* Typhimurium, antimicrobial resistance, genomics, invasive salmonellosis

## Abstract

Nontyphoidal *Salmonella* (NTS), particularly *Salmonella enterica* serovar Typhimurium, is among the leading etiologic agents of bacterial enterocolitis globally and a well-characterized cause of invasive disease (iNTS) in sub-Saharan Africa. In contrast, *S*. Typhimurium is poorly defined in Southeast Asia, a known hot spot for zoonotic disease with a recently described burden of iNTS disease. Here, we aimed to add insight into the epidemiology and potential impact of zoonotic transfer and antimicrobial resistance (AMR) in *S*. Typhimurium associated with iNTS and enterocolitis in Vietnam. We performed whole-genome sequencing and phylogenetic reconstruction on 85 human (enterocolitis, carriage, and iNTS) and 113 animal *S*. Typhimurium isolates isolated in Vietnam. We found limited evidence for the zoonotic transmission of *S*. Typhimurium. However, we describe a chain of events where a pandemic monophasic variant of *S*. Typhimurium (serovar I:4,[5],12:i:− sequence type 34 [ST34]) has been introduced into Vietnam, reacquired a phase 2 flagellum, and acquired an IncHI2 multidrug-resistant plasmid. Notably, these novel biphasic ST34 *S*. Typhimurium variants were significantly associated with iNTS in Vietnamese HIV-infected patients. Our study represents the first characterization of novel iNTS organisms isolated outside sub-Saharan Africa and outlines a new pathway for the emergence of alternative *Salmonella* variants into susceptible human populations.

## Abstract (precis)

*Salmonella* Typhimurium is a major diarrheal pathogen and associated with invasive nontyphoid *Salmonella* (iNTS) disease in vulnerable populations. We present the first characterization of iNTS organisms in Southeast Asia and describe a different evolutionary trajectory from that of organisms causing iNTS in sub-Saharan Africa. In Vietnam, the globally distributed monophasic variant of *Salmonella* Typhimurium, the serovar I:4,[5],12:i:− ST34 clone, has reacquired a phase 2 flagellum and gained a multidrug-resistant plasmid to become associated with iNTS disease in HIV-infected patients. We document distinct communities of *S*. Typhimurium and I:4,[5],12:i:− in animals and humans in Vietnam, despite the greater mixing of these host populations here. These data highlight the importance of whole-genome sequencing surveillance in a One Health context in understanding the evolution and spread of resistant bacterial infections.

## INTRODUCTION

Nontyphoidal *Salmonella* (NTS) is a common cause of bacterial enterocolitis (diarrheal disease) in humans and animals (1). Additionally, subsets of NTS organisms are also associated with an aggressive invasive disease in susceptible humans (2) and have been shown to cause invasive disease in animals (3, 4). Invasive NTS (iNTS) infections principally occur in sub-Saharan Africa, are life-threatening, and are commonly associated with malnourished infants and the immunocompromised, particularly those infected with HIV. Notably, iNTS disease is generally uncommon outside sub-Saharan Africa, but the disease has recently been described in a comparatively small patient cohort in Southeast Asia (5). The microbiological reservoirs of these two NTS disease presentations are distinct, with organisms causing enterocolitis in humans in industrialized countries primarily thought to arise through the food chain (1). In contrast, the principal source of the organisms causing iNTS in sub-Saharan Africa is thought to be the human population (6).

One of the most common NTS serovars associated with both enterocolitis and iNTS in humans is Salmonella enterica subsp. enterica serovar Typhimurium (*S*. Typhimurium). This serovar is globally ubiquitous and can be isolated from a range of other animal species. Successive human epidemics of *S*. Typhimurium have been described over the past several decades, many of which have been caused by variants that exhibit resistance to multiple antimicrobials, including those recommended for clinical care. These epidemic variants are of great concern, as antimicrobial-resistant *Salmonella* infections are associated with a higher probability of hospitalization and treatment failure, leading to a prolonged infection and increased likelihood of onward transmission (7).

An accurate understanding of how antimicrobial-resistant *Salmonella* variants emerge and spread is essential for controlling their geographic scope and limiting their potential public health impact. The advent of high-throughput whole-genome sequencing (WGS) and phylogenetics has enabled detailed investigations of the sources and potential transmission routes of *S*. Typhimurium variants (2, 8). These studies permitted the identification of distinct *S*. Typhimurium populations found in colocated animals and humans in an industrialized country (8) and outlined the complex phylogeography of an epidemic iNTS-causing *S*. Typhimurium across sub-Saharan Africa (2).

While there is a good understanding of the *S*. Typhimurium genomic landscape in Europe and sub-Saharan Africa, such an investigation has not yet been performed extensively for *S*. Typhimurium originating from Southeast Asia, a known global hot spot for zoonotic disease. Vietnam is a low-middle-income country (LMIC) in Southeast Asia, characterized by widespread human-animal interaction and the excessive use of antimicrobials in humans and agriculture (9, 10). Here, we exploited WGS, phylogenetic reconstruction, and genomic analysis to provide insight into the epidemiology and potential impact of zoonotic transfer and antimicrobial resistance (AMR) in *S*. Typhimurium and its monophasic variant *Salmonella* I:4,[5],12:i:− in Vietnam. Additionally, we aimed to define the genetic characteristics of the recently isolated iNTS *S*. Typhimurium/*S.* I:4,[5],12:i:− in Vietnam, representing the first such investigation of a novel collection of iNTS organisms isolated outside sub-Saharan Africa.

## RESULTS

**FIG 1:**
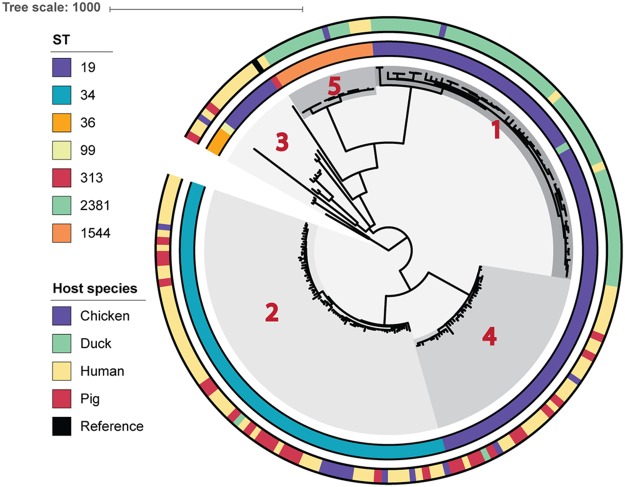
Maximum-likelihood phylogeny of 198 *Salmonella* Typhimurium/*S*. I:4,[5],12:i:− isolates from Vietnam. Reads were mapped to reference *S*. Typhimurium SL1344, with host species, multilocus sequence type (ST), and BAPS cluster (in red) marked. The scale bar represents the number of nonrecombinogenic single nucleotide polymorphisms per branch.

The genomes of 85 human *S*. Typhimurium and *S*. I:4,[5],12:i:− isolates collected between 2008 and 2013 (36 associated with enterocolitis, 41 associated with iNTS, and 8 from asymptomatic carriage) and 113 animal *S*. Typhimurium and *S*. I:4,[5],12:i:− isolates collected between 2011 and 2013 (chickens [*n* = 14], ducks [*n* = 70], and pigs [*n* = 29]) (see Table S1 in the supplemental material) in southern Vietnam were sequenced using an Illumina HiSeq2000 sequencer. By mapping the genome sequence reads against the *S*. Typhimurium SL1344 reference sequence, we were able to reconstruct the phylogenetic relationship between these contemporary Vietnamese isolates from different sources (Fig. 1). Using hierBAPS (11), the isolates clustered into five distinct clades (Table S3). The most striking observation within this initial phylogeny was an apparent lack of mixing between animal and human isolates (*P* = 0.0005). Notably, organisms in three of the five clades were predominantly associated with a single host species (clade 3, 80% [12/15] of the isolates from humans, *D* = 0.78, *P* > 0.10; clades 1 and 5, 95% [56/59] and 75% [12/16] of isolates from ducks, *D* = 0.73/*P* > 0.10 and *D* = 0.44/*P* > 0.10, respectively). Clades 2 and 4 contained a more comparable number of human and animal *S*. Typhimurium/*S*. I:4,[5],12:i:− isolates than clades 1, 3, and 5 (Table S3). There was a significant phylogenetic association with the host species of origin (animal or human) in clades 2 and 4, indicating nonrandom clustering of isolates by host species (clade 2, *D* = 0.18, *P* = 0; clade 4, *D* = −0.06, *P* = 0.002).

10.1128/mBio.01056-18.4 TABLE S1  Details and epidemiological data for the 198 *Salmonella* Typhimurium/*Salmonella* I:4,[5],12:i:− isolates from Vietnam. Download TABLE S1, PDF file, 0.1 MB. Copyright © 2018 Mather et al. 2018 Mather et al. This content is distributed under the terms of the Creative Commons Attribution 4.0 International license.

**FIG 2:**
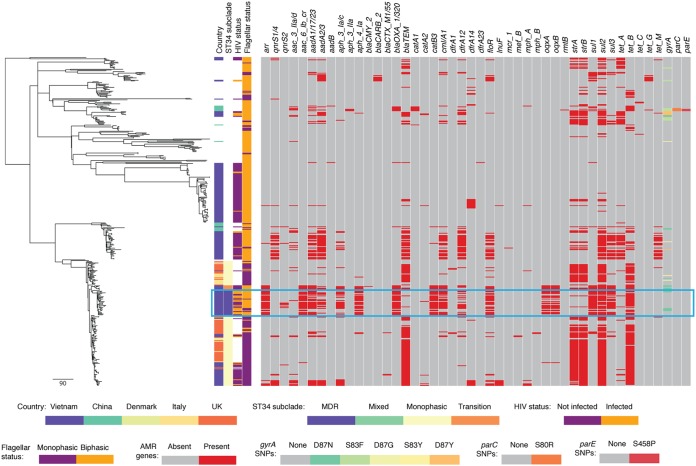
Maximum-likelihood phylogeny of 418 *Salmonella* Typhimurium/*S*. I:4,[5],12:i:− isolates from Vietnam and other countries. Reads were mapped to reference monophasic *S*. Typhimurium variant *S*. I:4,[5],12:i:− SO4698-09, with country of origin, ST34 subgroup, HIV status, flagellar status, and presence or absence of antimicrobial resistance determinants mapped against the phylogeny. The blue box indicates the multidrug-resistant (MDR) ST34 subgroup. The scale bar represents the number of nonrecombinogenic single nucleotide polymorphisms per branch.

Clade 2 was comprised of isolates belonging solely to multilocus sequence type 34 (ST34). This ST encompasses the European clone associated with the present *S*. Typhimurium variant pandemic, which is the monophasic *S*. I:4,[5],12:i:− (12). We expanded our data set with WGS of monophasic and biphasic *S*. Typhimurium and *S*. I:4,[5],12:i:− accessed from public databases, which included ST34 isolates from other countries (2, 12–14) (Table S2; Fig. S1) and two new sequences from organisms isolated in Scotland. By doing so, we could demonstrate that the ancestral subclade of the Vietnamese ST34 isolates was the European ST34 *S*. I:4,[5],12:i:− clone (Fig. 2) (12).

10.1128/mBio.01056-18.2 FIG S1  Maximum likelihood phylogeny of *S*. Typhimurium/*S*. I:4,[5],12:i:− isolates from animals and humans mapped to the monophasic reference SO4698-09, including 198 from Vietnam and 220 from other countries with multilocus sequence type (ST) and the available metadata on country of origin. The scale bar represents the number of nonrecombinogenic single nucleotide polymorphisms per branch. Download FIG S1, PDF file, 0.2 MB. Copyright © 2018 Mather et al. 2018 Mather et al. This content is distributed under the terms of the Creative Commons Attribution 4.0 International license.

10.1128/mBio.01056-18.5 TABLE S2  Details and epidemiological data for isolates in the context collection. Download TABLE S2, PDF file, 0.1 MB. Copyright © 2018 Mather et al. 2018 Mather et al. This content is distributed under the terms of the Creative Commons Attribution 4.0 International license.

10.1128/mBio.01056-18.6 TABLE S3  Numbers of isolates from humans and animals (combined animal species) in Vietnam per BAPS clade. Download TABLE S3, PDF file, 0.03 MB. Copyright © 2018 Mather et al. 2018 Mather et al. This content is distributed under the terms of the Creative Commons Attribution 4.0 International license.

Further interrogation of the phylogenetic structure revealed that the Vietnamese ST34 isolates could be further subdivided into three clear subgroups, which we named ancestral, transitional, and multidrug resistant (MDR), all of which had high bootstrap support values within the phylogenetic tree (Fig. S2). We observed that the majority of organisms within the ancestral ST34 subgroup, comprised of mostly European isolates, were genetically monophasic (Fig. 2). In contrast, there was a cluster of 31 Vietnamese isolates that were predominantly biphasic and further characterized by an extensive complement of AMR genes (MDR subgroup) (Tables S4 and S5). This complement of AMR genes was distinct from the classical AMR gene profile associated with resistance to ampicillin, streptomycin, sulfonamide, and tetracycline (ASSuT), which is typically observed in monophasic ST34 isolates.

10.1128/mBio.01056-18.3 FIG S2  Subset of the maximum likelihood phylogeny from Fig. 2, showing the ST34 *S*. Typhimurium/*S*. I:4,[5],12:i:− isolates from the Vietnam and context collections, mapped to monophasic reference SO4698-09. Key bootstrap values relating to the ST34 subclades are indicated in red on the relevant nodes. The scale bar represents the number of nonrecombinogenic single nucleotide polymorphisms per branch. Download FIG S2, PDF file, 0.2 MB. Copyright © 2018 Mather et al. 2018 Mather et al. This content is distributed under the terms of the Creative Commons Attribution 4.0 International license.

10.1128/mBio.01056-18.7 TABLE S4  Phenotypic antimicrobial resistance profiles for the 198 *S*. Typhimurium/*S*. I:4,[5],12:i:− isolates from Vietnam. Antimicrobial drugs to which isolates are resistant are listed; drugs to which isolates have intermediate resistance are listed in brackets. Ap, ampicillin; Amc, amoxicillin-clavulanate; Cz, ceftazidime; Cx, ceftriaxone; Ch, chloramphenicol; Cp, ciprofloxacin; Gm, gentamicin; Nal, nalidixic acid; Of, ofloxacin; Tm, trimethoprim. Download TABLE S4, PDF file, 0.1 MB. Copyright © 2018 Mather et al. 2018 Mather et al. This content is distributed under the terms of the Creative Commons Attribution 4.0 International license.

10.1128/mBio.01056-18.8 TABLE S5  Acquired genes and single nucleotide polymorphisms conferring antimicrobial resistance, along with the relevant accession numbers, in the isolates from Vietnam and the context collection. Download TABLE S5, PDF file, 0.2 MB. Copyright © 2018 Mather et al. 2018 Mather et al. This content is distributed under the terms of the Creative Commons Attribution 4.0 International license.

We investigated the genomic context of the additional AMR genes in the MDR ST34 subgroup using long-read sequencing data generated using a Pacific Biosciences sequencing system; they were found to be located on a large (~246-kb) IncHI2 plasmid. This plasmid was similar in gene content and structure to plasmid pHXY0908 (accession number KM877269.1), which has been previously described in an *S*. Typhimurium isolate from chicken feces in China in 2009 (15). The pHXY0908 IncHI2 plasmid carried *oqxAB*, *blmS*, *sul1*, *ΔaadA2*, *dfrA12*, *aph3*, *sul3*, *aadA1a*, *cmlA2*, *aadA2*, *floR*, *sul2*, *hph*, *aac(3′)-IVa*, *aac(6′)-Ib-cr*, *blaOXA-1*, *catB3*, and *arr3*. The predicted phenotype of these organisms was resistance to fluoroquinolones, bleomycin, sulfonamides, trimethoprim, kanamycin, streptomycin, chloramphenicol, spectinomycin, florfenicol, hygromycin B, apramycin, beta-lactams, and rifampin. The six transitional subgroup isolates lay between the ancestral ST34 subgroup and the MDR ST34 subgroup and exhibited some characteristics of both of the other subgroups (Fig. 2). Of these six transitional isolates, five carried the same mercury resistance genes found in the archetypal ST34 monophasic clone, and three carried the *bla*_TEM-1_, *strAB*, *tetB*, and *sul2* genes, also typical of the monophasic European clone. In contrast, and comparable to the MDR ST34 subgroup, the transitional isolates were also genetically biphasic and carried the MDR IncHI2 plasmid (Table S6).

10.1128/mBio.01056-18.9 TABLE S6  Incompatibility (Inc) types of plasmids identified in the isolates from Vietnam and the context collection. Download TABLE S6, PDF file, 0.1 MB. Copyright © 2018 Mather et al. 2018 Mather et al. This content is distributed under the terms of the Creative Commons Attribution 4.0 International license.

**FIG 3:**
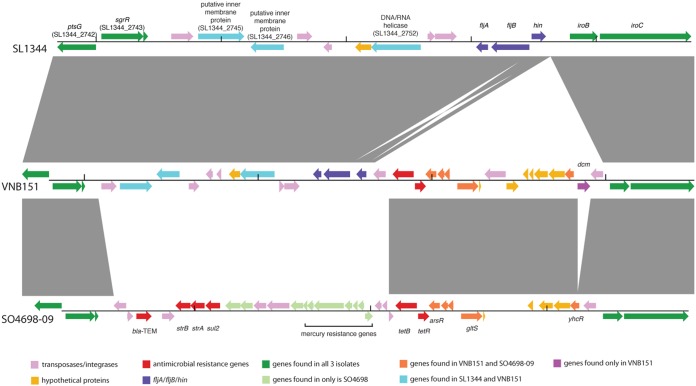
Multigenome comparison of the second flagellar region of *Salmonella* Typhimurium SL1344, isolate VNB151 from Vietnam, and monophasic *S*. Typhimurium variant *S*. I:4,[5],12:i:− SO4698-09. Arrows represent coding sequences for SL1344 and predicted coding sequences for VNB151 and SO4698-09; gray blocks indicate regions of genetic similarity between genomes. Minor differences in annotated coding sequences in regions with gray blocks reflect the predicted nature of the VNB151 and SO4698-09 annotation.

Two additional features distinguished the isolates in the ST34 MDR subgroup. First, these isolates were significantly associated with iNTS disease in HIV-infected Vietnamese individuals; 73% of human-derived isolates in this subgroup were from the blood of HIV-infected patients in comparison to 32% of the human-derived isolates from other clades (*P* = 0.001, Table 1). Second, while the MDR subgroup has arisen from monophasic ST34 isolates, the majority (26/31) of these isolates had an intact *fljBA* operon encoding a phase 2 flagellum and were phenotypically confirmed to be biphasic (Fig. 3). *S*. Typhimurium typically harbors two flagellin genes, *fliC* and *fljB*. These genes are regulated by the *hin* invertase so that only one flagellar antigen is expressed at any given time (16). In the pandemic ST34 monophasic *S*. I:4,[5],12:i:− ancestral clone, the *fljBA* operon has been deleted and replaced by a transposon (IS*26*-associated) carrying the AMR genes *bla*_TEM-1_, *strAB*, *sul2*, and *tetBR* and mercury resistance genes (12, 17). A fragment of this transposon remained in the majority of the ST34 MDR subgroup isolates, which included the *tetBR* genes, similarly inserted between *hin* and *iroB*, leaving *fljBA* intact (Fig. 3). While we could not reconstruct the precise sequence of events creating this reversion, the phylogenetic structure, bootstrap branch supports, and DNA sequence identity support that this second flagellar operon was reacquired by the MDR ST34 subgroup isolates from an alternative biphasic *S*. Typhimurium isolate (Fig. S2).

**TABLE 1:** Numbers of Vietnamese *S*. Typhimurium/*S*. I:4,[5],12:i:− isolates from human patients who are HIV infected or not HIV infected, excluding animal isolates

HIV infection status	No. (%) of isolates in clade:			
	Ancestral/monophasic ST34	MDR ST34	Transition ST34	Rest of tree
HIV infected	6 (40)	19 (73)	0 (0)	13 (32.5)
Not HIV infected	9 (60)	7 (27)	4 (100)	27 (67.5)
Total	15	26	4	40

Last, hypothesizing that these novel iNTS-associated ST34 biphasic organisms were adapted to cause an iNTS disease phenotype in humans (as has been reported for *S*. Typhimurium associated with iNTS in sub-Saharan Africa), we investigated potential genomic degradation. In a systematic scan for putative pseudogenes, we observed almost no evidence of genomic degradation in the ST34 MDR subgroup (Table S7), unlike iNTS-associated *S*. Typhimurium in sub-Saharan Africa. The only exception found in all 31 isolates was a frameshift in *pduT*, which was not present in the remaining Vietnamese ST34 isolates. This gene encodes a hypothetical propanediol utilization protein, and the frameshift would theoretically render this gene inactive.

10.1128/mBio.01056-18.10 TABLE S7  Genes reported as being associated with adaptation to an extraintestinal lifestyle in salmonellae by Okoro et al. (26) and Nuccio and Baumler (27) and the results for each from the pseudogene analysis on the 71 ST34 *S*. Typhimurium/*S*. I:4,[5],12:i:− genomes from Vietnam. Download TABLE S7, PDF file, 0.1 MB. Copyright © 2018 Mather et al. 2018 Mather et al. This content is distributed under the terms of the Creative Commons Attribution 4.0 International license.

## DISCUSSION

Our study outlines some important observations for *Salmonella* epidemiology in a global health context. Vietnam is a rapidly industrializing LMIC where the separation between humans and livestock species is less demarcated than in more developed countries. In this context, we observed either that distinct clades of *S*. Typhimurium and/or *S*. I:4,[5],12:i:− were composed predominantly of isolates from a single host species or that isolates from different host species were nonrandomly distributed within the clade. This observation suggests restricted interspecies transmission but should be interpreted with caution due to the limited overlap between sampling periods (humans, 2008 to 2013, and animals, 2011 to 2013). However, we found no significant association between sampling date and phylogeny in a tree containing only human isolates (lambda = 0.092, *P* =0.21), indicating that isolates did not cluster by year of isolation. Therefore, the observed clustering of isolates by host population was not confounded by the year of isolation.

Previously, it was assumed that the majority of NTS infections in humans arise through the food chain and are ultimately derived from animals (1). However, an increasing number of studies have shown that this scenario is more nuanced. In an industrialized setting, WGS of *S*. Typhimurium definitive type 104 (DT104) revealed that the major source of human NTS DT104 infections and the AMR of those infections was unlikely to be the local animal population (8). Additionally, a study from Kenya that compared NTS isolates from patients and from animals found that the organisms associated with the environment or food from within or near the homes of the patients were not significantly related to human isolates (18). A further investigation found that NTS isolates from patients were more comparable to NTS organisms obtained from asymptomatic household members than from the environmental or animal samples from the homes of index cases (6). Our work provides insight into the previously limited understanding of the transmission of iNTS and enterocolitis-causing *S*. Typhimurium and *S*. I:4,[5],12:i:− in Southeast Asia and identifies trends similar to those observed in other parts of the world.

There have been successive pandemics of *S*. Typhimurium, including both DT204c and DT104, in recent decades. The current pandemic is caused by the European clone of the monophasic ST34 *S*. Typhimurium variant, *S*. I:4,[5],12:i:−. This clone rose to prominence as a major cause of NTS disease in humans in Europe in the 2000s; pigs were identified as the most likely reservoir (19, 20). Since then, this organism has spread globally. ST34 was the second most common ST found within our Vietnamese isolates and is the most common *S*. Typhimurium ST currently isolated from humans and animals in China (21, 22). The pHXY0908-like plasmid found here within the MDR and transitional subgroups is also epidemic in China (15) and also associated with *S*. Typhimurium ST34 (21, 22). pHXY0908 additionally carries various metal tolerance genes conferring resistance to tellurite, and IncHI2 plasmids are facilitating the spread of *oqxAB* and *aac(6′)-Ib-cr* plasmid-mediated quinolone resistance determinants in NTS organisms. Here, we infrequently found IncHI2 plasmids outside the MDR and transitional ST34 subgroups. The exceptions to this IncHI2 plasmid distribution were a duck isolate from clade 1, a human isolate from clade 4, two Chinese ST19 isolates, and several isolates in the monophasic ST34 subgroup: nine Vietnamese isolates (human bloodstream infections, *n* = 3; pigs, *n* = 5; ducks, *n* = 1), five European isolates, and a Chinese ST34 isolate. However, in comparison to those in the ST34 MDR and the transitional subgroups, the plasmids identified in isolates in other clades carried fewer and/or a different complement of AMR genes (see Tables S5 and S6 in the supplemental material).

Our work describes a novel evolutionary pathway by which an emergent *Salmonella*, typically associated with noninvasive disease, has exploited an alternative human niche in HIV-infected individuals. This linkage of an enterocolitis-associated *Salmonella* in developed countries to one causing invasive disease in predominantly immunocompromised individuals in developing countries has been previously observed with Salmonella enterica serovar Enteritidis and non-ST34 *S*. Typhimurium subtypes (primarily ST313) in sub-Saharan Africa (2, 23). In the case of ST313, these biphasic organisms have undergone genome degradation comparable to that of Salmonella enterica serovar Typhi, acquired AMR genes on a virulence plasmid, and spread systemically in susceptible individuals (24). Here, a monophasic ST34 *S*. Typhimurium variant, *S*. I:4,[5],12:i:−, with an international distribution has reacquired a secondary flagellin gene and become associated with invasive disease in HIV-infected individuals in an industrializing country in Southeast Asia. Notably, and unlike ST313 in sub-Saharan Africa, this Vietnamese ST34 variant does not exhibit extensive evidence of genome degradation. The additional flagellin gene may possibly confer a virulence advantage in immunocompromised individuals, but further work exploiting suitable experimental systems, as has been performed for ST313 (3, 4), is required to test this hypothesis robustly. It is likely that the acquisition and maintenance of a broad-range MDR plasmid confer an advantage, due to the sustained prescribing of broad-spectrum antimicrobials to HIV-infected individuals in Vietnam. This series of events combines an evolving *Salmonella* clone with a global distribution, a pervasive Asian MDR plasmid, and a primary burden of disease in HIV-infected individuals. Although we have identified this new sublineage in Vietnam, it is likely not restricted to Southeast Asia. Recently, two *S*. Typhimurium ST34 isolates with similar IncHI2 plasmids were identified from two patients in Portugal, with no travel history to Asia and no report of foodborne outbreaks or recent contact with animals (25).

Our data suggest that conditions in Vietnam likely influenced the emergence of a new sublineage of *S*. Typhimurium. We predict that these conditions may be replicated in comparable LMICs, which may facilitate the emergence of new variants of pathogenic bacteria into human populations. These results demonstrate the incredible genomic plasticity, global mobility, and virulence potential of *S*. Typhimurium. The international circulation of these organisms combined with their ability to acquire AMR genes and to cause invasive disease in HIV-infected humans highlights the need for improved surveillance of bacterial pathogens in a One Health context. Our study highlights the impact of the global AMR crisis and adds a unique insight into the international epidemiology and emergent variants within Salmonella enterica.

## MATERIALS AND METHODS

### Ethics approval.

The scientific and ethics committees of the collaborating institutions and the Oxford Tropical Research Ethics Committee provided the ethical approvals for the studies that contributed organisms and data to this investigation.

### Vietnamese collection of *Salmonella* Typhimurium.

The data set for this study comprised 198 isolates of Salmonella enterica subsp. enterica serovar Typhimurium and *S*. I:4,[5],12:i:− isolated in Vietnam (see Table S1 in the supplemental material). These included 85 human-derived isolates: 36 from fecal samples taken from diarrheal patients, 41 from the blood of febrile patients, and eight from fecal samples taken from asymptomatic individuals. Additionally, 113 *Salmonella* Typhimurium and *S*. I:4,[5],12:i:− isolates isolated from the fecal material of asymptomatic animals (14 from chickens, 70 from ducks, and 29 from pigs) collected in the southern part of Vietnam from 2011 to 2013 were included. Details of the origins of these isolates can be found in Text S1 in the supplemental material.

10.1128/mBio.01056-18.1 TEXT S1  Supplemental methods. Download TEXT S1, PDF file, 0.1 MB. Copyright © 2018 Mather et al. 2018 Mather et al. This content is distributed under the terms of the Creative Commons Attribution 4.0 International license.

### MLST and genome sequencing.

S. Typhimurium and *S*. I:4,[5],12:i:− isolates were identified by multilocus sequence typing (MLST) prior to whole-genome sequencing (WGS). Genomic DNA was extracted using the Wizard genomic DNA purification kit (Promega, USA), and the *Salmonella* MLST alleles were PCR amplified and sequenced in both directions using BigDye Terminator v3 (Applied Biosystems, USA) followed by capillary sequencing on a 3130XL Genetic Analyzer (Applied Biosystems, USA). All sequences were manually trimmed to align to a reference sequence and were submitted to the S. enterica MLST database (http://mlst.warwick.ac.uk/mlst/dbs/Senterica) for allelic profile and molecular serotyping. For each confirmed *S*. Typhimurium and *S*. I:4,[5],12:i:− isolate, 2 µg of the extracted genomic DNA was subjected to WGS on an Illumina HiSeq2000 platform (San Diego, CA, USA) according to the manufacturer’s protocols to generate 100-bp paired-end reads.

### Antimicrobial susceptibility testing.

Antimicrobial susceptibility testing was performed on all confirmed *S*. Typhimurium and *S*. I:4,[5],12:i:− isolates on Mueller-Hinton agar using the disk diffusion method as recommended by Clinical and Laboratory Standards Institute (CLSI) guidelines (26); antimicrobial disks were purchased from Oxoid (Thermo Fisher Scientific, United Kingdom). Antimicrobial susceptibility testing was performed against ampicillin, amoxicillin-clavulanate, ceftazidime, ceftriaxone, chloramphenicol, ciprofloxacin, gentamicin, nalidixic acid, ofloxacin, and trimethoprim-sulfamethoxazole. Antimicrobial susceptibility was determined using the CLSI guidelines (26).

### Contextual collection of *Salmonella* Typhimurium/*S*. I:4,[5],12:i:− genome sequences.

To place the Vietnamese *S*. Typhimurium and *S*. I:4,[5],12:i:− isolates in context, we included an additional 220 *S*. Typhimurium and monophasic variant *S*. I:4,[5],12:i:− genomes isolated from humans and animals in Europe and China; full details of these isolates, sources, and accession numbers are shown in Table S2. Assemblies only were available for the Chinese and Danish isolates; these assemblies were shredded to generate 125-bp paired-end reads for each isolate to allow further analysis.

### Genomic analysis and phylogenetics.

The short reads of the 198 *S*. Typhimurium and *S*. I:4,[5],12:i:− genomes from Vietnam were mapped to the reference genome *S*. Typhimurium SL1344 (27, 28), composed of a chromosome and three plasmids (accession numbers FQ312003, HE654724, HE654725, and HE654726) using SMALT v0.7.4 (29), and single nucleotide polymorphisms (SNPs) were called using previously described methods (30). Variations in regions of mobile genetic elements and repeats, including prophages and plasmids, were removed. The genome sequence of *Salmonella* Enteritidis P125109 (chromosome accession number AM933172) was added as an outgroup and similarly mapped to SL1344. Putative recombination was removed from the alignment using Gubbins (31). Hierarchical clustering of the isolates using hierBAPS (11) was performed using the resultant nonrecombinogenic SNP alignment, generating five primary BAPS clusters. A phylogenetic tree was created from the nonrecombinogenic SNPs using RAxML (32) and rooted on *S*. Enteritidis P125109 in iTOL (33). Annotated assemblies of each genome were produced using the pipeline outlined in the work of Page et al. (34), which is described in Text S1 in the supplemental material.

A separate, larger tree was produced by mapping the short reads of all isolates (198 Vietnam isolates and the 220 context collection isolates) to the reference genome *S*. I:4,[5],12:i:− SO4698-09 (accession number PRJEB10340) (12). SNPs were called as previously described, and variation in prophage sequences, repeat regions, and the genomic island was removed. The tree was rooted on the outgroup *S*. Enteritidis P125109 and visualized, along with the country of origin, in iTOL.

### Assessment of mixing between animal and human isolates from Vietnam.

With 85 human-derived isolates, 113 animal-derived isolates, and five primary BAPS clusters, if the isolates were sampled from a common, well-mixed pool of salmonellae, the assumption would be that approximately 43% (85/198) of isolates in each of the five BAPS clusters would originate from humans. This null hypothesis was tested using Fisher’s exact test in R (35), with the *P* value computed using Monte Carlo simulation.

Further assessment of potential mixing between animal and human populations was undertaken for each individual BAPS clade while accounting for phylogenetic structure. Each BAPS clade was extracted from the larger phylogenetic tree using the drop.tip function of the APE package in R (36). For each of the five clades separately, the *D* value, a measure of phylogenetic signal, was calculated for the binary host population (animal/human) trait using the caper package in R (37). The estimated *D* value was evaluated as to whether or not it was significantly different both from random association (*D* = 1) and from the clustering expected under a Brownian evolution threshold model (*D* = 0). Assessment of potential confounding by date of isolation was performed as described in Text S1.

### Identification of antimicrobial resistance determinants.

The ResFinder (38) reference database was used with ARIBA (39) to identify acquired resistance genes, and the results were visualized using Phandango (40). Resistance due to SNPs in the *gyrA*, *gyrB*, *parC*, and *parE* genes was investigated by creating a database of these genes from the reference sequence of *S*. Typhimurium SL1344 and using this with ARIBA to identify SNPs that have been previously associated with resistance. There was a subgroup within the ST34 isolates that had a high number of AMR determinants, called the ST34 MDR (multidrug-resistant) subgroup.

To assess whether or not the isolates from the two clades comprising mainly isolates from ducks (clades 1 and 5) had significantly fewer AMR determinants than the Vietnamese isolates in other clades, the mean number of AMR determinants in isolates from clade 1 (0.66/isolate) and clade 5 (0/isolate) separately was compared to the mean number of AMR determinants of combined isolates from clades 2, 3, and 4 (9.96/isolate) using Mann-Whitney U tests.

### Identification of putative plasmids.

ARIBA (39) was used to identify the plasmid replicon types, using the PlasmidFinder database (41), in each of the Vietnam and context collection isolates.

### Genomic identification of monophasic or biphasic *S*. Typhimurium.

Genomic identification of biphasic or monophasic *S*. Typhimurium was performed by examining the mapped sequence read coverage of the 198 Vietnam isolates against the reference *S*. Typhimurium SL1344 in the genomic region around the *fljBA* locus. Isolates were classified as monophasic (deletion or partial deletion of the *fljBA* locus or presence of the A46T *fljA* or R140L *hin* SNP described by Ido et al. [42]), biphasic (no deletion of the *fljBA* locus), or possibly biphasic (biphasic−: intact *fljBA* locus but possible deletion of the regions around *hin*, a DNA invertase allowing phase switching). Genomic classification of the flagellar status of the Vietnam ST34 isolates was confirmed by laboratory phase switching methods detailed below. Four of the five biphasic− (all ST34) were confirmed as biphasic, and the other was confirmed as monophasic; in the case of any disagreements between the genomic prediction and the laboratory phase switching results, the laboratory results were used.

Isolates in the context collection from the work of Petrovska et al. (12) were labeled as monophasic or biphasic according to the classifications in Technical Appendix 1 from that publication. The other isolates from the context collection, including any from the work of Petrovska et al. for which serotyping data were not available, were classified as genetically monophasic or biphasic based on genomic analysis as described for the Vietnam isolates.

### Phase switching.

To identify monophasic and biphasic *S*. Typhimurium Vietnam ST34 variants, cell suspensions were agglutinated with H:i (phase 1 flagellin) and H:1 (phase 2 flagellin) antisera according to the manufacturer’s instructions (SSI Diagnostica, Hillerød, Denmark) (43). These results were further confirmed using phase-changing assays as described in Text S1 in the supplemental material.

### Long-read sequencing.

A biphasic ST34 isolate in the ST34 MDR subclade (VNB151) was additionally sequenced using the Pacific Biosciences platform (Menlo Park, CA, USA). Genomic DNA was phenol-chloroform extracted and was sequenced using 1 single-molecule, real-time (SMRT) cell and the P2-B6 chemistry. Sequence reads were assembled using the methods described in Text S1.

### Association of ST34 MDR subgroup with HIV infections.

To identify if there was an association of the ST34 MDR subclade with HIV-infected individuals, we assessed the numbers of isolates from HIV-infected patients in the ST34 MDR subclade versus the rest of the isolates in the tree, and compared these to the number of isolates not derived from HIV-infected patients using a chi-squared test, using only the human isolates from Vietnam.

### Identification of pseudogenes in ST34 isolates.

To determine whether or not the isolates in the ST34 MDR subclade demonstrated evidence of genome degradation as observed in other *Salmonella* isolates adapted to invasive disease (44), the presence of pseudogenes was investigated in the 71 Vietnamese ST34 isolates, as outlined in Text S1 in the supplemental material. Genes which were disrupted in the majority of ST34 MDR subgroup isolates but not found in other subgroup isolates were identified.

### Data availability.

Accession numbers for all genomes used in this study are available in Tables S1 and S2. The assembly for the biphasic ST34 isolate in the ST34 MDR subclade (VNB151) was submitted to the European Nucleotide Archive under accession number GCA_900166885.

## References

[1] Majowicz SE, Musto J, Scallan E, Angulo FJ, Kirk M, O’Brien SJ, Jones TF, Fazil A, Hoekstra RM, International Collaboration on Enteric Disease ‘Burden of Illness’ Studies 2010 The global burden of nontyphoidal *Salmonella* gastroenteritis. Clin Infect Dis 50:882–889. doi:10.1086/650733. 20158401

[2] Okoro CK, Kingsley RA, Connor TR, Harris SR, Parry CM, Al-Mashhadani MN, Kariuki S, Msefula CL, Gordon MA, de Pinna E, Wain J, Heyderman RS, Obaro S, Alonso PL, Mandomando I, MacLennan CA, Tapia MD, Levine MM, Tennant SM, Parkhill J, Dougan G 2012 Intracontinental spread of human invasive Salmonella Typhimurium pathovariants in sub-Saharan Africa. Nat Genet 44:1215–1221. doi:10.1038/ng.2423. 23023330 PMC3491877

[3] Parsons BN, Humphrey S, Salisbury AM, Mikoleit J, Hinton JC, Gordon MA, Wigley P 2013 Invasive non-typhoidal Salmonella typhimurium ST313 are not host-restricted and have an invasive phenotype in experimentally infected chickens. PLoS Negl Trop Dis 7:e2487. doi:10.1371/journal.pntd.0002487. 24130915 PMC3794976

[4] Ramachandran G, Panda A, Higginson EE, Ateh E, Lipsky MM, Sen S, Matson CA, Permala-Booth J, DeTolla LJ, Tennant SM 2017 Virulence of invasive Salmonella Typhimurium ST313 in animal models of infection. PLoS Negl Trop Dis 11:e0005697. doi:10.1371/journal.pntd.0005697. 28783750 PMC5559095

[5] Phu Huong Lan N, Le Thi Phuong T, Nguyen Huu H, Thuy L, Mather AE, Park SE, Marks F, Thwaites GE, Van Vinh Chau N, Thompson CN, Baker S 2016 Invasive non-typhoidal *Salmonella* infections in Asia: clinical observations, disease outcome and dominant serovars from an infectious disease hospital in Vietnam. PLoS Negl Trop Dis 10:e0004857. doi:10.1371/journal.pntd.0004857. 27513951 PMC4981332

[6] Kariuki S, Revathi G, Kariuki N, Kiiru J, Mwituria J, Muyodi J, Githinji JW, Kagendo D, Munyalo A, Hart CA 2006 Invasive multidrug-resistant non-typhoidal *Salmonella* infections in Africa: zoonotic or anthroponotic transmission? J Med Microbiol 55:585–591. doi:10.1099/jmm.0.46375-0. 16585646

[7] Krueger AL, Greene SA, Barzilay EJ, Henao O, Vugia D, Hanna S, Meyer S, Smith K, Pecic G, Hoefer D, Griffin PM 2014 Clinical outcomes of nalidixic acid, ceftriaxone, and multidrug-resistant nontyphoidal *Salmonella* infections compared with pansusceptible infections in FoodNet sites, 2006–2008. Foodborne Pathog Dis 11:335–341. doi:10.1089/fpd.2013.1642. 24617446

[8] Mather AE, Reid SWJ, Maskell DJ, Parkhill J, Fookes MC, Harris SR, Brown DJ, Coia JE, Mulvey MR, Gilmour MW, Petrovska L, de Pinna E, Kuroda M, Akiba M, Izumiya H, Connor TR, Suchard MA, Lemey P, Mellor DJ, Haydon DT, Thomson NR 2013 Distinguishable epidemics of multidrug-resistant Salmonella Typhimurium DT104 in different hosts. Science 341:1514–1517. doi:10.1126/science.1240578. 24030491 PMC4012302

[9] Nhung NT, Cuong NV, Thwaites G, Carrique-Mas J 2016 Antimicrobial usage and antimicrobial resistance in animal production in Southeast Asia: a review. Antibiotics 5:37. doi:10.3390/antibiotics5040037. PMC5187518 27827853

[10] Nguyen KV, Thi Do NT, Chandna A, Nguyen TV, Pham CV, Doan PM, Nguyen AQ, Thi Nguyen CK, Larsson M, Escalante S, Olowokure B, Laxminarayan R, Gelband H, Horby P, Thi Ngo HB, Hoang MT, Farrar J, Hien TT, Wertheim HF 2013 Antibiotic use and resistance in emerging economies: a situation analysis for Viet Nam. BMC Public Health 13:1158. doi:10.1186/1471-2458-13-1158. 24325208 PMC4116647

[11] Cheng L, Connor TR, Sirén J, Aanensen DM, Corander J 2013 Hierarchical and spatially explicit clustering of DNA sequences with BAPS software. Mol Biol Evol 30:1224–1228. doi:10.1093/molbev/mst028. 23408797 PMC3670731

[12] Petrovska L, Mather AE, AbuOun M, Branchu P, Harris SR, Connor T, Hopkins KL, Underwood A, Lettini AA, Page A, Bagnall M, Wain J, Parkhill J, Dougan G, Davies R, Kingsley RA 2016 Microevolution of monophasic Salmonella Typhimurium during epidemic, United Kingdom, 2005–2010. Emerg Infect Dis 22:617–624. doi:10.3201/eid2204.150531. 26982594 PMC4806966

[13] Qin Y, Hasman H, Aarestrup FM, Alwathnani HA, Rensing C 2014 Genome sequences of three highly copper-resistant Salmonella enterica subsp. I serovar Typhimurium strains isolated from pigs in Denmark. Genome Announc 2:e01334-14. doi:10.1128/genomeA.01334-14. 25540347 PMC4276825

[14] Cheng CK, Cheung MK, Nong W, Law PT, Qin J, Ling JM, Kam KM, Cheung WM, Kwan HS 2015 Next generation genome sequencing reveals phylogenetic clades with different level of virulence among Salmonella Typhimurium clinical human isolates in Hong Kong. BMC Genomics 16:688. doi:10.1186/s12864-015-1900-y. 26370680 PMC4570558

[15] Li L, Liao X, Yang Y, Sun J, Li L, Liu B, Yang S, Ma J, Li X, Zhang Q, Liu Y 2013 Spread of *oqxAB* in *Salmonella enterica* serotype Typhimurium predominantly by IncHI2 plasmids. J Antimicrob Chemother 68:2263–2268. doi:10.1093/jac/dkt209. 23737490

[16] Zieg J, Silverman M, Hilmen M, Simon M 1977 Recombinational switch for gene expression. Science 196:170–172. doi:10.1126/science.322276. 322276

[17] Lucarelli C, Dionisi AM, Filetici E, Owczarek S, Luzzi I, Villa L 2012 Nucleotide sequence of the chromosomal region conferring multidrug resistance (R-type ASSuT) in Salmonella Typhimurium and monophasic Salmonella Typhimurium strains. J Antimicrob Chemother 67:111–114. doi:10.1093/jac/dkr391. 21990047

[18] Kariuki S, Revathi G, Gakuya F, Yamo V, Muyodi J, Hart CA 2002 Lack of clonal relationship between non-typhi *Salmonella* strain types from humans and those isolated from animals living in close contact. FEMS Immunol Med Microbiol 33:165–171. doi:10.1111/j.1574-695X.2002.tb00587.x. 12110478

[19] Antunes P, Mourão J, Pestana N, Peixe L 2011 Leakage of emerging clinically relevant multidrug-resistant *Salmonella* clones from pig farms. J Antimicrob Chemother 66:2028–2032. doi:10.1093/jac/dkr228. 21697179

[20] Hopkins KL, Kirchner M, Guerra B, Granier SA, Lucarelli C, Porrero MC, Jakubczak A, Threlfall EJ, Mevius DJ 2010 Multiresistant Salmonella enterica serovar 4,[5],12:i:- in Europe: a new pandemic strain? Euro Surveill 15:19580. doi:10.2807/ese.15.22.19580-en. 20546690

[21] Wong MHY, Yan M, Chan EWC, Liu LZ, Kan B, Chen S 2013 Expansion of *Salmonella enterica* serovar Typhimurium ST34 clone carrying multiple resistance determinants in China. Antimicrob Agents Chemother 57:4599–4601. doi:10.1128/AAC.01174-13. 23796940 PMC3754352

[22] Sun J, Ke B, Huang Y, He D, Li X, Liang Z, Ke C 2014 The molecular epidemiological characteristics and genetic diversity of Salmonella Typhimurium in Guangdong, China, 2007–2011. PLoS One 9:e113145. doi:10.1371/journal.pone.0113145. 25380053 PMC4224511

[23] Feasey NA, Hadfield J, Keddy KH, Dallman TJ, Jacobs J, Deng X, Wigley P, Barquist L, Langridge GC, Feltwell T, Harris SR, Mather AE, Fookes M, Aslett M, Msefula C, Kariuki S, Maclennan CA, Onsare RS, Weill FX, Le Hello S, Smith AM, McClelland M, Desai P, Parry CM, Cheesbrough J, French N, Campos J, Chabalgoity JA, Betancor L, Hopkins KL, Nair S, Humphrey TJ, Lunguya O, Cogan TA, Tapia MD, Sow SO, Tennant SM, Bornstein K, Levine MM, Lacharme-Lora L, Everett DB, Kingsley RA, Parkhill J, Heyderman RS, Dougan G, Gordon MA, Thomson NR 2016 Distinct *Salmonella* enteritidis lineages associated with enterocolitis in high-income settings and invasive disease in low-income settings. Nat Genet 48:1211–1217. doi:10.1038/ng.3644. 27548315 PMC5047355

[24] Kingsley RA, Msefula CL, Thomson NR, Kariuki S, Holt KE, Gordon MA, Harris D, Clarke L, Whitehead S, Sangal V, Marsh K, Achtman M, Molyneux ME, Cormican M, Parkhill J, MacLennan CA, Heyderman RS, Dougan G 2009 Epidemic multiple drug resistant Salmonella Typhimurium causing invasive disease in sub-Saharan Africa have a distinct genotype. Genome Res 19:2279–2287. doi:10.1101/gr.091017.109. 19901036 PMC2792184

[25] Campos J, Mourão J, Marçal S, Machado J, Novais C, Peixe L, Antunes P 2016 Clinical Salmonella Typhimurium ST34 with metal tolerance genes and an IncHI2 plasmid carrying *oqxAB-aac*(*6′*)-*Ib-cr* from Europe. J Antimicrob Chemother 71:843–845. doi:10.1093/jac/dkv409. 26679244

[26] Clinical and Laboratory Standards Institute 2014 Standards for antimicrobial susceptibility testing; 24th informational supplement. CLSI document m100-S24 Clinical and Laboratory Standards Institute, Wayne, PA.

[27] Hoiseth SK, Stocker BA 1981 Aromatic-dependent *Salmonella* typhimurium are non-virulent and effective as live vaccines. Nature 291:238–239. doi:10.1038/291238a0. 7015147

[28] Kröger C, Dillon SC, Cameron AD, Papenfort K, Sivasankaran SK, Hokamp K, Chao Y, Sittka A, Hébrard M, Händler K, Colgan A, Leekitcharoenphon P, Langridge GC, Lohan AJ, Loftus B, Lucchini S, Ussery DW, Dorman CJ, Thomson NR, Vogel J, Hinton JC 2012 The transcriptional landscape and small RNAs of *Salmonella enterica* serovar Typhimurium. Proc Natl Acad Sci U S A 109:E1277–E1286. doi:10.1073/pnas.1201061109. 22538806 PMC3356629

[29] Wellcome Trust Sanger Institute SMALT: pairwise sequence alignment program. Wellcome Trust Sanger Institute, Hinxton, United Kingdom http://www.sanger.ac.uk/resources/software/smalt/.

[30] Makendi C, Page AJ, Wren BW, Le Thi Phuong T, Clare S, Hale C, Goulding D, Klemm EJ, Pickard D, Okoro C, Hunt M, Thompson CN, Phu Huong Lan N, Tran Do Hoang N, Thwaites GE, Le Hello S, Brisabois A, Weill FX, Baker S, Dougan G 2016 A phylogenetic and phenotypic analysis of *Salmonella enterica* serovar Weltevreden, an emerging agent of diarrheal disease in tropical regions. PLoS Negl Trop Dis 10:e0004446. doi:10.1371/journal.pntd.0004446. 26867150 PMC4750946

[31] Croucher NJ, Page AJ, Connor TR, Delaney AJ, Keane JA, Bentley SD, Parkhill J, Harris SR 2015 Rapid phylogenetic analysis of large samples of recombinant bacterial whole genome sequences using Gubbins. Nucleic Acids Res 43:e15. doi:10.1093/nar/gku1196. 25414349 PMC4330336

[32] Stamatakis A 2006 RAxML-VI-HPC: maximum likelihood-based phylogenetic analyses with thousands of taxa and mixed models. Bioinformatics 22:2688–2690. doi:10.1093/bioinformatics/btl446. 16928733

[33] Letunic I, Bork P 2016 Interactive tree of life (iTOL) v3: an online tool for the display and annotation of phylogenetic and other trees. Nucleic Acids Res 44:W242–W245. doi:10.1093/nar/gkw290. 27095192 PMC4987883

[34] Page AJ, De Silva N, Hunt M, Quail MA, Parkhill J, Harris SR, Otto TD, Keane JA 2016 Robust high throughput prokaryote *de novo* assembly and improvement pipeline for Illumina data. Microb Genom 2:e000083. doi:10.1099/mgen.0.000083. 28348874 PMC5320598

[35] R Core Team 2016 R: a language and environment for statistical computing. R Foundation for Statistical Computing, Vienna, Austria https://www.R-project.org/.

[36] Paradis E, Claude J, Strimmer K 2004 APE: analyses of phylogenetics and evolution in R language. Bioinformatics 20:289–290. doi:10.1093/bioinformatics/btg412. 14734327

[37] Orme D, Freckleton R, Thomas G, Petzoldt T, Fritz S, Isaac N, Pearse W 2013 caper: comparative analyses of phylogenetics and evolution in R, v0.5.2. https://CRAN.R-project.org/package=caper.

[38] Zankari E, Hasman H, Cosentino S, Vestergaard M, Rasmussen S, Lund O, Aarestrup FM, Larsen MV 2012 Identification of acquired antimicrobial resistance genes. J Antimicrob Chemother 67:2640–2644. doi:10.1093/jac/dks261. 22782487 PMC3468078

[39] Hunt M, Mather AE, Sánchez-Busó L, Page AJ, Parkhill J, Keane JA, Harris SR 2017 ARIBA: rapid antimicrobial resistance genotyping directly from sequencing reads. Microb Genom 3:e000131. doi:10.1099/mgen.0.000131. 29177089 PMC5695208

[40] Hadfield J, Croucher NJ, Goater RJ, Abudahab K, Aanensen DM, Harris SR 2017 Phandango: an interactive viewer for bacterial population genomics. Bioinformatics 34:292–293. doi:10.1093/bioinformatics/btx610. PMC5860215 29028899

[41] Carattoli A, Zankari E, García-Fernández A, Voldby Larsen M, Lund O, Villa L, Møller Aarestrup F, Hasman H 2014 *In silico* detection and typing of plasmids using PlasmidFinder and plasmid multilocus sequence typing. Antimicrob Agents Chemother 58:3895–3903. doi:10.1128/AAC.02412-14. 24777092 PMC4068535

[42] Ido N, Lee K, Iwabuchi K, Izumiya H, Uchida I, Kusumoto M, Iwata T, Ohnishi M, Akiba M 2014 Characteristics of *Salmonella enterica* serovar 4,[5],12:i:- as a monophasic variant of serovar Typhimurium. PLoS One 9:e104380. doi:10.1371/journal.pone.0104380. 25093666 PMC4122451

[43] International Organization for Standardization 2014 ISO/TR 6579-3:2014. Microbiology of the food chain. Horizontal method for the detection, enumeration and serotyping of Salmonella. Part 3: guidelines for serotyping of *Salmonella* spp. International Organization for Standardization, Geneva, Switzerland.

[44] Okoro CK, Barquist L, Connor TR, Harris SR, Clare S, Stevens MP, Arends MJ, Hale C, Kane L, Pickard DJ, Hill J, Harcourt K, Parkhill J, Dougan G, Kingsley RA 2015 Signatures of adaptation in human invasive Salmonella Typhimurium ST313 populations from sub-Saharan Africa. PLoS Negl Trop Dis 9:e0003611. doi:10.1371/journal.pntd.0003611. 25803844 PMC4372345

