# Supplementary material for: New Variant of Multidrug-Resistant Salmonella enterica Serovar Typhimurium Associated with Invasive Disease in Immunocompromised Patients in Vietnam
Source: mBio. 2018 Sep 4;9(5):e01056-18. doi: 10.1128/mBio.01056-18 (PMC6123440; doi:10.1128/mBio.01056-18)
Supplement: TABLE S1 [file mbo004184053st1.pdf]

Table S1. Details and epidemiological data for the 198 *Salmonella* Typhimurium/*Salmonella* I:4,[5],12:i:- isolates from Vietnam.

Sample	Accession	ST	Species	Year	HIV status*	Flagellar status	Infection type	BAPS group
71_G_169	ERS529910	19	Chicken	2012	0	monophasic	asymptomatic	4
71_H_083	ERS529923	19	Pig	2012	0	biphasic	asymptomatic	4
71_H_084	ERS529924	19	Pig	2012	0	biphasic	asymptomatic	4
71_H_085	ERS529922	19	Pig	2012	0	biphasic	asymptomatic	4
71_H_228	ERS529993	19	Pig	2012	0	biphasic	asymptomatic	4
71_H_243	ERS529925	19	Pig	2012	0	biphasic	asymptomatic	4
71_V_080	ERS529616	19	Duck	2012	0	biphasic	asymptomatic	1
71_V_097	ERS529621	19	Duck	2012	0	biphasic	asymptomatic	1
71_V_115	ERS529622	19	Duck	2012	0	biphasic	asymptomatic	1
71_V_330	ERS529623	19	Duck	2012	0	biphasic	asymptomatic	1
71_V_385	ERS529628	19	Duck	2012	0	biphasic	asymptomatic	1
71_V_386	ERS529629	19	Duck	2012	0	biphasic	asymptomatic	1
71_V_387	ERS529625	19	Duck	2012	0	biphasic	asymptomatic	1
71_V_389	ERS529624	19	Duck	2012	0	biphasic	asymptomatic	1
71_V_465	ERS529631	19	Duck	2012	0	biphasic	asymptomatic	1
71_V_466	ERS529630	19	Duck	2012	0	biphasic	asymptomatic	1
71_V_478	ERS529634	19	Duck	2012	0	biphasic	asymptomatic	1
71_V_479	ERS529633	19	Duck	2012	0	biphasic	asymptomatic	1
71_V_480	ERS529632	19	Duck	2012	0	biphasic	asymptomatic	1
72_H_332	ERS529928	19	Pig	2012	0	biphasic	asymptomatic	3
72_V_054	ERS529635	19	Duck	2012	0	biphasic	asymptomatic	1
72_V_055	ERS529636	19	Duck	2012	0	biphasic	asymptomatic	1
72_V_082	ERS529638	19	Duck	2012	0	biphasic	asymptomatic	1
72_V_083	ERS529639	19	Duck	2012	0	biphasic	asymptomatic	1
72_V_085	ERS529637	19	Duck	2012	0	biphasic	asymptomatic	1
72_V_111	ERS529640	19	Duck	2012	0	biphasic	asymptomatic	1
72_V_228	ERS529641	19	Duck	2012	0	biphasic	asymptomatic	1
72_V_267	ERS529642	19	Duck	2012	0	biphasic	asymptomatic	1
72_V_269	ERS529643	19	Duck	2012	0	biphasic	asymptomatic	1
72_V_282	ERS529644	19	Duck	2012	0	biphasic	asymptomatic	1
72-G-120	ERS529912	19	Chicken	2012	0	biphasic	asymptomatic	1
73_H_001	ERS529929	19	Pig	2012	0	biphasic	diarrhoea	4
73_H_110	ERS529930	19	Pig	2012	0	biphasic	asymptomatic	4
73_H_111	ERS529931	19	Pig	2012	0	biphasic	asymptomatic	4
73_H_112	ERS529932	19	Pig	2012	0	biphasic	diarrhoea	4
73_H_170	ERS529934	19	Pig	2012	0	biphasic	asymptomatic	4

73_V_001	ERS529645	19	Duck	2012	0	biphasic	asymptomatic	1
73_V_020	ERS529875	19	Duck	2012	0	biphasic	asymptomatic	1
73_V_023	ERS529876	19	Duck	2012	0	biphasic	asymptomatic	1
73_V_038	ERS529878	19	Duck	2012	0	biphasic	asymptomatic	1
73_V_039	ERS529877	19	Duck	2012	0	biphasic	asymptomatic	1
73_V_065	ERS529879	19	Duck	2012	0	biphasic	asymptomatic	1
73_V_110	ERS529880	19	Duck	2012	0	biphasic	asymptomatic	1
73_V_113	ERS529881	19	Duck	2012	0	biphasic	asymptomatic	1
73_V_114	ERS529882	19	Duck	2012	0	biphasic	asymptomatic	1
73_V_168	ERS529883	19	Duck	2012	0	biphasic	asymptomatic	1
73_V_253	ERS529884	19	Duck	2012	0	biphasic	asymptomatic	1
73_V_282	ERS529885	19	Duck	2012	0	biphasic	asymptomatic	1
73_V_283	ERS529887	19	Duck	2012	0	biphasic	asymptomatic	1
73_V_285	ERS529888	19	Duck	2012	0	biphasic	asymptomatic	1
73_V_286	ERS529886	19	Duck	2012	0	biphasic	asymptomatic	1
73_V_320	ERS529889	19	Duck	2012	0	biphasic	asymptomatic	1
73_V_321	ERS529890	19	Duck	2012	0	biphasic	asymptomatic	1
73_V_322	ERS529891	19	Duck	2012	0	biphasic	asymptomatic	1
73_V_335	ERS529893	19	Duck	2012	0	biphasic	asymptomatic	1
73_V_336	ERS529892	19	Duck	2012	0	biphasic	asymptomatic	1
73_V_364	ERS529894	19	Duck	2012	0	biphasic	asymptomatic	4
73_V_423	ERS529897	19	Duck	2012	0	biphasic	asymptomatic	1
73_V_425	ERS529896	19	Duck	2012	0	biphasic	asymptomatic	1
73_V_426	ERS529898	19	Duck	2012	0	biphasic	asymptomatic	1
74_G_339	ERS529615	19	Chicken	2012	0	biphasic	asymptomatic	4
74_H_047	ERS529937	19	Pig	2012	0	biphasic	asymptomatic	4
74_V_100	ERS529899	19	Duck	2012	0	biphasic	asymptomatic	1
74_V_101	ERS529900	19	Duck	2012	0	biphasic	asymptomatic	1
74_V_102	ERS529901	19	Duck	2012	0	biphasic	asymptomatic	1
74_V_202	ERS529903	19	Duck	2012	0	biphasic	asymptomatic	1
74_V_217	ERS529904	19	Duck	2012	0	biphasic	asymptomatic	1
74_V_310	ERS529906	19	Duck	2012	0	biphasic	asymptomatic	1
74_V_368	ERS529907	19	Duck	2012	0	biphasic	asymptomatic	1
74_V_418	ERS529909	19	Duck	2012	0	biphasic	asymptomatic	1
74_V_419	ERS529908	19	Duck	2012	0	biphasic	asymptomatic	1
Hue_59	ERS529955	19	Human	2010	0	biphasic	diarrhoea	4
KH_69	ERS529957	19	Human	2010	0	biphasic	diarrhoea	4
VNB1403	ERS126814	19	Human	2011	0	biphasic	blood stream infection	1
VNB1428	ERS126826	19	Human	2011	0	biphasic	blood stream infection	4
VNB148	ERS126799	19	Human	2008	1	biphasic	blood stream infection	3

VNB170	ERS126823	19	Human	2008	1	biphasic	blood stream infection	4
VNB184	ERS126859	19	Human	2008	1	biphasic	blood stream infection	4
VNB1870	ERS529945	19	Human	2012	1	biphasic	blood stream infection	1
VNB198	ERS126776	19	Human	2008	1	biphasic	blood stream infection	3
VNB2175	ERS529948	19	Human	2012	1	biphasic	blood stream infection	4
VNB596	ERS126824	19	Human	2009	1	biphasic	blood stream infection	3
VNB652	ERS126848	19	Human	2009	1	biphasic	blood stream infection	3
VNB664	ERS126860	19	Human	2009	1	biphasic	blood stream infection	3
VNB68	ERS126805	19	Human	2008	1	monophasic	blood stream infection	4
VNB773	ERS126813	19	Human	2009	1	biphasic	blood stream infection	3
VNS10068	ERS126794	19	Human	2009	0	biphasic	diarrhoea	4
VNS10137	ERS126818	19	Human	2009	0	biphasic	diarrhoea	3
VNS121 DQT	ERS126786	19	Human	1998	0	biphasic	asymptomatic	4
VNS165 VDQ	ERS126798	19	Human	1997	0	biphasic	asymptomatic	4
VNS20018	ERS126807	19	Human	2009	0	biphasic	diarrhoea	4
VNS20057	ERS126819	19	Human	2009	0	biphasic	diarrhoea	4
VNS20207	ERS126784	19	Human	2009	0	biphasic	diarrhoea	4
VNS20277	ERS126808	19	Human	2009	0	biphasic	diarrhoea	4
VNS20278	ERS126820	19	Human	2009	0	biphasic	diarrhoea	4
VNS20337	ERS126832	19	Human	2009	0	biphasic	diarrhoea	4
VNS20480	ERS126844	19	Human	2010	0	biphasic	diarrhoea	4
VNS30012	ERS126856	19	Human	2009	0	biphasic	diarrhoea	4
VNS30015	ERS126773	19	Human	2009	0	biphasic	diarrhoea	4
VNS30099	ERS126785	19	Human	2009	0	biphasic	diarrhoea	4
VNS30161	ERS126821	19	Human	2009	0	biphasic	diarrhoea	4
VNS30267	ERS126845	19	Human	2009	0	biphasic	diarrhoea	4
VNSC2047	ERS126834	19	Human	2010	0	biphasic	asymptomatic	3
VNSC2235	ERS126858	19	Human	2010	0	biphasic	asymptomatic	4
71_G_450	ERS529911	34	Chicken	2012	0	monophasic	asymptomatic	2
71_H_034	ERS529918	34	Pig	2012	0	biphasic	asymptomatic	2
71_H_035	ERS529917	34	Pig	2012	0	biphasic	asymptomatic	2
71_H_051	ERS529921	34	Pig	2012	0	monophasic	asymptomatic	2
71_H_052	ERS529920	34	Pig	2012	0	monophasic	asymptomatic	2
71_H_053	ERS529919	34	Pig	2012	0	monophasic	asymptomatic	2
71_H_455	ERS529991	34	Pig	2012	0	biphasic	asymptomatic	2
72_H_033	ERS529926	34	Pig	2012	0	biphasic	asymptomatic	2
72_H_265	ERS529927	34	Pig	2012	0	monophasic	asymptomatic	2
73_G_047	ERS529914	34	Chicken	2012	0	monophasic	asymptomatic	2
73_G_049	ERS529915	34	Chicken	2012	0	monophasic	asymptomatic	2

73_G_050	ERS529916	34	Chicken	2012	0	monophasic	asymptomatic	2
73_G_051	ERS529913	34	Chicken	2012	0	monophasic	asymptomatic	2
73_H_129	ERS529933	34	Pig	2012	0	monophasic	asymptomatic	2
73_H_172	ERS529935	34	Pig	2012	0	monophasic	asymptomatic	2
73_H_243	ERS529936	34	Pig	2012	0	monophasic	asymptomatic	2
74_H_072	ERS529938	34	Pig	2012	0	monophasic	diarrhoea	2
74_H_125	ERS529939	34	Pig	2012	0	biphasic	diarrhoea	2
74_H_126	ERS529940	34	Pig	2012	0	biphasic	asymptomatic	2
74_H_253	ERS529941	34	Pig	2012	0	monophasic	asymptomatic	2
74_H_256	ERS529942	34	Pig	2012	0	monophasic	asymptomatic	2
74_V_235	ERS529905	34	Duck	2012	0	monophasic	asymptomatic	2
CT49_2	ERS529987	34	Chicken	2012	0	biphasic	asymptomatic	2
CT55_1	ERS529988	34	Chicken	2012	0	monophasic	asymptomatic	2
CT69_2	ERS529990	34	Chicken	2013	0	monophasic	asymptomatic	2
Hue_11	ERS529954	34	Human	2009	0	biphasic	diarrhoea	2
MT13C.2.2	ERS529986	34	Chicken	2012	0	monophasic	asymptomatic	2
VNB1140	ERS126861	34	Human	2010	1	biphasic	blood stream infection	2
VNB1166	ERS126778	34	Human	2010	1	monophasic	blood stream infection	2
VNB1222	ERS126790	34	Human	2010	1	biphasic	blood stream infection	2
VNB1264	ERS126802	34	Human	2011	1	biphasic	blood stream infection	2
VNB1436	ERS126838	34	Human	2011	1	monophasic	blood stream infection	2
VNB1479	ERS126850	34	Human	2011	1	monophasic	blood stream infection	2
VNB1505	ERS126862	34	Human	2011	1	monophasic	blood stream infection	2
VNB151	ERS126811	34	Human	2008	1	biphasic	blood stream infection	2
VNB1701	ERS126817	34	Human	2012	1	monophasic	blood stream infection	2
VNB176	ERS126835	34	Human	2008	1	biphasic	blood stream infection	2
VNB177	ERS126847	34	Human	2008	1	biphasic	blood stream infection	2
VNB1779	ERS529943	34	Human	2012	1	biphasic	blood stream infection	2
VNB1792	ERS529944	34	Human	2012	1	monophasic	blood stream infection	2
VNB2140	ERS529947	34	Human	2012	1	biphasic	blood stream infection	2
VNB2315	ERS529950	34	Human	2013	1	monophasic	blood stream infection	2
VNB2605	ERS529952	34	Human	2013	1	monophasic	blood stream infection	2
VNB455	ERS126788	34	Human	2009	1	monophasic	blood stream infection	2
VNB541	ERS126800	34	Human	2009	1	biphasic	blood stream infection	2
VNB589	ERS126812	34	Human	2009	1	biphasic	blood stream infection	2
VNB617	ERS126836	34	Human	2009	1	biphasic	blood stream infection	2
VNB692	ERS126777	34	Human	2009	1	biphasic	blood stream infection	2
VNB712	ERS126789	34	Human	2009	0	biphasic	blood stream infection	2
VNB745	ERS126801	34	Human	2009	1	monophasic	blood stream infection	2
VNB802	ERS126825	34	Human	2010	1	biphasic	blood stream infection	2

VNB845	ERS126837	34	Human	2010	1	biphasic	blood stream infection	2
VNB922	ERS126849	34	Human	2010	1	biphasic	blood stream infection	2
VNS10045	ERS126770	34	Human	2009	0	biphasic	diarrhoea	2
VNS10052	ERS126782	34	Human	2009	0	biphasic	diarrhoea	2
VNS10146	ERS126830	34	Human	2009	0	biphasic	diarrhoea	2
VNS10182	ERS126842	34	Human	2009	0	biphasic	diarrhoea	2
VNS10314	ERS126787	34	Human	2010	0	monophasic	diarrhoea	2
VNS10413	ERS126771	34	Human	2010	0	biphasic	diarrhoea	2
VNS20005	ERS126783	34	Human	2009	0	biphasic	diarrhoea	2
VNS20007	ERS126795	34	Human	2009	0	monophasic	diarrhoea	2
VNS20081	ERS126863	34	Human	2009	0	biphasic	diarrhoea	2
VNS20099	ERS126855	34	Human	2009	0	monophasic	diarrhoea	2
VNS20101	ERS126843	34	Human	2009	0	monophasic	diarrhoea	2
VNS20235	ERS126796	34	Human	2009	0	monophasic	diarrhoea	2
VNS30144	ERS126809	34	Human	2009	0	biphasic	diarrhoea	2
VNS30243	ERS126833	34	Human	2009	0	monophasic	diarrhoea	2
VNS30356	ERS126857	34	Human	2009	0	monophasic	diarrhoea	2
VNS30385	ERS126774	34	Human	2010	0	biphasic	diarrhoea	2
VNSC2045	ERS126822	34	Human	2010	0	monophasic	asymptomatic	2
VNSC2191	ERS126846	34	Human	2010	0	monophasic	asymptomatic	2
71_H_114	ERS529992	36	Pig	2012	0	biphasic	asymptomatic	3
72-G-232	ERS529997	36	Chicken	2012	0	biphasic	asymptomatic	3
VNSC2362	ERS126775	36	Human	2010	0	biphasic	asymptomatic	3
VNSC2442	ERS126810	36	Human	2010	0	biphasic	asymptomatic	3
VNS20150	ERS126772	99	Human	2009	0	monophasic	diarrhoea	3
Hue_98	ERS529956	313	Human	2011	0	biphasic	diarrhoea	3
71_V_202	ERS529994	1544	Duck	2012	0	biphasic	asymptomatic	5
71_V_204	ERS529995	1544	Duck	2012	0	biphasic	asymptomatic	5
71_V_313	ERS529996	1544	Duck	2012	0	biphasic	asymptomatic	5
74_G_043	ERS529613	1544	Chicken	2012	0	biphasic	asymptomatic	5
74_V_129	ERS529902	1544	Duck	2012	0	biphasic	asymptomatic	5
VNB2200	ERS529949	1544	Human	2012	1	biphasic	blood stream infection	5
VNB2339	ERS529951	1544	Human	2013	1	biphasic	blood stream infection	5
VNDSal1	ERS126779	1544	Duck	2011	0	biphasic	asymptomatic	5
VNDSal2	ERS126791	1544	Duck	2011	0	biphasic	asymptomatic	5
VNDSal3	ERS126803	1544	Duck	2011	0	biphasic	asymptomatic	5
VNDSal4	ERS126815	1544	Duck	2011	0	biphasic	asymptomatic	5
VNDSal5	ERS126827	1544	Duck	2011	0	biphasic	asymptomatic	5
VNDSal6	ERS126839	1544	Duck	2011	0	biphasic	asymptomatic	5
VNDSal7	ERS126851	1544	Duck	2011	0	biphasic	asymptomatic	5

VNDSal8	ERS126831	1544	Duck	2011	0	biphasic	asymptomatic	5
VNS10124	ERS126806	1544	Human	2009	0	biphasic	diarrhoea	5
73_V_381	ERS529895	2381	Duck	2012	0	biphasic	asymptomatic	1

* 1 = HIV-infected; 0 = not HIV-infected
