# Supplementary material for: New Variant of Multidrug-Resistant Salmonella enterica Serovar Typhimurium Associated with Invasive Disease in Immunocompromised Patients in Vietnam
Source: mBio. 2018 Sep 4;9(5):e01056-18. doi: 10.1128/mBio.01056-18 (PMC6123440; doi:10.1128/mBio.01056-18)
Supplement: FIG S1 [file mbo004184053sf1.pdf]

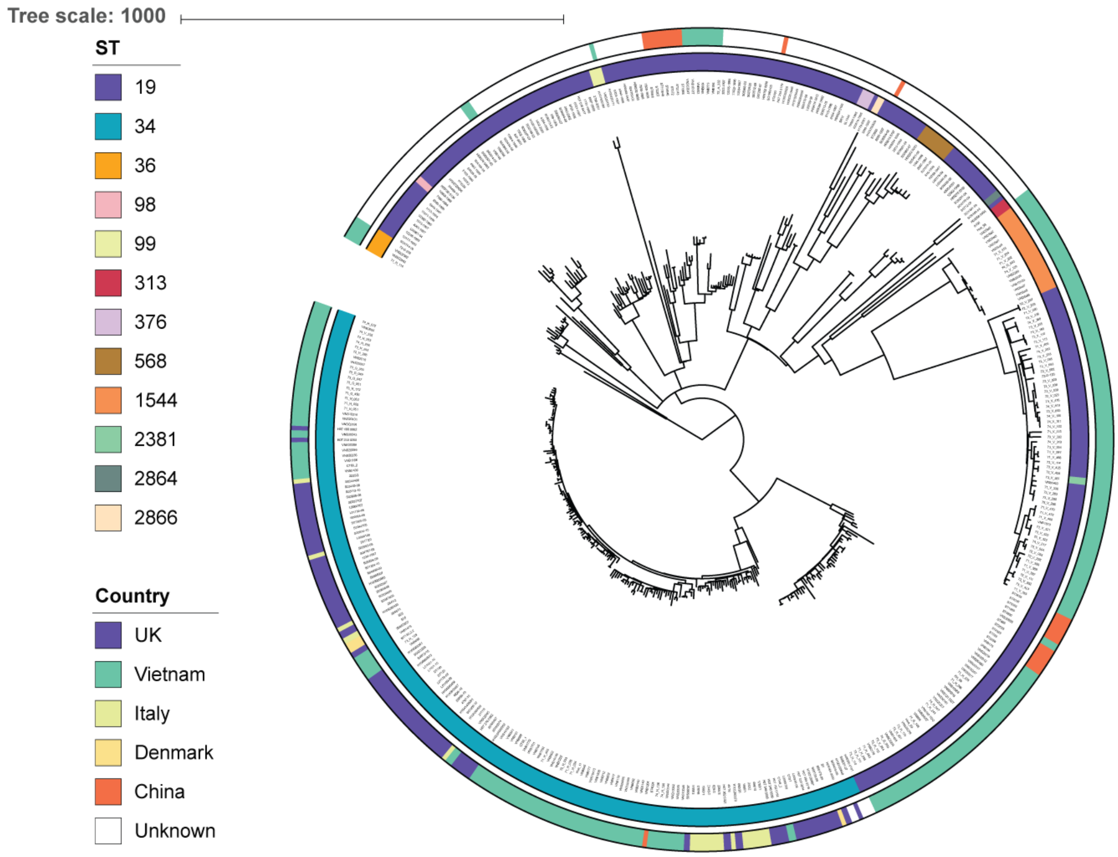

Fig. S1. Maximum likelihood phylogeny of *S.* Typhimurium/*S.* I:4,[5],12:i:- isolates from animals and humans mapped to the monophasic reference SO4698-09, including 198 from Vietnam and 220 from other countries with multi-locus sequence type (ST) and the available metadata on country of origin. Scale bar represents the number of non-recombinogenic single nucleotide polymorphisms per branch.
