# Supplementary material for: New Variant of Multidrug-Resistant Salmonella enterica Serovar Typhimurium Associated with Invasive Disease in Immunocompromised Patients in Vietnam
Source: mBio. 2018 Sep 4;9(5):e01056-18. doi: 10.1128/mBio.01056-18 (PMC6123440; doi:10.1128/mBio.01056-18)
Supplement: TABLE S2 [file mbo004184053st2.pdf]

Table S2. Details and epidemiological data for isolates in the context collection.

Sample	Accession	ST	Species	Year	Country	Flagellar status	Reference
ST111849	JRZX00000000	19	Human	1995	China	biphasic	Cheng et al. 2015
ST1489	JRZO00000000	19	Human	2006	China	biphasic	Cheng et al. 2015
ST1660	JRZS00000000	19	Human	2006	China	biphasic	Cheng et al. 2015
ST2143	JRZM00000000	19	Human	2005	China	biphasic	Cheng et al. 2015
ST2286	JRZR00000000	19	Human	2006	China	biphasic	Cheng et al. 2015
ST2287	JRZH00000000	19	Human	1995	China	biphasic	Cheng et al. 2015
ST2533	JRZP00000000	19	Human	2006	China	biphasic	Cheng et al. 2015
ST2850	JRZV00000000	19	Human	2005	China	biphasic	Cheng et al. 2015
ST3363	JRZJ00000000	19	Human	1996	China	biphasic	Cheng et al. 2015
ST372	JRZT00000000	19	Human	2006	China	biphasic	Cheng et al. 2015
ST3858	JRZI00000000	19	Human	1996	China	biphasic	Cheng et al. 2015
ST4024	JRYU00000000	34	Human	2007	China	monophasic	Cheng et al. 2015
ST4038	JRZK00000000	19	Human	2002	China	monophasic	Cheng et al. 2015
ST4329	JRZL00000000	19	Human	2005	China	monophasic	Cheng et al. 2015
ST4650	JRZN00000000	19	Human	2006	China	biphasic	Cheng et al. 2015
ST4848	AUXE00000000	19	Human	2006	China	biphasic	Cheng et al. 2015
ST486	JRZQ00000000	19	Human	2006	China	monophasic	Cheng et al. 2015
ST6988	JRZW00000000	19	Human	1994	China	biphasic	Cheng et al. 2015
ST728	JRYT00000000	19	Human	2007	China	biphasic	Cheng et al. 2015
ST8493	JRZU00000000	19	Human	1993	China	biphasic	Cheng et al. 2015
DT120	ERS007566	34	Human	2009	UK	biphasic	Okoro et al. 2012
DT193	ERS007576	34	Human	2009	UK	biphasic	Okoro et al. 2012
H09164 0090	ERS007590	34	Human	2009	UK	biphasic	Okoro et al. 2012
DT97	ERS007594	34	Human	2009	UK	biphasic	Okoro et al. 2012
S15	JRGR00000000	34	Pig	2004	Denmark	monophasic	Qin et al. 2014
S23	JRGT00000000	34	Pig	2004	Denmark	monophasic	Qin et al. 2014
S7	JRGS00000000	34	Pig	2004	Denmark	monophasic	Qin et al. 2014
A130	ERS007468	313	Human	2001		biphasic	Petrovska et al. 2016

105841997	ERS008962	19	ND	1997		biphasic	Petrovska et al. 2016
S0337107	ERS008971	34	Pig	2007	UK	monophasic	Petrovska et al. 2016
S0292307	ERS008972	34	Cattle	2007	UK	monophasic	Petrovska et al. 2016
S0344705	ERS008963	34	Horse	2005	UK	monophasic	Petrovska et al. 2016
S0272405	ERS008964	19	Pig	2005		monophasic	Petrovska et al. 2016
S0565506	ERS008966	34	Cattle	2006	UK	monophasic	Petrovska et al. 2016
S0657807	ERS008967	34	Cat	2007	UK	monophasic	Petrovska et al. 2016
S0806007	ERS008968	34	Pig	2007	UK	monophasic	Petrovska et al. 2016
L0064707	ERS008969	34	Pig	2007	UK	monophasic	Petrovska et al. 2016
S0509207	ERS008970	34	Dog	2007	UK	monophasic	Petrovska et al. 2016
H09394 0492	ERS007564	19	Human	2009		biphasic	Petrovska et al. 2016
H09256 0454	ERS007582	19	Human	2009		biphasic	Petrovska et al. 2016
H09414 0613	ERS007567	19	Human	2009		biphasic	Petrovska et al. 2016
H08390 0191	ERS007572	19	Human	2008		biphasic	Petrovska et al. 2016
H09226 0446	ERS007574	19	Human	2009		biphasic	Petrovska et al. 2016
H09332 0603	ERS007578	19	Human	2009		biphasic	Petrovska et al. 2016
H09214 0797	ERS007588	19	Human	2009		biphasic	Petrovska et al. 2016
H09376 0485	ERS007606	19	Human	2009		biphasic	Petrovska et al. 2016
H09366 0457	ERS007608	19	Human	2009		biphasic	Petrovska et al. 2016
H09270 0335	ERS007592	19	Human	2009		biphasic	Petrovska et al. 2016
H09254 0380	ERS007596	19	Human	2009		biphasic	Petrovska et al. 2016
H09130 0134	ERS007598	19	Human	2009		biphasic	Petrovska et al. 2016
H09024 0100	ERS007600	19	Human	2009		biphasic	Petrovska et al. 2016
H09282 0253	ERS007602	568	Human	2009		biphasic	Petrovska et al. 2016
H09152 0230	ERS007604	19	Human	2009		biphasic	Petrovska et al. 2016
S0344408	ERS008974	34	Sheep	2008	UK	monophasic	Petrovska et al. 2016
L0085709	ERS008976	34	Dog	2009	UK	monophasic	Petrovska et al. 2016
S0354909	ERS008979	34	Cattle	2009	UK	monophasic	Petrovska et al. 2016
L0004109	ERS008980	34	Dog	2009	UK	monophasic	Petrovska et al. 2016
S0387409	ERS008981	34	Cattle	2009	UK	monophasic	Petrovska et al. 2016
S0433209	ERS008982	34	Pig	2009	UK	monophasic	Petrovska et al. 2016

S04696-09	ERS015598	19	Cattle	2009	biphasic	Petrovska et al. 2016
10084-1995	ERS015599	376	Cattle	1995	biphasic	Petrovska et al. 2016
100419-1995	ERS015600	376	ND	1995	biphasic	Petrovska et al. 2016
S00454-09	ERS015601	19	Horse	2009	biphasic	Petrovska et al. 2016
547-2001	ERS015613	19	Pig	2001	biphasic	Petrovska et al. 2016
10902-1996	ERS015614	19	Env	1996	biphasic	Petrovska et al. 2016
7828-1995	ERS015605	19	Pig	1995	biphasic	Petrovska et al. 2016
7830-1995	ERS015606	19	ND	1995	biphasic	Petrovska et al. 2016
7302-1999	ERS015607	19	Pig	1999	biphasic	Petrovska et al. 2016
S00914-05	ERS015608	19	Cattle	2005	biphasic	Petrovska et al. 2016
4582-1995	ERS015609	19	Cattle	1995	biphasic	Petrovska et al. 2016
12005-1995	ERS015610	19	Pig	1995	biphasic	Petrovska et al. 2016
3203-1997	ERS015611	19	Pig	1997	biphasic	Petrovska et al. 2016
10984-1996	ERS015612	19	Cattle	1996	biphasic	Petrovska et al. 2016
1713-1998	ERS015615	19	Cattle	1998	biphasic	Petrovska et al. 2016
1164-1998	ERS015624	568	Cattle	1998	biphasic	Petrovska et al. 2016
818-1998	ERS015625	568	Chicken	2008	biphasic	Petrovska et al. 2016
S083001-02	ERS015618	19	Pig	2002	biphasic	Petrovska et al. 2016
4284-1995	ERS015619	19	Cattle	1995	biphasic	Petrovska et al. 2016
4179-2001	ERS015620	19	Bird	2001	biphasic	Petrovska et al. 2016
8935-1997	ERS015621	19	Sheep	1997	biphasic	Petrovska et al. 2016
6940-1998	ERS015622	19	Dog	1998	biphasic	Petrovska et al. 2016
S06221-07	ERS015623	34	Cattle	2007	biphasic	Petrovska et al. 2016
3299-1997	ERS015626	2866	Bird	1997	biphasic	Petrovska et al. 2016
5544-1997	ERS015635	19	Env	1997	biphasic	Petrovska et al. 2016
11020-1996	ERS015636	19	Pig	1996	biphasic	Petrovska et al. 2016
2610-1998	ERS015627	19	Horse	1998	biphasic	Petrovska et al. 2016
6353-1997	ERS015628	19	Pig	1997	biphasic	Petrovska et al. 2016
8721-1997	ERS015629	19	Env	1997	biphasic	Petrovska et al. 2016
1402-2000	ERS015631	19	Cattle	2000	biphasic	Petrovska et al. 2016
SO3185-03	ERS015632	19	Cattle	2003	biphasic	Petrovska et al. 2016

SO9207-07	ERS015633	19	Pig	2007	biphasic	Petrovska et al. 2016
8380-1996	ERS015634	19	Pig	1996	biphasic	Petrovska et al. 2016
9115-1996	ERS015637	19	Cattle	1996	biphasic	Petrovska et al. 2016
SO4744-08	ERS015646	19	Chicken	2008	biphasic	Petrovska et al. 2016
7396-1998	ERS015647	98	Parrot	1998	biphasic	Petrovska et al. 2016
388-1998	ERS015638	19	Dog	1998	biphasic	Petrovska et al. 2016
SO8313-02	ERS015639	19	Pig	2002	biphasic	Petrovska et al. 2016
SO4454-08	ERS015640	19	Pig	2008	biphasic	Petrovska et al. 2016
SO6356-04	ERS015641	2864	Food	2004	biphasic	Petrovska et al. 2016
SO1491-06	ERS015642	19	Pigeon	2006	biphasic	Petrovska et al. 2016
SO6281-04	ERS015643	19	Duck	2004	biphasic	Petrovska et al. 2016
SO4178-09	ERS015644	19	Duck	2009	biphasic	Petrovska et al. 2016
8767-1998	ERS015645	19	Pig	1998	biphasic	Petrovska et al. 2016
SO3433-05	ERS015648	19	Chicken	2005	biphasic	Petrovska et al. 2016
SO5416-06	ERS015657	34	Chicken	2006	biphasic	Petrovska et al. 2016
SO5081-04	ERS015649	19	Horse	2004	biphasic	Petrovska et al. 2016
S00060-07	ERS015650	19	Horse	2007	biphasic	Petrovska et al. 2016
SO9304-02	ERS015651	19	Cattle	2002	biphasic	Petrovska et al. 2016
6164-1997	ERS015652	19	Chicken	1997	biphasic	Petrovska et al. 2016
S09313-03	ERS015653	19	Cattle	2003	biphasic	Petrovska et al. 2016
2087-1997	ERS015654	568	Finch	1997	biphasic	Petrovska et al. 2016
S07676-03	ERS015655	568	Bird	2003	biphasic	Petrovska et al. 2016
S05451-08	ERS015656	568	Dog	2008	biphasic	Petrovska et al. 2016
10258-1997	ERS015659	19	Turkey	1997	biphasic	Petrovska et al. 2016
SR11	ERS015668	19	ND	ND	biphasic	Petrovska et al. 2016
12342-1996	ERS015661	19	Duck	1996	biphasic	Petrovska et al. 2016
4300-2001	ERS015662	19	Duck	2001	biphasic	Petrovska et al. 2016
SO1960-05	ERS015663	19	Pig	2005	biphasic	Petrovska et al. 2016
S04199-08	ERS015664	19	Pig	2008	biphasic	Petrovska et al. 2016
S05968-02	ERS015665	19	Pig	2002	biphasic	Petrovska et al. 2016
S07292-07	ERS015666	19	Pig	2007	biphasic	Petrovska et al. 2016

S04782-03	ERS015667	19	Pig	2003		biphasic	Petrovska et al. 2016
10177-1993	ERS023488	19	Pig	1993		biphasic	Petrovska et al. 2016
L01001-10	ERS023497	34	Chicken	2010	UK	monophasic	Petrovska et al. 2016
S01569-10	ERS023498	34	Pig	2010	UK	monophasic	Petrovska et al. 2016
S04797-08	ERS023499	34	Bird	2008	UK	monophasic	Petrovska et al. 2016
10246-1993	ERS023489	19	Cattle	1993		biphasic	Petrovska et al. 2016
10382-1995	ERS023490	19	Cattle	1995		biphasic	Petrovska et al. 2016
1013-1997	ERS023491	19	Pig	1995		biphasic	Petrovska et al. 2016
11671-1996	ERS023492	19	Cattle	1996		biphasic	Petrovska et al. 2016
4061-1997	ERS023493	19	Pig	1997		biphasic	Petrovska et al. 2016
S03512-08	ERS023494	19	Cat	2008		monophasic	Petrovska et al. 2016
S00130-09	ERS023495	34	Cat	2009	UK	monophasic	Petrovska et al. 2016
S02412-09	ERS023496	19	Cattle	2009		monophasic	Petrovska et al. 2016
1334-1997	ERS023500	34	Pig	1997	UK	monophasic	Petrovska et al. 2016
L00938-09	ERS023509	34	Pig	2009	UK	monophasic	Petrovska et al. 2016
L00446-08	ERS023510	19	Chicken	2008		monophasic	Petrovska et al. 2016
L00178-09	ERS023511	19	Chicken	2009		monophasic	Petrovska et al. 2016
1731-1999	ERS023502	19	Pig	1999		monophasic	Petrovska et al. 2016
6887-2000	ERS023504	19	Pig	2000		monophasic	Petrovska et al. 2016
2798-2001	ERS023505	19	Pig	2001		monophasic	Petrovska et al. 2016
3543-2002	ERS023506	19	Pig	2002		monophasic	Petrovska et al. 2016
L01176-08	ERS023507	34	Pig	2008	UK	monophasic	Petrovska et al. 2016
L01189-08	ERS023508	34	Pig	2008	UK	monophasic	Petrovska et al. 2016
S00814-10	ERS023512	34	Chicken	2010	UK	monophasic	Petrovska et al. 2016
L1101-10	ERS023521	34	Chicken	2010	UK	monophasic	Petrovska et al. 2016
S4812-10	ERS023522	34	Pig	2010	UK	biphasic	Petrovska et al. 2016
S4489-10	ERS023523	19	ND	2010		biphasic	Petrovska et al. 2016
S03445-08	ERS023513	34	Sheep	2008	UK	monophasic	Petrovska et al. 2016
S07300-05	ERS023514	34	Cattle	2005	UK	monophasic	Petrovska et al. 2016
S00065-06	ERS023515	34	Cattle	2006	UK	monophasic	Petrovska et al. 2016
S01364-10	ERS023516	34	Cattle	2010	UK	monophasic	Petrovska et al. 2016

4824-10	ERS023517	34	Pig	2010	UK	monophasic	Petrovska et al. 2016
4797-10	ERS023518	34	Chicken	2010	UK	monophasic	Petrovska et al. 2016
S5712-08	ERS023519	19	Duck	2008		biphasic	Petrovska et al. 2016
S5828-08	ERS023520	19	Chicken	2008		biphasic	Petrovska et al. 2016
S3659-10	ERS023524	34	Chicken	2010	UK	monophasic	Petrovska et al. 2016
S03113-10	ERS023533	34	Pig	2010	UK	monophasic	Petrovska et al. 2016
S04698-09	ERS023534	34	Cattle	2009	UK	monophasic	Petrovska et al. 2016
L00759-09	ERS023535	19	Snake	2009		monophasic	Petrovska et al. 2016
S00250-07	ERS023525	19	Pig	2007		monophasic	Petrovska et al. 2016
S00176-09	ERS023526	34	Pig	2009	UK	monophasic	Petrovska et al. 2016
L00961-04	ERS023527	19	Pig	2004		monophasic	Petrovska et al. 2016
5102-1999	ERS023528	19	Pig	1999		monophasic	Petrovska et al. 2016
L01730-06	ERS023529	34	Pig	2006	UK	monophasic	Petrovska et al. 2016
S02909-08	ERS023530	34	Pig	2008	UK	monophasic	Petrovska et al. 2016
S05893-09	ERS023531	34	Pig	2009	UK	monophasic	Petrovska et al. 2016
S05894-09	ERS023532	34	Pig	2009	UK	monophasic	Petrovska et al. 2016
H103260370	ERS037925	34	Human	2010	UK	monophasic	Petrovska et al. 2016
H105100366	ERS037934	19	Human	2010		biphasic	Petrovska et al. 2016
H105260826	ERS037935	34	Human	2010	UK	biphasic	Petrovska et al. 2016
H105280433	ERS037936	34	Human	2010	UK	monophasic	Petrovska et al. 2016
H103700509	ERS037926	19	Human	2010		biphasic	Petrovska et al. 2016
H103720606	ERS037927	19	Human	2010		monophasic	Petrovska et al. 2016
H103920583	ERS037928	34	Human	2010	UK	monophasic	Petrovska et al. 2016
H1041406001	ERS037929	34	Human	2010	UK	monophasic	Petrovska et al. 2016
H104240404	ERS037930	34	Human	2010	UK	monophasic	Petrovska et al. 2016
H104680513	ERS037931	34	Human	2010	UK	biphasic	Petrovska et al. 2016
H105000301	ERS037933	34	Human	2010	UK	monophasic	Petrovska et al. 2016
45/16	ERS037951	34	Pig	2006	Italy	monophasic	Petrovska et al. 2016
2200/2	ERS037960	34	Pig	2009	Italy	monophasic	Petrovska et al. 2016
2448/2	ERS037961	34	Cattle	2010	Italy	monophasic	Petrovska et al. 2016
1038/2	ERS037962	19	ND	ND		monophasic	Petrovska et al. 2016

496/10	ERS037963	34	Cattle	2006	Italy	monophasic	Petrovska et al. 2016
1115/25	ERS037964	19	Cattle	ND		monophasic	Petrovska et al. 2016
1686/1	ERS037968	34	Human	2007	Italy	monophasic	Petrovska et al. 2016
1790/1	ERS037969	34	Turkey	2007	Italy	monophasic	Petrovska et al. 2016
242/2	ERS037952	34	Shellfish	2006	Italy	monophasic	Petrovska et al. 2016
2617/20	ERS037970	34	Pig	2010	Italy	monophasic	Petrovska et al. 2016
1948/2	ERS037971	34	Pig	2007	Italy	monophasic	Petrovska et al. 2016
1693/1	ERS043715	34	Human	2007	Italy	monophasic	Petrovska et al. 2016
3046/11	ERS037953	34	Cattle	2008	Italy	monophasic	Petrovska et al. 2016
1365/1	ERS037954	34	ND	2009	Italy	monophasic	Petrovska et al. 2016
2841/2	ERS037955	34	Human	2009	Italy	monophasic	Petrovska et al. 2016
692/26	ERS037956	34	Pig	2008	Italy	monophasic	Petrovska et al. 2016
2117/2	ERS037957	34	Shellfish	2008	Italy	monophasic	Petrovska et al. 2016
629/2	ERS037958	34	Pig	2008	Italy	monophasic	Petrovska et al. 2016
2223/2	ERS037959	34	Pig	2010	Italy	monophasic	Petrovska et al. 2016
H07 016 0417	ERS037901	19	Human	2007		monophasic	Petrovska et al. 2016
H07 246 0339	ERS037910	34	Human	2007	UK	monophasic	Petrovska et al. 2016
H07 362 0321	ERS037911	34	Human	2007	UK	monophasic	Petrovska et al. 2016
H090260055	ERS037913	19	Human	2009		monophasic	Petrovska et al. 2016
H100120548	ERS037914	34	Human	2010	UK	monophasic	Petrovska et al. 2016
H100420171	ERS037915	19	Human	2010		monophasic	Petrovska et al. 2016
H100760028	ERS037916	19	Human	2010		monophasic	Petrovska et al. 2016
H100800267	ERS037917	34	Human	2010	UK	monophasic	Petrovska et al. 2016
H101020440	ERS037918	19	Human	2010		monophasic	Petrovska et al. 2016
H101560198	ERS037919	99	Human	2010		monophasic	Petrovska et al. 2016
H07 166 0082	ERS037902	34	Human	2007	UK	monophasic	Petrovska et al. 2016
H102120667	ERS037921	34	Human	2010	UK	monophasic	Petrovska et al. 2016
H10234093302	ERS037923	34	Human	2010	UK	biphasic	Petrovska et al. 2016
H07 182 0182	ERS037903	34	Human	2007	UK	monophasic	Petrovska et al. 2016
H07 230 0280	ERS037904	34	Human	2007	UK	monophasic	Petrovska et al. 2016
H07 234 0179	ERS037905	19	Human	2007		monophasic	Petrovska et al. 2016

H07 246 0338	ERS037906	34	Human	2007	UK	monophasic	Petrovska et al. 2016
H07 276 0382	ERS037907	34	Human	2007	UK	monophasic	Petrovska et al. 2016
H07 338 0264	ERS037908	34	Human	2007	UK	monophasic	Petrovska et al. 2016
H07 394 0379	ERS037909	34	Human	2007	UK	monophasic	Petrovska et al. 2016
SL1344	FQ312003/HE654724/ HE654725/HE654726	19	Cattle			biphasic	Hoiseth & Stocker 1981, Kroger et al. 2012
20160374	ERS1545097	34	Human	2016	UK	monophasic	This study
20160407	ERS1545098	34	Human	2016	UK	monophasic	This study
