# Supplementary material for: New Variant of Multidrug-Resistant Salmonella enterica Serovar Typhimurium Associated with Invasive Disease in Immunocompromised Patients in Vietnam
Source: mBio. 2018 Sep 4;9(5):e01056-18. doi: 10.1128/mBio.01056-18 (PMC6123440; doi:10.1128/mBio.01056-18)
Supplement: TABLE S3 [file mbo004184053st3.pdf]

Table S3. The numbers of isolates from humans and animals (combined animal species) in Vietnam per BAPS clade.

	BAPS clade				
	1	2	3	4	5
Animal	57	26	3	14	13
Human	2	45	12	23	3
