# Supplementary material for: New Variant of Multidrug-Resistant Salmonella enterica Serovar Typhimurium Associated with Invasive Disease in Immunocompromised Patients in Vietnam
Source: mBio. 2018 Sep 4;9(5):e01056-18. doi: 10.1128/mBio.01056-18 (PMC6123440; doi:10.1128/mBio.01056-18)
Supplement: FIG S2 [file mbo004184053sf2.pdf]

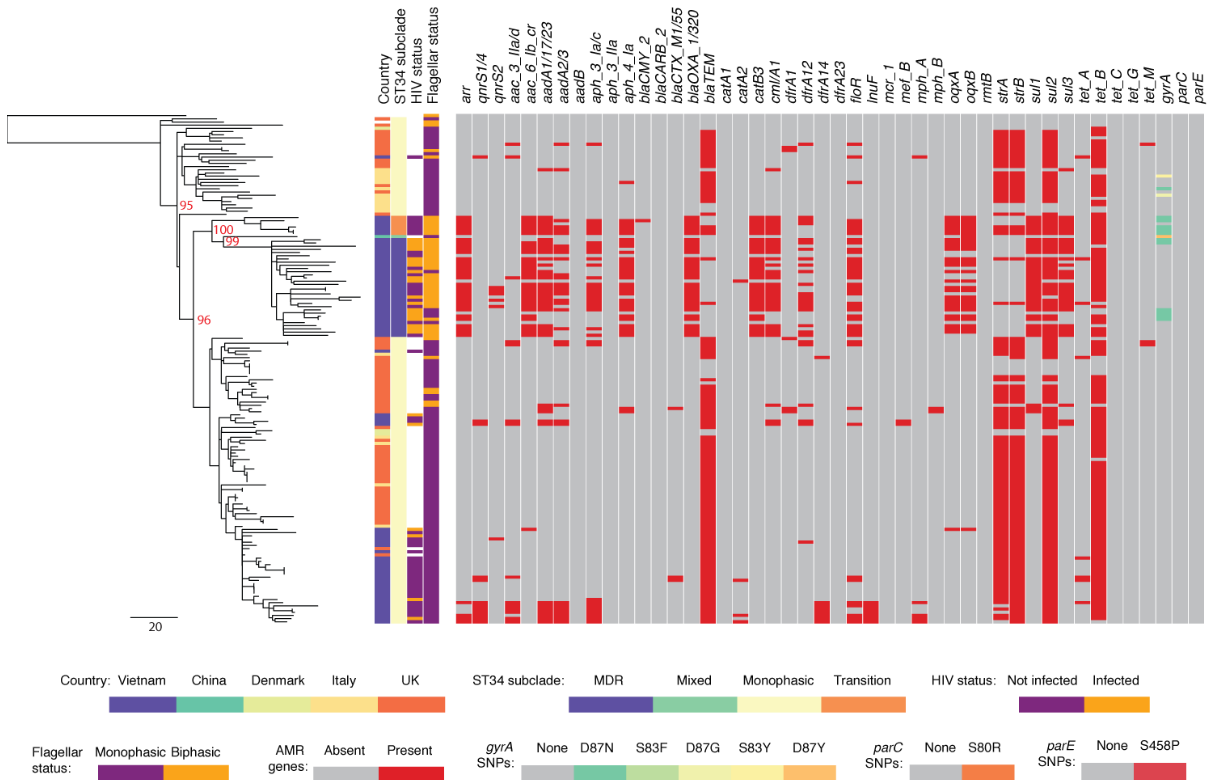

Fig. S2. Subset of the maximum likelihood phylogeny from Fig. 2, showing the ST34 *S.* Typhimurium/*S.* I:4,[5],12:i:- isolates from the Vietnam and context collections, mapped to monophasic reference SO4698-09. Key bootstrap values relating to the ST34 subclades are indicated in red on the relevant nodes. Scale bar represents the number of non-recombinogenic single nucleotide polymorphisms per branch.
