# Supplementary material for: New Variant of Multidrug-Resistant Salmonella enterica Serovar Typhimurium Associated with Invasive Disease in Immunocompromised Patients in Vietnam
Source: mBio. 2018 Sep 4;9(5):e01056-18. doi: 10.1128/mBio.01056-18 (PMC6123440; doi:10.1128/mBio.01056-18)
Supplement: TABLE S4 [file mbo004184053st4.pdf]

Table S4. Phenotypic antimicrobial resistance profiles for the 198 *S.* Typhimurium/*S.* I:4,[5],12:i:- isolates from Vietnam. Antimicrobial drugs to which isolates are resistant are listed; drugs to which isolates are have intermediate resistance are listed in brackets. Ap=ampicillin; Amc=amoxicillin/clavulanate; Cz=ceftazidime; Cx=ceftriaxone; Ch=chloramphenicol; Cp=ciprofloxacin; Gm=gentamicin; Nal=nalidixic acid; Of=ofloxacin; Tm=trimethoprim.

Sample	Phenotype	Sample	Phenotype
73_V_020		VNB589	ApAmcCh(Cp)GmNalTm
73_V_253		73_H_001	ApCh(Nal)Tm
73_V_282		73_H_110	ApChTm
73_V_286	(Tm)	73_H_111	ApChTm
73_V_283		73_H_112	ApChTm
73_V_285		73_H_129	Ap
73_V_320		73_V_110	
73_V_321		73_H_170	ApChTm
73_V_322		73_H_172	ApAmcCzCxCh(Nal)
73_V_336		73_H_243	ApChGmNal(Of)Tm
73_V_335		74_H_047	ApChTm
73_V_023		74_H_072	ApChGmNalOfTm
73_V_364	ApChTm	74_H_125	Ap(Amc)Ch(Gm)NalTm
73_V_381		74_H_126	Ap(Amc)Ch(Gm)Nal(Tm)
73_V_425	(Tm)	74_H_253	ApGm(Nal)Tm
73_V_423		74_H_256	ApGmNal(Of)Tm
73_V_426	(Tm)	VNB1779	ApChGmNal
74_V_100		73_V_113	
74_V_101		VNB1792	ApCh(Cp)GmNalTm
74_V_102		VNB1870	Gm
74_V_129		VNB2140	Ap(Amc)ChGmNalTm
74_V_202		VNB2175	Ap(Amc)Ch(Nal)Tm
73_V_039		VNB2200	

74_V_217		VNB2315	Ap(Ch)Nal
74_V_235	ApChNal(Of)Tm	VNB2339	
74_V_310		VNB2605	Ap(Amc)ChGmNal(Of)Tm
74_V_368		73_V_114	(Tm)
74_V_419		Hue_11	ApChGmTm
74_V_418		Hue_59	Tm
71_G_169	ApChTm	Hue_98	
71_G_450	ApAmcCzCxChGm(Nal)	KH_69	ApAmcCh(Cp)Tm
72-G-120		73_V_168	
73_G_051	Ap	74_G_043	
73_V_038		74_G_339	ApCh(Nal)Tm
73_G_047	Ap	71_V_080	
73_G_049	Ap	71_V_097	ApTm
73_G_050	Ap	71_V_115	
71_H_035	ApAmcChCpGmNal(Of)	71_V_330	ApChGm(Nal)Tm
71_H_034	Ap(Amc)ChCpGmNal(Of)(Tm)	71_V_389	
71_H_053	Ap	71_V_387	
71_H_052	Ap	71_V_385	
71_H_051	Ap	71_V_386	
71_H_085	ApChTm	71_V_466	ApTm
71_H_083		71_V_465	
73_V_065		71_V_480	
71_H_084	ChTm	71_V_479	(Amc)
71_H_243	ApCh(Nal)Tm	71_V_478	
72_H_033	Ap(Amc)Ch(Cp)Gm(Nal)Tm	72_V_054	
72_H_265	ApCh(Cp)GmNal(Of)Tm	72_V_055	
72_H_332	Ap(Ch)Nal	72_V_085	
72_V_083		72_V_082	
72_V_111		VNB773	ApCh(Cp)NalTm

72_V_228	
72_V_267	
72_V_269	
72_V_282	
73_V_001	ApTm
MT13C.2.2	Ap(Amc)Ch(Cp)GmTm
CT49_2	(Cp)
CT55_1	ApCh(Cp)Gm
CT69_2	Ap
71_H_455	ApCh(Cp)GmNalTm
71_H_114	
71_H_228	ApChTm
71_V_202	
71_V_204	
71_V_313	
72-G-232	ApCh(Nal)Tm
VNS10045	ApAmcCzCxChNalTm
VNDSal1	(Gm)
VNS10052	ApChGmNalTm
VNS20005	ApAmcCzCx
VNS20207	ApChTm
VNS30099	ApChTm
VNS121 DQT	NalTm
VNS10314	Ap
VNB455	Ap(Cp)
VNS10413	Ap(Amc)ChGm
VNB712	Ap(Amc)Ch(Cp)Gm
VNB1222	ApChTm
VNDSal2	

VNB1403	
VNDSal4	
VNB1701	ApCh(Cp)GmNal
VNS10137	ApNalTm
VNS30385	
VNS20057	ApTm
VNS20278	ApChGmNalTm
VNS30161	Ap
VNSC2045	Ap
VNB170	Ap(Cp)GmTm
VNB596	Ap(Amc)Ch(Cp)Nal
VNB802	Ap(Amc)Ch(Cp)Gm(Nal)Tm
VNB1428	ApCh(Cp)Tm
VNDSal5	
VNSC2362	
VNS10146	ApChCpGmNal(Of)Tm
VNDSal8	(Gm)
VNS20337	ApChTm
VNS30243	ApChTm
VNSC2047	Nal
VNB176	Ap(Amc)Ch(Cp)GmNalTm
VNB617	Ap(Amc)ChCpGmNal(Tm)
VNB845	Ch(Cp)Nal
VNB1436	Ap(Ch)(Cp)(Nal)
VNB198	ApCh(Cp)GmNal
VNDSal6	
VNS10182	ApAmcChGmNalTm
VNS20101	Ap
VNS20480	ApCh(Nal)Tm

VNS10068	ApChTm	VNS30267	Ap(Amc)ChGm
VNS20007	Ap	VNSC2191	Ap
VNS20235	Ap	VNB177	Ap(Amc)Ch(Cp)Gm
VNS165 VDQ	ApGmTm	VNB652	ApCh(Cp)GmNalTm
VNS20150		VNB692	Ap(Amc)Ch(Cp)NalTm
VNB148	Ap(Amc)Ch(Cp)Nal	VNB922	Ap(Amc)Ch(Cp)Gm(Nal)Tm
VNB541	Ap(Amc)Ch(Cp)Gm(Nal)Tm	VNB1479	ApCh(Cp)Gm(Nal)Tm
VNB745	(Cp)Nal	VNDSal7	(Gm)
VNB1264	ApChCpGmNal(Of)Tm	VNS20099	Ap
VNDSal3		VNS30012	
VNB68	Ap(Amc)(Cp)GmTm	VNS30356	Ap
VNS10124		VNSC2235	
VNS20018	ApChNalTm	VNB1166	Ap(Cp)
VNS20277	ApChTm	VNB184	Ap(Cp)GmTm
VNS30015	Ch	VNB664	ApCh(Cp)NalTm
VNS30144	ApAmcCh(Cp)GmNal	VNB1140	Ap(Amc)ChCpGmNalTm
VNSC2442	Nal	VNB1505	ApChGm(Nal)Tm
VNB151	ApAmcCxCh(Cp)GmNal(Tm)	VNS20081	ApAmcCz(Cx)Ch(Cp)GmNalTm
