# Supplementary material for: New Variant of Multidrug-Resistant Salmonella enterica Serovar Typhimurium Associated with Invasive Disease in Immunocompromised Patients in Vietnam
Source: mBio. 2018 Sep 4;9(5):e01056-18. doi: 10.1128/mBio.01056-18 (PMC6123440; doi:10.1128/mBio.01056-18)
Supplement: TABLE S5 [file mbo004184053st5.pdf]

Table S5. Acquired genes and single nucleotide polymorphisms conferring antimicrobial resistance, along with the relevant accession numbers, in the isolates from Vietnam and the context collection.

Isolate	Genotype
73_V_253	
73_V_282	
73_V_286	dfrA14.1_DQ388123
73_V_283	dfrA14.1_DQ388123
73_V_285	dfrA14.1_DQ388123
73_V_320	
73_V_321	
73_V_322	
73_V_336	
73_V_335	
73_V_020	
73_V_364	QnrS1.1_AB187515, aadA1.3_JQ414041, aadA2.1_X68227, blaTEM_1B.1_JF910132, cmlA1.1_M64556, dfrA12.1_AB571791, floR.2_AF118107, sul2.2_GQ421466, sul3.2_AJ459418, tet_A_.4_AJ517790, tet_M_.8_X04388
73_V_381	dfrA14.1_DQ388123
73_V_425	dfrA14.1_DQ388123
73_V_423	
73_V_426	dfrA14.1_DQ388123
74_V_100	
74_V_101	
74_V_102	
74_V_129	
74_V_202	
73_V_023	
74_V_217	

74_V_235	ARR_2.1_HQ141279, QnrS1.1_AB187515, aadA17.1_FJ460181, aadA2.1_X68227, aph_3___Ic.1_X62115, blaTEM_1B.1_JF910132, catA2.1_X53796, dfrA14.1_DQ388123, floR.2_AF118107, lnu_F_.1_EU118119, mph_A_.1_D16251, strA.4_AF321551, strB.1_M96392, sul2.2_GQ421466, tet_B_.4_AF326777
74_V_310	
74_V_368	
74_V_419	sul2.3_HQ840942
74_V_418	
71_G_169	QnrS1.1_AB187515, aadA1.3_JQ414041, aadA2.2_JQ364967, blaTEM_1B.1_JF910132, cmlA1.1_M64556, dfrA12.1_AB571791, floR.2_AF118107, mcr_1.1_KP347127, sul2.2_GQ421466, sul3.2_AJ459418, tet_A_.4_AJ517790, tet_M_.8_X04388
71_G_450	QnrS1.1_AB187515, aac_3__IId.1_EU022314, blaCTX_M_55.2_GQ456159, blaTEM_1B.1_JF910132, floR.2_AF118107, strA.4_AF321551, strB.1_M96392, sul2.3_HQ840942, tet_A_.4_AJ517790, tet_B_.4_AF326777
72-G-120	
73_G_051	blaTEM_1B.1_JF910132, strA.4_AF321551, strB.1_M96392, sul2.3_HQ840942, tet_B_.4_AF326777
73_V_039	
73_G_047	blaTEM_1B.1_JF910132, strA.4_AF321551, strB.1_M96392, sul2.3_HQ840942, tet_B_.4_AF326777
73_G_049	blaTEM_1B.1_JF910132, strA.4_AF321551, strB.1_M96392, sul2.3_HQ840942, tet_B_.4_AF326777
73_G_050	blaTEM_1B.1_JF910132, strA.4_AF321551, strB.1_M96392, sul2.3_HQ840942, tet_B_.4_AF326777
71_H_035	ARR_3.1_JF806499, QnrS2.1_JF261185, aac_6__Ib_cr.1_DQ303918, aadA1.3_JQ414041, aadA2.1_X68227, aph_3___Ia.1_V00359, aph_4__Ia.1_V01499, blaOXA_1.1_J02967, catB3.1_AJ009818, cmlA1.1_M64556, floR.2_AF118107, oqxA.1_EU370913, oqxB.1_EU370913, sul1.2_CP002151, sul2.2_GQ421466, sul3.2_AJ459418, tet_B_.4_AF326777
71_H_034	ARR_3.1_JF806499, QnrS2.1_JF261185, aac_6__Ib_cr.1_DQ303918_2, aadA1.3_JQ414041, aadA2.1_X68227, aph_3___Ia.1_V00359, aph_4__Ia.1_V01499, blaOXA_1.1_J02967, catB3.1_AJ009818, cmlA1.1_M64556, floR.2_AF118107, oqxA.1_EU370913, oqxB.1_EU370913, sul1.2_CP002151, sul2.2_GQ421466, sul3.2_AJ459418, tet_B_.4_AF326777
71_H_053	blaTEM_1B.1_JF910132, strA.4_AF321551, strB.1_M96392, sul2.3_HQ840942, tet_B_.4_AF326777
71_H_052	blaTEM_1B.1_JF910132, strA.4_AF321551, strB.1_M96392, sul2.3_HQ840942, tet_B_.4_AF326777
71_H_051	blaTEM_1B.1_JF910132, strA.4_AF321551, strB.1_M96392, sul2.3_HQ840942, tet_B_.4_AF326777
71_H_085	QnrS1.1_AB187515, aadA1.3_JQ414041, aadA2.1_X68227, blaTEM_1B.1_JF910132, cmlA1.1_M64556, dfrA12.1_AB571791, floR.2_AF118107, sul2.2_GQ421466, sul3.2_AJ459418, tet_A_.4_AJ517790, tet_M_.8_X04388
71_H_083	
73_V_038	
71_H_084	aadA2.1_X68227, cmlA1.1_M64556, dfrA12.1_AB571791, sul2.2_GQ421466, sul3.2_AJ459418, tet_A_.4_AJ517790, tet_M_.8_X04388
71_H_243	QnrS1.1_AB187515, aadA1.3_JQ414041, aadA2.1_X68227, blaTEM_1B.1_JF910132, cmlA1.1_M64556, dfrA12.1_AB571791, floR.2_AF118107, sul2.2_GQ421466, sul3.2_AJ459418, tet_A_.4_AJ517790, tet_M_.8_X04388

72_H_033	ARR_3.1_JF806499, QnrS2.1_JF261185, aac_6__Ib_cr.1_DQ303918, aadA1.3_JQ414041, aadA2.1_X68227, aph_3___Ia.1_V00359, aph_4__Ia.1_V01499, blaOXA_1.1_J02967, catB3.1_AJ009818, cmlA1.1_M64556, dfrA12.1_AB571791, floR.2_AF118107, sul1.2_CP002151, sul2.2_GQ421466, sul3.2_AJ459418, tet_B_.4_AF326777
72_H_265	ARR_2.1_HQ141279, QnrS1.1_AB187515, aac_3__IId.1_EU022314, aadA17.1_FJ460181, aadA2.1_X68227, aph_3___Ic.1_X62115, blaTEM_1B.1_JF910132, dfrA14.1_DQ388123, floR.2_AF118107, lnu_F_.1_EU118119, mph_A_.1_D16251, strA.4_AF321551, strB.1_M96392, sul2.3_HQ840942, tet_A_.4_AJ517790, tet_B_.4_AF326777
72_H_332	aac_3__IId.1_EU022314, blaTEM_1B.1_JF910132, gyrA.D87N
73_H_001	QnrS1.1_AB187515, aadA1.3_JQ414041, aadA2.1_X68227, blaTEM_1B.1_JF910132, cmlA1.1_M64556, dfrA12.1_AB571791, floR.2_AF118107, sul2.2_GQ421466, sul3.2_AJ459418, tet_A_.4_AJ517790, tet_M_.8_X04388
73_H_110	QnrS1.1_AB187515, aadA1.3_JQ414041, aadA2.1_X68227, blaTEM_1B.1_JF910132, cmlA1.1_M64556, dfrA12.1_AB571791, floR.2_AF118107, sul2.2_GQ421466, sul3.2_AJ459418, tet_A_.4_AJ517790, tet_M_.8_X04388
73_H_111	QnrS1.1_AB187515, aadA1.3_JQ414041, aadA2.1_X68227, blaTEM_1B.1_JF910132, cmlA1.1_M64556, dfrA12.1_AB571791, floR.2_AF118107, sul2.2_GQ421466, sul3.2_AJ459418, tet_A_.4_AJ517790, tet_M_.8_X04388
73_H_112	QnrS1.1_AB187515, aadA1.3_JQ414041, aadA2.1_X68227, blaTEM_1B.1_JF910132, cmlA1.1_M64556, dfrA12.1_AB571791, floR.2_AF118107, sul2.2_GQ421466, sul3.2_AJ459418, tet_A_.4_AJ517790, tet_M_.8_X04388
73_H_129	blaTEM_1B.1_JF910132, strA.4_AF321551, strB.1_M96392, sul2.3_HQ840942, tet_B_.4_AF326777
73_V_065	
73_H_170	QnrS1.1_AB187515, aadA1.3_JQ414041, aadA2.1_X68227, blaTEM_1B.1_JF910132, cmlA1.1_M64556, dfrA12.1_AB571791, floR.2_AF118107, sul2.2_GQ421466, sul3.2_AJ459418, tet_A_.4_AJ517790, tet_M_.8_X04388
73_H_172	QnrS1.1_AB187515, blaCTX_M_55.2_GQ456159, blaTEM_1B.1_JF910132, catA2.1_X53796, floR.2_AF118107, strA.4_AF321551, strB.1_M96392, sul2.2_GQ421466, tet_A_.4_AJ517790, tet_B_.4_AF326777
73_H_243	QnrS1.1_AB187515, aac_3__IId.1_EU022314, aadA17.1_FJ460181, aadA2.1_X68227, aph_3___Ic.1_X62115, blaTEM_1B.1_JF910132, dfrA14.1_DQ388123, floR.2_AF118107, lnu_F_.1_EU118119, strB.1_M96392, sul2.3_HQ840942, tet_B_.4_AF326777
74_H_047	QnrS1.1_AB187515, aadA1.3_JQ414041, aadA2.1_X68227, blaTEM_1B.1_JF910132, cmlA1.1_M64556, dfrA12.1_AB571791, floR.2_AF118107, sul2.2_GQ421466, sul3.2_AJ459418, tet_A_.4_AJ517790, tet_M_.8_X04388
74_H_072	ARR_2.1_HQ141279, QnrS1.1_AB187515, aac_3__IId.1_EU022314, aadA17.1_FJ460181, aadA2.1_X68227, aph_3___Ic.1_X62115, blaTEM_1B.1_JF910132, catA2.1_X53796, dfrA14.1_DQ388123, floR.2_AF118107, lnu_F_.1_EU118119, mph_A_.1_D16251, strB.1_M96392, sul2.2_GQ421466
74_H_125	ARR_3.1_JF806499, aac_6__Ib_cr.1_DQ303918_2, aadA1.3_JQ414041, aadA2.1_X68227, aph_3___Ia.1_V00359, aph_4__Ia.1_V01499, blaOXA_1.1_J02967, blaTEM_1B.1_JF910132, catB3.1_AJ009818, cmlA1.1_M64556, floR.2_AF118107, oqxA.1_EU370913, oqxB.1_EU370913, strA.4_AF321551, strB.1_M96392, sul1.2_CP002151, sul2.2_GQ421466, sul3.2_AJ459418, tet_B_.4_AF326777, gyrA.D87N
74_H_126	ARR_3.1_JF806499, aac_6__Ib_cr.1_DQ303918, aadA1.3_JQ414041, aadA2.1_X68227, aph_3___Ia.1_V00359, aph_4__Ia.1_V01499, blaOXA_1.1_J02967, blaTEM_1B.1_JF910132, catB3.1_AJ009818, cmlA1.1_M64556, floR.2_AF118107, oqxA.1_EU370913, oqxB.1_EU370913, strA.4_AF321551, strB.1_M96392, sul1.2_CP002151, sul2.3_HQ840942, sul3.2_AJ459418, tet_B_.4_AF326777, gyrA.D87N
74_H_253	QnrS1.1_AB187515, aac_3__IId.1_EU022314, aadA17.1_FJ460181, aadA2.1_X68227, aph_3___Ic.1_X62115, blaTEM_1B.1_JF910132, dfrA14.1_DQ388123, lnu_F_.1_EU118119, strB.1_M96392, sul2.3_HQ840942, tet_B_.4_AF326777
74_H_256	QnrS1.1_AB187515, aac_3__IId.1_EU022314, aadA17.1_FJ460181, aadA2.1_X68227, aph_3___Ic.1_X62115, blaTEM_1B.1_JF910132, dfrA14.1_DQ388123, lnu_F_.1_EU118119, strA.4_AF321551, strB.1_M96392, sul2.3_HQ840942, tet_B_.4_AF326777
VNB1779	ARR_3.1_JF806499, aac_6__Ib_cr.1_DQ303918_2, aph_4__Ia.1_V01499, blaOXA_1.1_J02967, catB3.1_AJ009818, floR.2_AF118107, oqxA.1_EU370913, oqxB.1_EU370913, sul1.1_AY224185, sul2.2_GQ421466, tet_B_.4_AF326777, gyrA.D87N
73_V_110	

VNB1792	ARR_3.1_JF806499, aac_6__Ib_cr.1_DQ303918, aadA1.3_JQ414041, aadA2.1_X68227, aph_3___Ia.1_V00359, aph_4__Ia.1_V01499, blaOXA_1.1_J02967, catB3.1_AJ009818, cmlA1.1_M64556, dfrA12.1_AB571791, floR.2_AF118107, oqxA.1_EU370913, oqxB.1_EU370913, sul1.1_AY224185, sul2.2_GQ421466, sul3.2_AJ459418, gyrA.D87N
VNB1870	sul2.2_GQ421466
VNB2140	ARR_3.1_JF806499, aac_6__Ib_cr.1_DQ303918, aadA1.3_JQ414041, aadA2.1_X68227, aph_3___Ia.1_V00359, aph_4__Ia.1_V01499, blaOXA_1.1_J02967, catB3.1_AJ009818, cmlA1.1_M64556, oqxA.1_EU370913, oqxB.1_EU370913, sul1.1_AY224185, sul2.2_GQ421466, sul3.2_AJ459418
VNB2175	QnrS1.1_AB187515, aadA1.3_JQ414041, aadA2.1_X68227, blaTEM_1B.1_JF910132, cmlA1.1_M64556, dfrA12.1_AB571791, floR.2_AF118107, sul2.2_GQ421466, sul3.2_AJ459418, tet_A_.4_AJ517790, tet_M_.8_X04388
VNB2200	
VNB2315	aph_3___Ic.1_X62115, blaTEM_1B.1_JF910132, strA.4_AF321551, strB.1_M96392, sul2.3_HQ840942, tet_B_.4_AF326777
VNB2339	
VNB2605	ARR_2.1_HQ141279, QnrS1.1_AB187515, aac_3__IId.1_EU022314, aadA17.1_FJ460181, aadA2.1_X68227, aph_3___Ic.1_X62115, blaTEM_1B.1_JF910132, dfrA14.1_DQ388123, floR.2_AF118107, lnu_F_.1_EU118119, mph_A_.1_D16251, strA.4_AF321551, strB.1_M96392, sul2.3_HQ840942, tet_B_.4_AF326777
73_V_113	
Hue_11	ARR_3.1_JF806499, aac_6__Ib_cr.1_DQ303918_2, aadA1.3_JQ414041, aadA2.1_X68227, aph_3___Ia.1_V00359, aph_4__Ia.1_V01499, blaOXA_1.1_J02967, catB3.1_AJ009818, cmlA1.1_M64556, dfrA12.1_AB571791, floR.2_AF118107, sul1.2_CP002151, sul2.2_GQ421466, sul3.2_AJ459418, tet_B_.4_AF326777
Hue_59	aadA2.2_JQ364967, aph_3___Ia.1_V00359, dfrA12.1_AB571791, strA.4_AF321551, strB.1_M96392, sul2.3_HQ840942, tet_B_.4_AF326777
Hue_98	
KH_69	QnrS1.1_AB187515, aadA1.3_JQ414041, aadA2.1_X68227, blaTEM_1B.1_JF910132, cmlA1.1_M64556, dfrA12.1_AB571791, floR.2_AF118107, sul2.2_GQ421466, sul3.2_AJ459418, tet_A_.4_AJ517790, tet_M_.8_X04388
73_V_114	dfrA14.1_DQ388123
73_V_168	
74_G_043	
74_G_339	QnrS1.1_AB187515, aadA1.3_JQ414041, aadA2.1_X68227, blaTEM_1B.1_JF910132, cmlA1.1_M64556, dfrA12.1_AB571791, floR.2_AF118107, sul2.2_GQ421466, sul3.2_AJ459418, tet_A_.4_AJ517790, tet_M_.8_X04388
71_V_080	
71_V_097	blaTEM_1B.1_JF910132, dfrA14.1_DQ388123, strA.4_AF321551, strB.1_M96392, sul2.2_GQ421466
71_V_115	
71_V_330	ARR_2.1_HQ141279, QnrS1.1_AB187515, aac_3__IId.1_EU022314, aadA17.1_FJ460181, aadA2.1_X68227, blaTEM_1B.1_JF910132, catA2.1_X53796, dfrA14.1_DQ388123, lnu_F_.1_EU118119, mph_A_.1_D16251, strA.4_AF321551, strB.1_M96392, sul2.2_GQ421466, tet_A_.4_AJ517790
71_V_389	
71_V_387	

71_V_385	
71_V_386	
71_V_466	blaTEM_1B.1_JF910132, dfrA14.1_DQ388123, strA.4_AF321551, strB.1_M96392, sul2.2_GQ421466
71_V_465	
71_V_480	
71_V_479	
71_V_478	
72_V_054	
72_V_055	
72_V_085	
72_V_082	
72_V_083	
72_V_111	
72_V_228	
72_V_267	
72_V_269	
72_V_282	
73_V_001	blaTEM_1B.1_JF910132, dfrA14.1_DQ388123, strA.4_AF321551, strB.1_M96392, sul2.2_GQ421466
MT13C.2.2	QnrS1.1_AB187515, aac_3__IId.1_EU022314, aadA1.3_JQ414041, aadA2.1_X68227, blaTEM_1B.1_JF910132, cmlA1.1_M64556, dfrA12.1_AB571791, mef_B_.1_FJ196385, strA.4_AF321551, strB.1_M96392, sul2.2_GQ421466, sul3.2_AJ459418, tet_B_.4_AF326777
CT49_2	QnrS1.1_AB187515, aac_3__IId.1_EU022314, blaTEM_1B.1_JF910132, floR.2_AF118107, mph_A_.1_D16251, strA.4_AF321551, strB.1_M96392, sul2.3_HQ840942, tet_A_.4_AJ517790, tet_B_.4_AF326777
CT55_1	tet_B_.4_AF326777
CT69_2	blaTEM_1B.1_JF910132, strA.4_AF321551, strB.1_M96392, sul2.3_HQ840942, tet_B_.4_AF326777
71_H_455	ARR_3.1_JF806499, QnrS2.1_JF261185, aac_6__Ib_cr.1_DQ303918, aadA1.3_JQ414041, aph_3___Ia.1_V00359, aph_4__Ia.1_V01499, blaOXA_1.1_J02967, catB3.1_AJ009818, cmlA1.1_M64556, dfrA12.1_AB571791, floR.2_AF118107, oqxA.1_EU370913, oqxB.1_EU370913, sul1.1_AY224185, sul2.2_GQ421466, sul3.2_AJ459418, tet_B_.4_AF326777
71_H_114	
71_H_228	QnrS1.1_AB187515, aadA1.3_JQ414041, aadA2.1_X68227, blaTEM_1B.1_JF910132, cmlA1.1_M64556, dfrA12.1_AB571791, floR.2_AF118107, sul2.2_GQ421466, sul3.2_AJ459418, tet_A_.4_AJ517790, tet_M_.8_X04388
71_V_202	

71_V_204	
71_V_313	
72-G-232	QnrS1.1_AB187515, aadA1.3_JQ414041, aadA2.2_JQ364967, blaTEM_1B.1_JF910132, cmlA1.1_M64556, dfrA12.1_AB571791, floR.2_AF118107, sul2.2_GQ421466, sul3.2_AJ459418, tet_A_.4_AJ517790, tet_M_.8_X04388
20160374	aac_3__IIa.1_X51534, aadA2.2_JQ364967, aph_3___Ic.1_X62115, blaTEM_1B.1_JF910132, dfrA12.1_AB571791, floR.2_AF118107, strA.1_M96392, strB.1_M96392, sul2.3_HQ840942, tet_B_.4_AF326777, tet_M_.4_X75073
20160407	aac_3__IIa.1_X51534, aadA2.2_JQ364967, aph_3___Ic.1_X62115, blaTEM_1B.1_JF910132, dfrA12.1_AB571791, floR.2_AF118107, strA.1_M96392, strB.1_M96392, sul2.3_HQ840942, tet_B_.4_AF326777, tet_M_.4_X75073
A130	aadA1.2_JN815078, aadB.1_JN119852, blaOXA_1.1_J02967, blaTEM_1B.1_JF910132, dfrA23.1_AJ746361, sul1.1_AY224185
S0337107	blaTEM_1B.1_JF910132, strA.4_AF321551, strB.1_M96392, sul2.3_HQ840942, tet_B_.4_AF326777
S0292307	blaTEM_1B.1_JF910132, strA.4_AF321551, strB.1_M96392, sul2.3_HQ840942, tet_B_.4_AF326777
105841997	strA.4_AF321551, strB.1_M96392, sul2.2_GQ421466, tet_A_.4_AJ517790, gyrA.S83F
S0344705	blaTEM_1B.1_JF910132, strA.4_AF321551, strB.1_M96392, sul2.3_HQ840942, tet_B_.4_AF326777
S0272405	aadA2.2_JQ364967, blaTEM_1B.1_JF910132, cmlA1.1_M64556, dfrA12.1_AB571791, strA.4_AF321551, strB.1_M96392, sul2.2_GQ421466, sul3.2_AJ459418, tet_A_.4_AJ517790
S0565506	blaTEM_1B.1_JF910132, strA.4_AF321551, strB.1_M96392, sul2.3_HQ840942, tet_B_.4_AF326777
S0657807	blaTEM_1B.1_JF910132, strA.4_AF321551, strB.1_M96392, sul2.3_HQ840942, tet_B_.4_AF326777
S0806007	blaTEM_1B.1_JF910132, strA.4_AF321551, strB.1_M96392, sul2.3_HQ840942, tet_B_.4_AF326777
L0064707	blaTEM_1B.1_JF910132, strA.4_AF321551, strB.1_M96392, sul2.3_HQ840942, tet_B_.4_AF326777
S0509207	blaTEM_1B.1_JF910132, strA.4_AF321551, strB.1_M96392, sul2.3_HQ840942, tet_B_.4_AF326777
H09256 0454	
H09394 0492	
DT120	blaTEM_1B.1_JF910132, strA.4_AF321551, strB.1_M96392, sul2.3_HQ840942, tet_B_.4_AF326777
H09414 0613	
H08390 0191	
H09226 0446	tet_B_.4_AF326777
DT193	blaTEM_1B.1_JF910132, strA.4_AF321551, strB.1_M96392, sul2.3_HQ840942, tet_B_.4_AF326777
H09332 0603	
H09376 0485	aadA2.1_X68227, blaCARB_2.1_M69058, floR.2_AF118107, sul1.2_CP002151, tet_G_.5_AF071555
H09366 0457	tet_C_.5_NC_003213
H09214 0797	

H09164 0090	
H09270 0335	
DT97	blaTEM_1B.1_JF910132, dfrA1.30_JQ690541, strA.4_AF321551, strB.1_M96392, sul2.2_GQ421466, tet_B_.4_AF326777
H09254 0380	
H09130 0134	
H09024 0100	
H09282 0253	
H09152 0230	aadA1.3_JQ414041, aadA2.2_JQ364967, blaTEM_1B.1_JF910132, cmlA1.1_M64556, dfrA12.1_AB571791, strA.4_AF321551, strB.1_M96392, sul2.2_GQ421466, sul3.2_AJ459418, tet_A_.4_AJ517790
S0344408	blaTEM_1B.1_JF910132, strA.4_AF321551, strB.1_M96392, sul2.3_HQ840942, tet_B_.4_AF326777
L0085709	blaTEM_1B.1_JF910132, strA.4_AF321551, strB.1_M96392, sul2.3_HQ840942, tet_B_.4_AF326777
S0354909	blaTEM_1B.1_JF910132, strA.4_AF321551, strB.1_M96392, sul2.3_HQ840942, tet_B_.4_AF326777
L0004109	blaTEM_1B.1_JF910132, strA.4_AF321551, strB.1_M96392, sul2.3_HQ840942, tet_B_.4_AF326777
S0387409	blaTEM_1B.1_JF910132, strA.4_AF321551, strB.1_M96392, sul2.3_HQ840942, tet_B_.4_AF326777
S0433209	aadA1.5_JX185132, aph_4__Ia.1_V01499, blaCTX_M_1.6_DQ915955, blaTEM_1B.1_JF910132, dfrA1.30_JQ690541, floR.2_AF118107, mph_B_.1_D85892, strA.4_AF321551, strB.1_M96392, sul1.2_CP002151, sul2.2_GQ421466, tet_B_.4_AF326777
S04696-09	
10084-1995	
100419-1995	
S00454-09	
547-2001	
10902-1996	
7828-1995	dfrA14.1_DQ388123, strB.1_M96392, sul2.2_GQ421466
7830-1995	dfrA14.1_DQ388123, strB.1_M96392, sul2.2_GQ421466
7302-1999	
S00914-05	aadA2.1_X68227, blaCARB_2.1_M69058, floR.2_AF118107, tet_G_.4_AF133140
4582-1995	aadA2.1_X68227, sul1.1_AY224185
12005-1995	strA.4_AF321551, strB.1_M96392, sul2.2_GQ421466, tet_A_.4_AJ517790, gyrA.S83F
3203-1997	strA.4_AF321551, strB.1_M96392, sul2.2_GQ421466, tet_A_.4_AJ517790

10984-1996	
1164-1998	
818-1998	
1713-1998	blaTEM_1B.1_JF910132, tet_A_.4_AJ517790
S083001-02	aadA2.1_X68227, blaCARB_2.1_M69058, dfrA14.1_DQ388123, floR.2_AF118107, sul1.2_CP002151, sul2.2_GQ421466, tet_G_.5_AF071555
4284-1995	
4179-2001	
8935-1997	
6940-1998	
S06221-07	
5544-1997	blaTEM_1B.1_JF910132, strA.4_AF321551, strB.1_M96392, sul2.3_HQ840942, tet_A_.4_AJ517790, gyrA.S83F
11020-1996	gyrA.S83F
3299-1997	
2610-1998	
6353-1997	
8721-1997	dfrA14.1_DQ388123, strB.1_M96392, sul2.2_GQ421466, tet_B_.4_AF326777
1402-2000	
SO3185-03	
SO9207-07	
8380-1996	blaTEM_1B.1_JF910132, strA.4_AF321551, strB.1_M96392, sul2.3_HQ840942, tet_A_.4_AJ517790, gyrA.S83F
SO4744-08	
7396-1998	
9115-1996	sul2.2_GQ421466
388-1998	dfrA14.1_DQ388123, strB.1_M96392, sul2.2_GQ421466, tet_B_.4_AF326777
SO8313-02	
SO4454-08	tet_B_.4_AF326777
SO6356-04	
SO1491-06	

SO6281-04	
SO4178-09	
8767-1998	dfrA14.1_DQ388123, strB.1_M96392, sul2.2_GQ421466, tet_B_.4_AF326777
SO5416-06	
SO3433-05	
SO5081-04	
S00060-07	
SO9304-02	
6164-1997	sul2.2_GQ421466, tet_C_.9_AY046276
S09313-03	
2087-1997	
S07676-03	
S05451-08	
SR11	
10258-1997	
12342-1996	
4300-2001	
SO1960-05	blaTEM_1B.1_JF910132, dfrA12.1_AB571791, strA.4_AF321551, strB.1_M96392, sul2.2_GQ421466, sul3.2_AJ459418, tet_A_.4_AJ517790
S04199-08	aadA2.1_X68227, blaCARB_2.1_M69058, floR.2_AF118107, tet_G_.4_AF133140
S05968-02	aph_3___Ic.1_X62115, strA.4_AF321551, strB.1_M96392, sul2.2_GQ421466, tet_A_.4_AJ517790, gyrA.S83F
S07292-07	strA.4_AF321551, strB.1_M96392, sul2.2_GQ421466, tet_A_.4_AJ517790
S04782-03	tet_C_.5_NC_003213
L01001-10	blaTEM_1B.1_JF910132, strA.4_AF321551, strB.1_M96392, sul2.3_HQ840942, tet_B_.4_AF326777
S01569-10	
S04797-08	blaTEM_1B.1_JF910132, strA.4_AF321551, strB.1_M96392, sul2.3_HQ840942, tet_B_.4_AF326777
10177-1993	blaTEM_1B.1_JF910132, strA.4_AF321551, strB.1_M96392, sul2.3_HQ840942, tet_A_.4_AJ517790
10246-1993	tet_A_.4_AJ517790
10382-1995	

1013-1997	blaTEM_1B.1_JF910132, strA.4_AF321551, strB.1_M96392, sul2.3_HQ840942, tet_A_.4_AJ517790
11671-1996	blaTEM_1B.1_JF910132, strA.4_AF321551, strB.1_M96392, sul2.3_HQ840942, tet_A_.4_AJ517790
4061-1997	blaTEM_1B.1_JF910132, strA.4_AF321551, strB.1_M96392, sul2.3_HQ840942, tet_A_.4_AJ517790
S03512-08	aadA1.2_JN815078, aadA2.2_JQ364967, blaTEM_1B.1_JF910132, cmlA1.1_M64556, dfrA12.1_AB571791, strA.4_AF321551, strB.1_M96392, sul2.2_GQ421466, sul3.2_AJ459418, tet_A_.4_AJ517790
S00130-09	blaTEM_1B.1_JF910132, strA.4_AF321551, strB.1_M96392, sul2.3_HQ840942, tet_B_.4_AF326777
S02412-09	
L00938-09	blaTEM_1B.1_JF910132, dfrA1.30_JQ690541, strA.4_AF321551, strB.1_M96392, sul2.2_GQ421466, tet_B_.4_AF326777
L00446-08	
L00178-09	
1334-1997	blaTEM_1B.1_JF910132, strA.4_AF321551, strB.1_M96392, sul2.3_HQ840942, tet_B_.4_AF326777
1731-1999	
6887-2000	tet_B_.4_AF326777
2798-2001	tet_B_.4_AF326777
3543-2002	tet_B_.4_AF326777
L01176-08	blaTEM_1B.1_JF910132, strA.4_AF321551, strB.1_M96392, sul2.3_HQ840942, tet_B_.4_AF326777
L01189-08	tet_B_.4_AF326777
L1101-10	blaTEM_1B.1_JF910132, strA.4_AF321551, strB.1_M96392, sul2.3_HQ840942, tet_B_.4_AF326777
S4812-10	aadA1.3_JQ414041, aadA2.1_X68227, blaTEM_1B.1_JF910132, cmlA1.1_M64556, dfrA12.1_AB571791, sul1.2_CP002151, sul3.2_AJ459418
S4489-10	
S00814-10	blaTEM_1B.1_JF910132, strA.4_AF321551, strB.1_M96392, sul2.3_HQ840942, tet_B_.4_AF326777
S03445-08	blaTEM_1B.1_JF910132, strA.4_AF321551, strB.1_M96392, sul2.3_HQ840942, tet_B_.4_AF326777
S07300-05	blaTEM_1B.1_JF910132, strA.4_AF321551, strB.1_M96392, sul2.3_HQ840942, tet_B_.4_AF326777
S00065-06	blaTEM_1B.1_JF910132, strA.4_AF321551, strB.1_M96392, sul2.3_HQ840942, tet_B_.4_AF326777
S01364-10	blaTEM_1B.1_JF910132, strA.4_AF321551, strB.1_M96392, sul2.3_HQ840942, tet_B_.4_AF326777
4824-10	
4797-10	
S5712-08	
S5828-08	aadA2.1_X68227, blaCARB_2.1_M69058, floR.2_AF118107, sul1.2_CP002151, tet_G_.5_AF071555

S03113-10	blaTEM_1B.1_JF910132, strA.4_AF321551, strB.1_M96392, sul2.3_HQ840942, tet_B_.4_AF326777
S04698-09	blaTEM_1B.1_JF910132, strA.4_AF321551, strB.1_M96392, sul2.3_HQ840942, tet_B_.4_AF326777
L00759-09	tet_B_.4_AF326777
S3659-10	
S00250-07	
S00176-09	blaTEM_1B.1_JF910132, strA.4_AF321551, strB.1_M96392, sul2.3_HQ840942, tet_B_.4_AF326777
L00961-04	tet_A_.4_AJ517790
5102-1999	aadA1.2_JN815078, aadA2.1_X68227, cmlA1.1_M64556, sul1.1_AY224185, sul2.2_GQ421466, sul3.2_AJ459418
L01730-06	blaTEM_1B.1_JF910132, strA.4_AF321551, strB.1_M96392, sul2.3_HQ840942, tet_B_.4_AF326777
S02909-08	blaTEM_1B.1_JF910132, strA.4_AF321551, strB.1_M96392, sul2.3_HQ840942, tet_B_.4_AF326777
S05893-09	blaTEM_1B.1_JF910132, strA.4_AF321551, strB.1_M96392, sul2.3_HQ840942, tet_B_.4_AF326777
S05894-09	blaTEM_1B.1_JF910132, strA.4_AF321551, strB.1_M96392, sul2.3_HQ840942, tet_B_.4_AF326777
H105100366	blaTEM_1B.1_JF910132, dfrA14.1_DQ388123, strB.1_M96392, sul2.2_GQ421466, tet_A_.4_AJ517790
H105260826	blaTEM_1B.1_JF910132, dfrA14.1_DQ388123, strB.1_M96392, sul2.2_GQ421466, tet_A_.4_AJ517790
H105280433	blaTEM_1B.1_JF910132, strA.4_AF321551, strB.1_M96392, sul2.3_HQ840942, tet_B_.4_AF326777
H103260370	blaTEM_1B.1_JF910132, strA.4_AF321551, strB.1_M96392, sul2.3_HQ840942, tet_B_.4_AF326777
H103700509	
H103720606	aadA1.2_JN815078, blaTEM_1B.1_JF910132, cmlA1.1_M64556, dfrA12.1_AB571791, sul1.1_AY224185, sul2.2_GQ421466, sul3.2_AJ459418, tet_A_.4_AJ517790
H103920583	blaTEM_1B.1_JF910132, strA.4_AF321551, strB.1_M96392, sul2.3_HQ840942
H1041406001	
H104240404	blaTEM_1B.1_JF910132, strA.4_AF321551, strB.1_M96392, sul2.3_HQ840942, tet_B_.4_AF326777
H104680513	blaTEM_1B.1_JF910132, strA.4_AF321551, strB.1_M96392, sul2.3_HQ840942, tet_B_.4_AF326777
H105000301	aadA1.5_JX185132, aph_4__Ia.1_V01499, blaTEM_1B.1_JF910132, dfrA1.30_JQ690541, mph_B_.1_D85892, strA.4_AF321551, strB.1_M96392, sul1.1_AY224185, sul2.3_HQ840942, tet_B_.4_AF326777
2200/2	blaTEM_1B.1_JF910132, strA.4_AF321551, strB.1_M96392, sul2.3_HQ840942, tet_B_.4_AF326777, gyrA.D87G
2448/2	blaTEM_1B.1_JF910132, strA.4_AF321551, strB.1_M96392, sul2.3_HQ840942, tet_B_.4_AF326777
1038/2	blaTEM_1B.1_JF910132, sul1.2_CP002151, sul2.2_GQ421466
496/10	blaTEM_1B.1_JF910132, strA.4_AF321551, strB.1_M96392, sul2.3_HQ840942

1115/25	aadA1.2_JN815078, aadA2.1_X68227, blaTEM_1B.1_JF910132, cmlA1.1_M64556, dfrA12.1_AB571791, sul1.2_CP002151, sul2.2_GQ421466, sul3.2_AJ459418, tet_A_.4_AJ517790
1686/1	blaTEM_1B.1_JF910132, strA.4_AF321551, strB.1_M96392, sul2.3_HQ840942, tet_B_.4_AF326777
1790/1	aadA1.3_JQ414041, aadA2.1_X68227, cmlA1.1_M64556, sul3.2_AJ459418
45/16	blaTEM_1B.1_JF910132, strA.4_AF321551, strB.1_M96392, sul2.3_HQ840942, tet_B_.4_AF326777, gyrA.D87N
2617/20	blaTEM_1B.1_JF910132, strA.4_AF321551, strB.1_M96392, sul2.3_HQ840942, tet_B_.4_AF326777
1948/2	
1693/1	tet_B_.4_AF326777
242/2	blaTEM_1B.1_JF910132, strA.4_AF321551, strB.1_M96392, sul2.3_HQ840942, tet_B_.4_AF326777, gyrA.S83Y
3046/11	blaTEM_1B.1_JF910132, strA.4_AF321551, strB.1_M96392, sul2.3_HQ840942, tet_B_.4_AF326777
1365/1	
2841/2	blaTEM_1B.1_JF910132, strA.4_AF321551, strB.1_M96392, sul2.3_HQ840942, tet_B_.4_AF326777
692/26	aph_4__Ia.1_V01499, blaTEM_1B.1_JF910132, floR.2_AF118107, strA.4_AF321551, strB.1_M96392, sul2.3_HQ840942, tet_B_.4_AF326777
2117/2	blaTEM_1B.1_JF910132, strA.4_AF321551, strB.1_M96392, sul2.3_HQ840942, tet_B_.4_AF326777
629/2	blaTEM_1B.1_JF910132, strA.4_AF321551, strB.1_M96392, sul2.3_HQ840942
2223/2	blaTEM_1B.1_JF910132, strA.4_AF321551, strB.1_M96392, sul2.3_HQ840942, tet_B_.4_AF326777
H07 246 0339	blaTEM_1B.1_JF910132, strA.1_M96392, strB.1_M96392, sul2.3_HQ840942, tet_B_.4_AF326777
H07 362 0321	blaTEM_1B.1_JF910132, strA.4_AF321551, strB.1_M96392, sul2.3_HQ840942, tet_B_.4_AF326777
H090260055	tet_B_.4_AF326777
H100120548	
H100420171	
H100760028	
H100800267	strA.4_AF321551, strB.1_M96392, sul2.3_HQ840942, tet_B_.4_AF326777
H101020440	tet_B_.4_AF326777
H101560198	
H07 016 0417	tet_B_.4_AF326777
H102120667	blaTEM_1B.1_JF910132, strA.4_AF321551, strB.1_M96392, sul2.3_HQ840942
H10234093302	blaTEM_1B.1_JF910132, dfrA1.1_X00926, strA.4_AF321551, strB.1_M96392, sul2.3_HQ840942
H07 166 0082	blaTEM_1B.1_JF910132, strA.4_AF321551, strB.1_M96392, sul2.3_HQ840942, tet_B_.4_AF326777

H07 182 0182	blaTEM_1B.1_JF910132, strA.4_AF321551, strB.1_M96392, sul2.3_HQ840942, tet_B_.4_AF326777
H07 230 0280	blaTEM_1B.1_JF910132, strA.4_AF321551, strB.1_M96392, sul2.3_HQ840942, tet_B_.4_AF326777
H07 234 0179	
H07 246 0338	blaTEM_1B.1_JF910132, strA.4_AF321551, strB.1_M96392, sul2.3_HQ840942, tet_B_.4_AF326777
H07 276 0382	blaTEM_1B.1_JF910132, strA.4_AF321551, strB.1_M96392, sul2.3_HQ840942, tet_B_.4_AF326777
H07 338 0264	aac_3__IIa.1_X51534, aadA2.1_X68227, aph_3___Ic.1_X62115, blaTEM_1A.4_HM749966, dfrA12.1_AB571791, floR.2_AF118107, strA.4_AF321551, strB.1_M96392, sul2.2_GQ421466, tet_B_.4_AF326777, tet_M_.4_X75073
H07 394 0379	blaTEM_1B.1_JF910132, strA.4_AF321551, strB.1_M96392, sul2.3_HQ840942, tet_B_.4_AF326777
VNDSal1	
VNS10052	ARR_3.1_JF806499, aac_6__Ib_cr.1_DQ303918, aadA1.3_JQ414041, aph_3___Ia.1_V00359, aph_4__Ia.1_V01499, blaOXA_1.1_J02967, catB3.1_AJ009818, cmlA1.1_M64556, dfrA12.1_AB571791, floR.2_AF118107, oqxA.1_EU370913, oqxB.1_EU370913, sul1.1_AY224185, sul2.2_GQ421466, sul3.2_AJ459418, tet_B_.4_AF326777
VNS20005	tet_B_.4_AF326777
VNS20207	ARR_3.1_JF806499, QnrS1.1_AB187515, QnrS2.1_JF261185, aadA1.3_JQ414041, aadA2.1_X68227, blaTEM_1B.1_JF910132, catB3.1_AJ009818, cmlA1.1_M64556, dfrA12.1_AB571791, floR.2_AF118107, sul2.2_GQ421466, sul3.2_AJ459418, tet_A_.4_AJ517790, tet_B_.4_AF326777, tet_M_.8_X04388
VNS30099	QnrS1.1_AB187515, QnrS2.1_JF261185, aadA1.3_JQ414041, aadA2.1_X68227, blaOXA_1.1_J02967, blaTEM_1B.1_JF910132, catB3.1_AJ009818, cmlA1.1_M64556, dfrA12.1_AB571791, strA.4_AF321551, strB.1_M96392, sul1.10_DQ143913, sul2.3_HQ840942, sul3.2_AJ459418, tet_A_.4_AJ517790, tet_B_.4_AF326777, tet_M_.8_X04388
VNS121 DQT	aadA2.2_JQ364967, aph_3___Ia.1_V00359, dfrA12.1_AB571791, strA.4_AF321551, strB.1_M96392, sul2.3_HQ840942, tet_B_.4_AF326777, gyrA.S83Y
VNS10314	blaTEM_1B.1_JF910132, strA.4_AF321551, strB.1_M96392, sul2.2_GQ421466, tet_B_.4_AF326777
VNB455	blaTEM_1B.1_JF910132, strA.4_AF321551, strB.1_M96392, sul2.3_HQ840942, tet_B_.4_AF326777
VNS10045	ARR_3.1_JF806499, aac_6__Ib_cr.1_DQ303918, aadA1.3_JQ414041, blaOXA_1.1_J02967, catB3.1_AJ009818, cmlA1.1_M64556, dfrA12.1_AB571791, oqxA.1_EU370913, oqxB.1_EU370913, sul1.2_CP002151, sul2.3_HQ840942, sul3.2_AJ459418, tet_B_.4_AF326777, gyrA.D87N
VNB712	ARR_3.1_JF806499, aac_6__Ib_cr.1_DQ303918_2, aph_4__Ia.1_V01499, blaOXA_1.1_J02967, catB3.1_AJ009818, floR.2_AF118107, sul1.1_AY224185, sul2.2_GQ421466, tet_B_.4_AF326777
VNB1222	ARR_3.1_JF806499, aac_6__Ib_cr.1_DQ303918, aadA1.3_JQ414041, aph_3___Ia.1_V00359, blaOXA_1.1_J02967, catB3.1_AJ009818, cmlA1.1_M64556, dfrA12.1_AB571791, oqxA.1_EU370913, oqxB.1_EU370913, sul1.1_AY224185, sul3.2_AJ459418, tet_B_.4_AF326777
VNDSal2	
VNS10068	QnrS1.1_AB187515, aadA1.3_JQ414041, aadA2.1_X68227, blaTEM_1B.1_JF910132, cmlA1.1_M64556, dfrA12.1_AB571791, floR.2_AF118107, sul2.2_GQ421466, sul3.2_AJ459418, tet_A_.4_AJ517790, tet_M_.8_X04388
VNS20007	blaTEM_1B.1_JF910132, strA.4_AF321551, strB.1_M96392, sul2.3_HQ840942, tet_B_.4_AF326777
VNS20235	QnrS2.1_JF261185, blaTEM_1B.1_JF910132, strA.4_AF321551, strB.1_M96392, sul2.3_HQ840942, tet_B_.4_AF326777
VNS165 VDQ	aac_3__IId.1_EU022314, aadA2.2_JQ364967, aph_3___Ia.1_V00359, blaTEM_1B.1_JF910132, dfrA12.1_AB571791, strA.4_AF321551, strB.1_M96392, sul2.3_HQ840942, tet_B_.4_AF326777

VNS10413	ARR_3.1_JF806499, aac_6__Ib_cr.1_DQ303918, aadA1.3_JQ414041, aadA2.1_X68227, aph_4__Ia.1_V01499, blaOXA_1.1_J02967, catB3.1_AJ009818, cmlA1.1_M64556, floR.2_AF118107, sul1.1_AY224185, sul2.2_GQ421466, sul3.2_AJ459418, tet_B_.4_AF326777
VNB148	aadA2.1_X68227, blaCARB_2.1_M69058, floR.2_AF118107, sul1.1_AY224185, tet_G_.4_AF133140, gyrA.S83F
VNB541	ARR_3.1_JF806499, aac_6__Ib_cr.1_DQ303918, aadA1.3_JQ414041, aph_4__Ia.1_V01499, blaOXA_1.1_J02967, catB3.1_AJ009818, cmlA1.1_M64556, dfrA12.1_AB571791, floR.2_AF118107, oqxA.1_EU370913, oqxB.1_EU370913, sul1.2_CP002151, sul2.2_GQ421466, sul3.2_AJ459418, tet_B_.4_AF326777
VNB745	gyrA.D87N
VNB1264	ARR_3.1_JF806499, aadA1.3_JQ414041, aph_4__Ia.1_V01499, blaOXA_1.1_J02967, catB3.1_AJ009818, cmlA1.1_M64556, dfrA12.1_AB571791, floR.2_AF118107, oqxA.1_EU370913, oqxB.1_EU370913, sul1.2_CP002151, sul2.2_GQ421466, sul3.2_AJ459418, tet_B_.4_AF326777, gyrA.D87N
VNDSal3	
VNB68	aac_3__IId.1_EU022314, aadA2.2_JQ364967, aph_3___Ia.1_V00359, blaTEM_1B.1_JF910132, dfrA12.1_AB571791, strA.4_AF321551, strB.1_M96392, sul2.3_HQ840942, tet_B_.4_AF326777
VNS10124	
VNS20018	QnrS1.1_AB187515, aadA1.3_JQ414041, aadA2.1_X68227, blaTEM_1B.1_JF910132, cmlA1.1_M64556, dfrA12.1_AB571791, floR.2_AF118107, sul2.2_GQ421466, sul3.2_AJ459418, tet_A_.4_AJ517790, tet_M_.8_X04388
VNS20277	QnrS1.1_AB187515, aadA1.3_JQ414041, aadA2.1_X68227, blaTEM_1B.1_JF910132, cmlA1.1_M64556, dfrA12.1_AB571791, floR.2_AF118107, sul2.2_GQ421466, sul3.2_AJ459418, tet_A_.4_AJ517790, tet_M_.8_X04388
VNS20150	QnrS1.1_AB187515
VNS30144	ARR_3.1_JF806499, aac_6__Ib_cr.1_DQ303918, aadA1.3_JQ414041, aadA2.1_X68227, aph_3___Ia.1_V00359, aph_4__Ia.1_V01499, blaOXA_1.1_J02967, blaTEM_1B.1_JF910132, catB3.1_AJ009818, cmlA1.1_M64556, floR.2_AF118107, oqxA.1_EU370913, oqxB.1_EU370913, strA.4_AF321551, strB.1_M96392, sul1.1_AY224185, sul2.2_GQ421466, sul3.2_AJ459418, tet_B_.4_AF326777, gyrA.D87N
VNSC2442	QnrS1.1_AB187515, strA.4_AF321551, strB.1_M96392, sul2.2_GQ421466, tet_A_.4_AJ517790
VNB151	ARR_3.1_JF806499, aac_6__Ib_cr.1_DQ303918_2, aadA1.3_JQ414041, aadA2.1_X68227, aph_3___Ia.1_V00359, aph_4__Ia.1_V01499, blaOXA_1.1_J02967, catB3.1_AJ009818, cmlA1.1_M64556, floR.2_AF118107, oqxA.1_EU370913, oqxB.1_EU370913, sul1.2_CP002151, sul2.2_GQ421466, sul3.2_AJ459418, tet_B_.4_AF326777
VNB589	ARR_3.1_JF806499, aac_6__Ib_cr.1_DQ303918, aadA1.3_JQ414041, aph_4__Ia.1_V01499, blaOXA_1.1_J02967, catB3.1_AJ009818, cmlA1.1_M64556, dfrA12.1_AB571791, floR.2_AF118107, oqxA.1_EU370913, oqxB.1_EU370913, sul1.2_CP002151, sul2.2_GQ421466, sul3.2_AJ459418
VNB773	aadA2.1_X68227, blaTEM_1B.1_JF910132, catA2.1_X53796, dfrA12.1_AB571791, strA.4_AF321551, strB.1_M96392, sul2.2_GQ421466, tet_A_.4_AJ517790, gyrA.D87Y
VNB1403	dfrA14.1_DQ388123
VNDSal4	
VNB1701	ARR_3.1_JF806499, aac_6__Ib_cr.1_DQ303918, aph_4__Ia.1_V01499, blaOXA_1.1_J02967, catB3.1_AJ009818, floR.2_AF118107, oqxA.1_EU370913, oqxB.1_EU370913, sul1.2_CP002151, sul2.2_GQ421466, tet_B_.4_AF326777, gyrA.D87N
VNS10137	aadA2.1_X68227, blaTEM_1B.1_JF910132, dfrA12.1_AB571791, strA.4_AF321551, strB.1_M96392, sul2.2_GQ421466, tet_A_.4_AJ517790, gyrA.D87Y
VNS30015	QnrS1.1_AB187515, aadA1.3_JQ414041, aadA2.1_X68227, blaTEM_1B.1_JF910132, cmlA1.1_M64556, dfrA12.1_AB571791, floR.2_AF118107, sul2.2_GQ421466, sul3.2_AJ459418, tet_A_.4_AJ517790, tet_M_.8_X04388
VNS20057	

VNS20278	aac_3__IId.1_EU022314, aadA2.2_JQ364967, aph_3___Ia.1_V00359, blaTEM_1B.1_JF910132, dfrA12.1_AB571791, floR.2_AF118107, strA.4_AF321551, strB.1_M96392, sul2.3_HQ840942, tet_B_.4_AF326777, gyrA.D87Y
VNS30161	aac_3__IId.1_EU022314, aadA2.2_JQ364967, aph_3___Ia.1_V00359, blaTEM_1B.1_JF910132, dfrA12.1_AB571791, strA.4_AF321551, strB.1_M96392, sul2.2_GQ421466, tet_A_.4_AJ517790, tet_B_.4_AF326777
VNSC2045	blaTEM_1B.1_JF910132, strA.4_AF321551, strB.1_M96392, sul2.3_HQ840942, tet_B_.4_AF326777
VNB170	aac_3__IId.1_EU022314, aadA2.2_JQ364967, aph_3___Ia.1_V00359, blaTEM_1B.1_JF910132, dfrA12.1_AB571791, strA.4_AF321551, strB.1_M96392, sul2.3_HQ840942, tet_B_.4_AF326777
VNB596	aadA2.1_X68227, blaCARB_2.1_M69058, floR.2_AF118107, sul1.1_AY224185, tet_G_.5_AF071555, gyrA.S83F
VNB802	ARR_3.1_JF806499, aac_6__Ib_cr.1_DQ303918_2, aadA1.3_JQ414041, aph_3___Ia.1_V00359, aph_4__Ia.1_V01499, blaOXA_1.1_J02967, catB3.1_AJ009818, cmlA1.1_M64556, dfrA12.1_AB571791, floR.2_AF118107, oqxA.1_EU370913, oqxB.1_EU370913, sul1.1_AY224185, sul2.2_GQ421466, sul3.2_AJ459418, tet_B_.4_AF326777
VNB1428	QnrS1.1_AB187515, aadA1.3_JQ414041, aadA2.1_X68227, blaTEM_1B.1_JF910132, cmlA1.1_M64556, dfrA12.1_AB571791, floR.2_AF118107, sul2.2_GQ421466, sul3.2_AJ459418, tet_A_.4_AJ517790, tet_M_.8_X04388
VNDSal5	
VNS30385	ARR_3.1_JF806499, aac_6__Ib_cr.1_DQ303918_2, aadA1.3_JQ414041, aadA2.1_X68227, aph_3___Ia.1_V00359, aph_4__Ia.1_V01499, blaOXA_1.1_J02967, catB3.1_AJ009818, cmlA1.1_M64556, floR.2_AF118107, sul1.2_CP002151, sul2.2_GQ421466, sul3.2_AJ459418, tet_B_.4_AF326777
VNS10146	ARR_3.1_JF806499, QnrS2.1_JF261185, aac_6__Ib_cr.1_DQ303918, aadA1.3_JQ414041, aadA2.1_X68227, aph_3___Ia.1_V00359, aph_4__Ia.1_V01499, blaOXA_1.1_J02967, catB3.1_AJ009818, cmlA1.1_M64556, dfrA12.1_AB571791, oqxA.1_EU370913, oqxB.1_EU370913, sul1.1_AY224185, sul2.2_GQ421466, sul3.2_AJ459418, tet_B_.4_AF326777
VNDSal8	
VNS20337	QnrS1.1_AB187515, aadA1.3_JQ414041, aadA2.1_X68227, blaTEM_1B.1_JF910132, cmlA1.1_M64556, dfrA12.1_AB571791, floR.2_AF118107, sul2.2_GQ421466, sul3.2_AJ459418, tet_A_.4_AJ517790, tet_M_.8_X04388
VNS30243	blaTEM_1B.1_JF910132, strA.4_AF321551, strB.1_M96392, sul2.3_HQ840942, tet_B_.4_AF326777
VNSC2047	strA.4_AF321551, strB.1_M96392, sul2.2_GQ421466, tet_A_.4_AJ517790, gyrA.D87Y
VNB176	ARR_3.1_JF806499, aac_6__Ib_cr.1_DQ303918_2, aadA1.3_JQ414041, aadA2.1_X68227, aph_3___Ia.1_V00359, aph_4__Ia.1_V01499, blaOXA_1.1_J02967, blaTEM_1B.1_JF910132, catB3.1_AJ009818, cmlA1.1_M64556, dfrA12.1_AB571791, floR.2_AF118107, oqxA.1_EU370913, oqxB.1_EU370913, strA.4_AF321551, strB.1_M96392, sul1.1_AY224185, sul2.2_GQ421466, sul3.2_AJ459418, tet_A_.4_AJ517790, tet_B_.4_AF326777
VNB617	ARR_3.1_JF806499, aac_6__Ib_cr.1_DQ303918, aph_4__Ia.1_V01499, blaOXA_1.1_J02967, catB3.1_AJ009818, floR.2_AF118107, oqxA.1_EU370913, oqxB.1_EU370913, sul1.1_AY224185, sul2.2_GQ421466, tet_B_.4_AF326777
VNB845	catA2.1_X53796, oqxA.1_EU370913, oqxB.1_EU370913, sul1.2_CP002151, tet_B_.4_AF326777
VNB1436	aacA4.1_KM278199, blaTEM_1B.1_JF910132, oqxA.1_EU370913, oqxB.1_EU370913, strA.4_AF321551, strB.1_M96392, sul2.3_HQ840942, tet_B_.4_AF326777
VNSC2362	strA.4_AF321551, strB.1_M96392, sul2.2_GQ421466, tet_A_.4_AJ517790
VNDSal6	
VNS10182	ARR_3.1_JF806499, aac_6__Ib_cr.1_DQ303918, aadA1.3_JQ414041, aph_3___Ia.1_V00359, aph_4__Ia.1_V01499, blaOXA_1.1_J02967, catB3.1_AJ009818, cmlA1.1_M64556, dfrA12.1_AB571791, floR.2_AF118107, sul1.1_AY224185, sul2.2_GQ421466, sul3.2_AJ459418, tet_B_.4_AF326777

VNS20101	blaTEM_1B.1_JF910132, strA.4_AF321551, strB.1_M96392, sul2.3_HQ840942, tet_B_.4_AF326777
VNS20480	
VNS30267	QnrS1.1_AB187515, aadA1.3_JQ414041, aadA2.1_X68227, blaTEM_1B.1_JF910132, cmlA1.1_M64556, dfrA12.1_AB571791, floR.2_AF118107, sul2.2_GQ421466, sul3.2_AJ459418, tet_A_.4_AJ517790
VNSC2191	blaTEM_1B.1_JF910132, strA.4_AF321551, strB.1_M96392, sul2.3_HQ840942, tet_A_.4_AJ517790, tet_B_.4_AF326777
VNB177	ARR_3.1_JF806499, aac_3__IId.1_EU022314, aac_6__Ib_cr.1_DQ303918, aadA1.3_JQ414041, aadA2.1_X68227, aph_3___Ia.1_V00359, aph_4__Ia.1_V01499, blaOXA_1.1_J02967, catB3.1_AJ009818, cmlA1.1_M64556, floR.2_AF118107, sul1.1_AY224185, sul2.2_GQ421466, sul3.2_AJ459418, tet_B_.4_AF326777
VNB652	aadA2.1_X68227, aph_4__Ia.1_V01499, blaTEM_1B.1_JF910132, dfrA12.1_AB571791, floR.2_AF118107, strA.4_AF321551, strB.1_M96392, sul2.2_GQ421466, tet_A_.4_AJ517790, tet_M_.8_X04388, gyrA.D87Y
VNB198	aac_3__IId.1_EU022314, aadA1.3_JQ414041, aadA2.1_X68227, blaTEM_1B.1_JF910132, cmlA1.1_M64556, strA.4_AF321551, strB.1_M96392, sul2.2_GQ421466, sul3.2_AJ459418, tet_A_.4_AJ517790, gyrA.D87N
VNB922	ARR_3.1_JF806499, aac_6__Ib_cr.1_DQ303918, aadA1.3_JQ414041, aadA2.1_X68227, aph_3___Ia.1_V00359, aph_4__Ia.1_V01499, blaOXA_1.1_J02967, blaTEM_1B.1_JF910132, catB3.1_AJ009818, cmlA1.1_M64556, dfrA12.1_AB571791, floR.2_AF118107, oqxA.1_EU370913, oqxB.1_EU370913, strA.4_AF321551, strB.1_M96392, sul1.2_CP002151, sul2.2_GQ421466, sul3.2_AJ459418, tet_A_.4_AJ517790
VNB1479	QnrS1.1_AB187515, aac_3__IId.1_EU022314, aadA1.3_JQ414041, aadA2.1_X68227, blaTEM_1B.1_JF910132, cmlA1.1_M64556, dfrA12.1_AB571791, floR.2_AF118107, mef_B_.1_FJ196385, strA.4_AF321551, strB.1_M96392, sul2.3_HQ840942, sul3.2_AJ459418, tet_B_.4_AF326777
VNDSal7	
VNS20099	blaTEM_1B.1_JF910132, dfrA12.1_AB571791, strA.4_AF321551, strB.1_M96392, sul2.3_HQ840942, tet_B_.4_AF326777
VNS30012	
VNS30356	blaTEM_1B.1_JF910132, strA.4_AF321551, strB.1_M96392, sul2.3_HQ840942, tet_B_.4_AF326777
VNSC2235	
VNB692	ARR_3.1_JF806499, aac_6__Ib_cr.1_DQ303918, aadA1.3_JQ414041, aadA2.1_X68227, aph_3___Ia.1_V00359, blaOXA_1.1_J02967, catB3.1_AJ009818, cmlA1.1_M64556, dfrA12.1_AB571791, floR.2_AF118107, oqxA.1_EU370913, oqxB.1_EU370913, sul1.2_CP002151, sul2.2_GQ421466, sul3.2_AJ459418, tet_B_.4_AF326777
VNB184	aac_3__IId.1_EU022314, aadA2.2_JQ364967, aph_3___Ia.1_V00359, blaTEM_1B.1_JF910132, dfrA12.1_AB571791, strA.4_AF321551, strB.1_M96392, sul2.3_HQ840942, tet_B_.4_AF326777
VNB664	aadA2.1_X68227, blaTEM_1B.1_JF910132, catA2.1_X53796, dfrA12.1_AB571791, strA.4_AF321551, strB.1_M96392, sul2.2_GQ421466, tet_A_.4_AJ517790, gyrA.D87Y
VNB1140	ARR_3.1_JF806499, aac_6__Ib_cr.1_DQ303918_2, aadA1.3_JQ414041, aph_3___Ia.1_V00359, aph_4__Ia.1_V01499, blaOXA_1.1_J02967, catB3.1_AJ009818, cmlA1.1_M64556, dfrA12.1_AB571791, floR.2_AF118107, oqxA.1_EU370913, oqxB.1_EU370913, sul1.1_AY224185, sul2.2_GQ421466, sul3.2_AJ459418, tet_B_.4_AF326777, gyrA.D87N
VNB1505	ARR_3.1_JF806499, aac_6__Ib_cr.1_DQ303918_2, aadA1.3_JQ414041, aph_4__Ia.1_V01499, blaOXA_1.1_J02967, catB3.1_AJ009818, cmlA1.1_M64556, dfrA12.1_AB571791, floR.2_AF118107, oqxA.1_EU370913, oqxB.1_EU370913, sul1.2_CP002151, sul2.2_GQ421466, sul3.2_AJ459418, tet_B_.4_AF326777
VNS20081	ARR_3.1_JF806499, aac_6__Ib_cr.1_DQ303918_2, aadA1.3_JQ414041, aadA2.1_X68227, aph_3___Ia.1_V00359, aph_4__Ia.1_V01499, blaCMY_2.1_X91840, blaOXA_1.1_J02967, catB3.1_AJ009818, cmlA1.1_M64556, dfrA12.1_AB571791, floR.2_AF118107, oqxA.1_EU370913, oqxB.1_EU370913, sul1.1_AY224185, sul2.2_GQ421466, sul3.2_AJ459418, tet_B_.4_AF326777, gyrA.D87N
VNB1166	blaTEM_1B.1_JF910132, strA.4_AF321551, strB.1_M96392, sul2.3_HQ840942, tet_B_.4_AF326777

S15	tet_B_.4_AF326777
S23	tet_B_.4_AF326777
S7	tet_B_.4_AF326777
ST111849	aadA1.3_JQ414041, blaOXA_1.1_J02967, catA1.1_V00622, sul1.2_CP002151, tet_B_.4_AF326777
ST1489	
ST1660	aadA1.3_JQ414041, aph_3___IIa.1_X57709, blaOXA_1.1_J02967, catA1.1_V00622, dfrA12.1_AB571791, strA.4_AF321551, strB.1_M96392, sul1.2_CP002151, sul2.2_GQ421466, tet_B_.4_AF326777, gyrA.S83F, parC.S80R, parE.S458P
ST2143	aac_3__IId.1_EU022314, aadA2.2_JQ364967, aph_3___IIa.1_X57709, blaOXA_1.1_J02967, catA1.1_V00622, dfrA12.1_AB571791, rmtB.1_AB103506, tet_B_.4_AF326777, tet_G_.5_AF071555, gyrA.S83F, parC.S80R, parE.S458P
ST2286	aac_3__IId.1_EU022314, aadA2.2_JQ364967, blaTEM_1B.1_JF910132, dfrA12.1_AB571791, strA.4_AF321551, strB.1_M96392, sul2.3_HQ840942, tet_B_.4_AF326777
ST2287	aac_3__IId.1_EU022314, aadA2.2_JQ364967, blaOXA_1.1_J02967, catA1.1_V00622, dfrA12.1_AB571791, sul1.2_CP002151, tet_B_.4_AF326777
ST2533	aac_3__IId.1_EU022314, aadA2.2_JQ364967, blaTEM_1B.1_JF910132, dfrA12.1_AB571791, strB.1_M96392, sul2.3_HQ840942, tet_B_.4_AF326777, gyrA.D87N
ST2850	aac_3__IId.1_EU022314, gyrA.S83Y
ST3363	
ST372	aac_3__IId.1_EU022314, aadA1.5_JX185132, aph_3___IIa.1_X57709, blaOXA_1.1_J02967, catA1.1_V00622, strB.1_M96392, sul1.1_AY224185, sul2.2_GQ421466, tet_B_.4_AF326777, gyrA.S83F, parC.S80R, parE.S458P
ST3858	tet_A_.4_AJ517790
ST4024	oqxB.1_EU370913, tet_B_.4_AF326777, gyrA.D87Y
ST4038	
ST4329	gyrA.D87N
ST4650	
ST4848	aac_3__IId.1_EU022314, blaOXA_1.1_J02967, catA1.1_V00622, tet_B_.4_AF326777, gyrA.S83F, parC.S80R
ST486	
ST6988	aadA1.3_JQ414041, aadB.1_JN119852, cmlA1.1_M64556, mph_A_.1_D16251, strA.1_M96392, strB.1_M96392, tet_B_.4_AF326777
ST728	
ST8493	catA1.1_V00622, strB.1_M96392, sul2.3_HQ840942, tet_B_.4_AF326777
