# Supplementary material for: New Variant of Multidrug-Resistant Salmonella enterica Serovar Typhimurium Associated with Invasive Disease in Immunocompromised Patients in Vietnam
Source: mBio. 2018 Sep 4;9(5):e01056-18. doi: 10.1128/mBio.01056-18 (PMC6123440; doi:10.1128/mBio.01056-18)
Supplement: TABLE S6 [file mbo004184053st6.pdf]

Table S6. Incompatibility (Inc) types of plasmids identified in the isolates from Vietnam and the context collection.

Isolate	Inc type
73_V_253	IncFIB_S_.1__FN432031, IncFII_S_.1__CP000858
73_V_282	IncFIB_S_.1__FN432031, IncFII_S_.1__CP000858
73_V_286	IncFIB_S_.1__FN432031, IncFII_S_.1__CP000858, IncX1.4__JN935898
73_V_283	IncFIB_S_.1__FN432031, IncFII_S_.1__CP000858, IncX1.4__JN935898, IncX1.2__CP003417
73_V_285	IncFIB_S_.1__FN432031, IncFII_S_.1__CP000858, IncX1.4__JN935898
73_V_320	IncFIB_S_.1__FN432031, IncFII_S_.1__CP000858
73_V_321	IncFIB_S_.1__FN432031, IncFII_S_.1__CP000858
73_V_322	IncFIB_S_.1__FN432031, IncFII_S_.1__CP000858
73_V_336	IncFIB_S_.1__FN432031, IncFII_S_.1__CP000858
73_V_335	IncFIB_S_.1__FN432031, IncFII_S_.1__CP000858
73_V_020	IncFIB_S_.1__FN432031, IncFII_S_.1__CP000858
73_V_364	Col_BS512_.1__NC_010656, Col_BS512_.1__NC_010656.2, IncFIA_HI1_.1_HI1_AF250878, IncHI1A.1__AF250878, IncHI1B_R27_.1_R27_AF250878
73_V_381	IncFIB_S_.1__FN432031, IncFII_S_.1__CP000858, IncX1.4__JN935898
73_V_425	IncFIB_S_.1__FN432031, IncFII_S_.1__CP000858, IncX1.4__JN935898, IncX1.2__CP003417
73_V_423	IncFIB_S_.1__FN432031, IncFII_S_.1__CP000858
73_V_426	IncFIB_S_.1__FN432031, IncFII_S_.1__CP000858, IncX1.4__JN935898, IncX1.2__CP003417
74_V_100	IncFIB_S_.1__FN432031, IncFII_S_.1__CP000858
74_V_101	IncFIB_S_.1__FN432031, IncFII_S_.1__CP000858
74_V_102	IncFIB_S_.1__FN432031, IncFII_S_.1__CP000858
74_V_129	IncFIB_S_.1__FN432031, IncFII_S_.1__CP000858
74_V_202	IncFIB_S_.1__FN432031, IncFII_S_.1__CP000858
73_V_023	IncFIB_S_.1__FN432031, IncFII_S_.1__CP000858
74_V_217	IncFIB_S_.1__FN432031, IncFII_S_.1__CP000858
74_V_235	IncHI2.1__BX664015, IncHI2A.1__BX664015
74_V_310	IncFIB_S_.1__FN432031, IncFII_S_.1__CP000858

74_V_368	IncFIB_S_.1__FN432031, IncFII_S_.1__CP000858
74_V_419	IncFIB_S_.1__FN432031, IncFII_S_.1__CP000858, IncQ1.1__HE654726
74_V_418	IncFIB_S_.1__FN432031, IncFII_S_.1__CP000858
71_G_169	IncHI1A.1__AF250878, IncHI1B_R27_.1_R27_AF250878, IncI1.1_Alpha_AP005147
71_G_450	IncA_C2.1__JN157804, IncQ1.1__HE654726
72-G-120	IncFIB_S_.1__FN432031, IncFII_S_.1__CP000858
73_G_051	IncQ1.1__HE654726
73_V_039	IncFIB_S_.1__FN432031, IncFII_S_.1__CP000858
73_G_047	IncQ1.1__HE654726
73_G_049	IncQ1.1__HE654726
73_G_050	IncQ1.1__HE654726
71_H_035	ColRI.1__DQ298019, IncHI2.1__BX664015, IncHI2A.1__BX664015, IncQ2.1__FJ696404
71_H_034	Col8282.1__DQ995353, ColRI.1__DQ298019, IncHI2.1__BX664015, IncHI2A.1__BX664015, IncQ2.1__FJ696404
71_H_053	IncQ1.1__HE654726
71_H_052	IncQ1.1__HE654726
71_H_051	IncQ1.1__HE654726
71_H_085	Col_BS512_.1__NC_010656, Col_BS512_.1__NC_010656.2, IncHI1A.1__AF250878, IncHI1B_R27_.1_R27_AF250878
71_H_083	Col_BS512_.1__NC_010656, Col_BS512_.1__NC_010656.2
73_V_038	IncFIB_S_.1__FN432031, IncFII_S_.1__CP000858
71_H_084	ColRI.1__DQ298019, IncHI1A.1__AF250878, IncHI1B_R27_.1_R27_AF250878
71_H_243	ColRI.1__DQ298019, ColpVC.1__JX133088, IncFIA_HI1_.1_HI1_AF250878, IncHI1A.1__AF250878, IncHI1B_R27_.1_R27_AF250878
72_H_033	ColRI.1__DQ298019, IncHI2.1__BX664015, IncHI2A.1__BX664015, IncQ2.1__FJ696404
72_H_265	IncHI2.1__BX664015, IncHI2A.1__BX664015, IncN.1__AY046276, IncQ1.1__HE654726
72_H_332	IncFII.1__AY458016, IncFIA.1__AP001918, IncI1.1_Alpha_AP005147
73_H_001	Col_BS512_.1__NC_010656, Col_BS512_.1__NC_010656.2, IncFIA_HI1_.1_HI1_AF250878, IncHI1A.1__AF250878, IncHI1B_R27_.1_R27_AF250878
73_H_110	Col_BS512_.1__NC_010656, Col_BS512_.1__NC_010656.2, IncFIA_HI1_.1_HI1_AF250878, IncHI1A.1__AF250878, IncHI1B_R27_.1_R27_AF250878
73_H_111	Col_BS512_.1__NC_010656, Col_BS512_.1__NC_010656.2, IncFIA_HI1_.1_HI1_AF250878, IncHI1A.1__AF250878, IncHI1B_R27_.1_R27_AF250878
73_H_112	Col_BS512_.1__NC_010656, Col_BS512_.1__NC_010656.2, IncFIA_HI1_.1_HI1_AF250878, IncHI1A.1__AF250878, IncHI1B_R27_.1_R27_AF250878

73_H_129	IncQ1.1__HE654726
73_V_065	IncFIB_S_.1__FN432031, IncFII_S_.1__CP000858
73_H_170	Col_BS512_.1__NC_010656, Col_BS512_.1__NC_010656.2, IncFIA_HI1_.1_HI1_AF250878, IncHI1A.1__AF250878, IncHI1B_R27_.1_R27_AF250878
73_H_172	IncA_C2.1__JN157804, IncQ1.1__HE654726
73_H_243	IncHI2.1__BX664015, IncHI2A.1__BX664015, IncN.1__AY046276, IncQ1.1__HE654726
74_H_047	ColRI.1__DQ298019, IncFIA_HI1_.1_HI1_AF250878, IncHI1A.1__AF250878, IncHI1B_R27_.1_R27_AF250878
74_H_072	IncHI2.1__BX664015, IncHI2A.1__BX664015
74_H_125	IncHI2.1__BX664015, IncHI2A.1__BX664015, IncQ1.1__HE654726
74_H_126	Col_BS512_.1__NC_010656, Col_BS512_.1__NC_010656.2, IncHI2.1__BX664015, IncHI2A.1__BX664015, IncQ1.1__HE654726
74_H_253	IncHI2.1__BX664015, IncHI2A.1__BX664015, IncN.1__AY046276, IncQ1.1__HE654726
74_H_256	IncHI2.1__BX664015, IncHI2A.1__BX664015, IncN.1__AY046276, IncQ1.1__HE654726
VNB1779	IncHI2.1__BX664015, IncHI2A.1__BX664015
73_V_110	IncFIB_S_.1__FN432031, IncFII_S_.1__CP000858, pEC4115.1__NC_01135
VNB1792	Col8282.1__DQ995353
VNB1870	IncFIB_S_.1__FN432031, IncFII_S_.1__CP000858
VNB2140	ColRI.1__DQ298019, IncHI2.1__BX664015, IncHI2A.1__BX664015
VNB2175	Col_BS512_.1__NC_010656, Col_BS512_.1__NC_010656.2, IncFIA_HI1_.1_HI1_AF250878, IncHI1A.1__AF250878, IncHI1B_R27_.1_R27_AF250878
VNB2200	IncFIB_S_.1__FN432031, IncFII_S_.1__CP000858
VNB2315	IncHI2.1__BX664015, IncHI2A.1__BX664015, IncQ1.1__HE654726
VNB2339	IncFIB_S_.1__FN432031, IncFII_S_.1__CP000858
VNB2605	IncHI2.1__BX664015, IncHI2A.1__BX664015, IncN.1__AY046276, IncQ1.1__HE654726
73_V_113	IncFIB_S_.1__FN432031, IncFII_S_.1__CP000858, pEC4115.1__NC_01135
Hue_11	IncHI2.1__BX664015, IncHI2A.1__BX664015
Hue_59	IncFIA_HI1_.1_HI1_AF250878, IncHI1A.1__AF250878, IncHI1B_R27_.1_R27_AF250878, IncQ1.1__HE654726
Hue_98	IncFIB_S_.1__FN432031, IncFII_S_.1__CP000858
KH_69	ColRI.1__DQ298019, IncFIA_HI1_.1_HI1_AF250878, IncHI1A.1__AF250878, IncHI1B_R27_.1_R27_AF250878
73_V_114	IncFIB_S_.1__FN432031, IncFII_S_.1__CP000858, IncX1.4__JN935898
73_V_168	IncFIB_S_.1__FN432031, IncFII_S_.1__CP000858, pEC4115.1__NC_01135

74_G_043	IncFIB_S_.1__FN432031, IncFII_S_.1__CP000858
74_G_339	Col_BS512_.1__NC_010656, Col_BS512_.1__NC_010656.2, IncFIA_HI1_.1_HI1_AF250878, IncHI1A.1__AF250878, IncHI1B_R27_.1_R27_AF250878
71_V_080	IncFIB_S_.1__FN432031, IncFII_S_.1__CP000858
71_V_097	IncFIB_S_.1__FN432031, IncFII_S_.1__CP000858, IncX1.4__JN935898
71_V_115	IncFIB_S_.1__FN432031, IncFII_S_.1__CP000858
71_V_330	IncFIB_S_.1__FN432031, IncFII_S_.1__CP000858, IncHI2.1__BX664015, IncHI2A.1__BX664015, IncX1.4__JN935898
71_V_389	IncFIB_S_.1__FN432031, IncFII_S_.1__CP000858
71_V_387	IncFIB_S_.1__FN432031, IncFII_S_.1__CP000858
71_V_385	IncFIB_S_.1__FN432031, IncFII_S_.1__CP000858
71_V_386	IncFIB_S_.1__FN432031, IncFII_S_.1__CP000858
71_V_466	IncFIB_S_.1__FN432031, IncFII_S_.1__CP000858, IncX1.4__JN935898, IncX1.2__CP003417
71_V_465	IncFIB_S_.1__FN432031, IncFII_S_.1__CP000858
71_V_480	IncFIB_S_.1__FN432031, IncFII_S_.1__CP000858
71_V_479	IncFIB_S_.1__FN432031, IncFII_S_.1__CP000858
71_V_478	IncFIB_S_.1__FN432031, IncFII_S_.1__CP000858
72_V_054	IncFIB_S_.1__FN432031, IncFII_S_.1__CP000858
72_V_055	IncFIB_S_.1__FN432031, IncFII_S_.1__CP000858
72_V_085	IncFIB_S_.1__FN432031, IncFII_S_.1__CP000858
72_V_082	IncFIB_S_.1__FN432031, IncFII_S_.1__CP000858
72_V_083	IncFIB_S_.1__FN432031, IncFII_S_.1__CP000858
72_V_111	IncFIB_S_.1__FN432031, IncFII_S_.1__CP000858
72_V_228	IncFIB_S_.1__FN432031, IncFII_S_.1__CP000858
72_V_267	IncFIB_S_.1__FN432031, IncFII_S_.1__CP000858
72_V_269	IncFIB_S_.1__FN432031, IncFII_S_.1__CP000858
72_V_282	IncFIB_S_.1__FN432031, IncFII_S_.1__CP000858
73_V_001	IncFIB_S_.1__FN432031, IncFII_S_.1__CP000858, IncX1.4__JN935898, IncX1.2__CP003417
MT13C.2.2	IncFIB_K_.1_Kpn3_JN233704, IncQ1.1__HE654726
CT49_2	IncA_C2.1__JN157804, IncQ1.1__HE654726

CT55_1	ColRI.1__DQ298019
CT69_2	IncQ1.1__HE654726
71_H_455	ColRI.1__DQ298019, IncHI2.1__BX664015, IncHI2A.1__BX664015, IncQ2.1__FJ696404
71_H_114	IncHI1A.1__AF250878, IncHI1B_R27_.1_R27_AF250878
71_H_228	ColRI.1__DQ298019, IncFIA_HI1_.1_HI1_AF250878, IncHI1A.1__AF250878, IncHI1B_R27_.1_R27_AF250878
71_V_202	IncFIB_S_.1__FN432031, IncFII_S_.1__CP000858
71_V_204	IncFIB_S_.1__FN432031, IncFII_S_.1__CP000858
71_V_313	IncFIB_S_.1__FN432031, IncFII_S_.1__CP000858
72-G-232	IncFIA_HI1_.1_HI1_AF250878, IncHI1A.1__AF250878, IncHI1B_R27_.1_R27_AF250878
20160374	IncHI2.1__BX664015, IncHI2A.1__BX664015, IncQ1.1__HE654726
20160407	IncFII_pRSB107_.1_pRSB107_AJ851089, IncHI2.1__BX664015, IncHI2A.1__BX664015, IncQ1.1__HE654726
A130	ColRI.1__DQ298019, IncFIB_S_.1__FN432031, IncFII_S_.1__CP000858
S0337107	IncQ1.1__HE654726
S0292307	IncQ1.1__HE654726
105841997	IncFIB_S_.1__FN432031, IncFII_S_.1__CP000858, IncQ1.1__HE654726
S0344705	IncI2.1_Delta_AP002527, IncQ1.1__HE654726, IncX4.1__CP002895
S0272405	IncFIB_S_.1__FN432031, IncFII_S_.1__CP000858, IncQ1.1__HE654726
S0565506	IncQ1.1__HE654726
S0657807	
S0806007	IncQ1.1__HE654726
L0064707	IncQ1.1__HE654726
S0509207	IncQ1.1__HE654726
H09256 0454	IncFIB_S_.1__FN432031, IncFII_S_.1__CP000858
H09394 0492	IncFIB_S_.1__FN432031, IncFII_S_.1__CP000858
DT120	IncQ1.1__HE654726
H09414 0613	IncFIB_S_.1__FN432031, IncFII_S_.1__CP000858
H08390 0191	
H09226 0446	IncFIA_HI1_.1_HI1_AF250878, IncFIB_S_.1__FN432031, IncFII_S_.1__CP000858, IncHI1A.1__AF250878, IncHI1B_R27_.1_R27_AF250878

DT193	IncQ1.1__HE654726
H09332 0603	ColRI.1__DQ298019, IncFIB_S_.1__FN432031, IncFII_S_.1__CP000858
H09376 0485	IncFIB_S_.1__FN432031, IncFII_S_.1__CP000858
H09366 0457	ColE10.1__X01654, ColRI.1__DQ298019, IncFIB_S_.1__FN432031, IncFII_S_.1__CP000858
H09214 0797	IncFIB_S_.1__FN432031, IncFII_S_.1__CP000858
H09164 0090	Col156.1__NC_009781, Col8282.1__DQ995353
H09270 0335	IncFIB_S_.1__FN432031, IncFII_S_.1__CP000858
DT97	IncFII_S_.1__CP000858, IncI1.1_Alpha_AP005147, IncQ1.1__HE654726
H09254 0380	IncFIB_S_.1__FN432031, IncFII_S_.1__CP000858
H09130 0134	IncFIB_S_.1__FN432031, IncFII_S_.1__CP000858
H09024 0100	ColpVC.1__JX133088, IncFIB_S_.1__FN432031, IncFII_S_.1__CP000858
H09282 0253	
H09152 0230	IncFII_pCoo_.1_pCoo_CR942285, IncFIB_S_.1__FN432031, IncFII_S_.1__CP000858, IncQ1.1__HE654726
S0344408	IncQ1.1__HE654726
L0085709	IncQ1.1__HE654726
S0354909	
L0004109	IncQ1.1__HE654726
S0387409	IncQ1.1__HE654726
S0433209	IncHI2.1__BX664015, IncHI2A.1__BX664015, IncI1.1_Alpha_AP005147, IncQ1.1__HE654726
S04696-09	
10084-1995	Col_BS512_.1__NC_010656, Col_BS512_.1__NC_010656.2, IncFIB_S_.1__FN432031, IncFII_S_.1__CP000858
100419-1995	Col_BS512_.1__NC_010656, Col_BS512_.1__NC_010656.2, IncFIB_S_.1__FN432031, IncFII_S_.1__CP000858
S00454-09	
547-2001	Col156.1__NC_009781
10902-1996	Col156.1__NC_009781, IncFIB_S_.1__FN432031, IncFII_S_.1__CP000858
7828-1995	IncFIB_S_.1__FN432031, IncFII_S_.1__CP000858
7830-1995	IncFIB_S_.1__FN432031, IncFII_S_.1__CP000858
7302-1999	

S00914-05	IncFIB_S_.1__FN432031, IncFII_S_.1__CP000858
4582-1995	IncFIB_S_.1__FN432031, IncFII_S_.1__CP000858
12005-1995	IncFIB_S_.1__FN432031, IncFII_S_.1__CP000858, IncQ1.1__HE654726
3203-1997	IncFIB_S_.1__FN432031, IncFII_S_.1__CP000858, IncQ1.1__HE654726
10984-1996	
1164-1998	
818-1998	
1713-1998	IncFII_S_.1__CP000858
S083001-02	IncFIB_S_.1__FN432031, IncFII_S_.1__CP000858
4284-1995	
4179-2001	IncFIB_S_.1__FN432031, IncFII_S_.1__CP000858
8935-1997	ColpVC.1__JX133088, IncFIB_S_.1__FN432031, IncFII_S_.1__CP000858
6940-1998	IncFIB_S_.1__FN432031, IncFII_S_.1__CP000858, IncX1.1__EU370913, IncX1.2__CP003417
S06221-07	IncI1.1_Alpha_AP005147
5544-1997	IncFII_S_.1__CP000858, IncQ1.1__HE654726
11020-1996	IncFIB_S_.1__FN432031, IncFII_S_.1__CP000858
3299-1997	IncFIB_S_.1__FN432031, IncFII_S_.1__CP000858
2610-1998	Col156.1__NC_009781
6353-1997	IncFII_S_.1__CP000858
8721-1997	IncFIA.1__AP001918, IncFIB_AP001918_.1__AP001918, IncFII_pRSB107_.1_pRSB107_AJ851089, IncQ1.1__HE654726
1402-2000	
SO3185-03	
SO9207-07	
8380-1996	IncFII_S_.1__CP000858, IncQ1.1__HE654726
SO4744-08	Col156.1__NC_009781, IncFIB_S_.1__FN432031, IncFII_S_.1__CP000858
7396-1998	IncFIB_S_.1__FN432031, IncFII_S_.1__CP000858
9115-1996	IncFII_S_.1__CP000858
388-1998	IncFIA.1__AP001918, IncFIB_AP001918_.1__AP001918, IncFII_pRSB107_.1_pRSB107_AJ851089

SO8313-02	
SO4454-08	IncFIA.1__AP001918, IncFIB_AP001918_.1__AP001918, IncFII_pRSB107_.1_pRSB107_AJ851089
SO6356-04	IncFIB_S_.1__FN432031, IncFII_S_.1__CP000858
SO1491-06	IncFIB_S_.1__FN432031, IncFII_S_.1__CP000858
SO6281-04	IncFIB_S_.1__FN432031, IncFII_S_.1__CP000858
SO4178-09	IncFIB_S_.1__FN432031, IncFII_S_.1__CP000858
8767-1998	IncFIA.1__AP001918, IncFIB_AP001918_.1__AP001918, IncFII_pRSB107_.1_pRSB107_AJ851089, IncQ1.1__HE654726
SO5416-06	
SO3433-05	IncFIB_S_.1__FN432031, IncFII_S_.1__CP000858
SO5081-04	
S00060-07	
SO9304-02	ColpVC.1__JX133088, IncFIB_S_.1__FN432031, IncFII_S_.1__CP000858
6164-1997	ColpVC.1__JX133088, IncFIB_S_.1__FN432031, IncFII_S_.1__CP000858
S09313-03	ColpVC.1__JX133088, IncFIB_S_.1__FN432031, IncFII_S_.1__CP000858
2087-1997	
S07676-03	
S05451-08	
SR11	IncFIB_S_.1__FN432031, IncFII_S_.1__CP000858
10258-1997	IncFIB_S_.1__FN432031, IncFII_S_.1__CP000858
12342-1996	IncFIB_S_.1__FN432031, IncFII_S_.1__CP000858
4300-2001	IncFIB_S_.1__FN432031, IncFII_S_.1__CP000858
SO1960-05	Col156.1__NC_009781, IncFIB_S_.1__FN432031, IncFII_S_.1__CP000858, IncQ1.1__HE654726
S04199-08	IncFIB_S_.1__FN432031, IncFII_S_.1__CP000858
S05968-02	IncFIB_S_.1__FN432031, IncFII_S_.1__CP000858, IncQ1.1__HE654726
S07292-07	IncFIB_S_.1__FN432031, IncFII_S_.1__CP000858, IncQ1.1__HE654726
S04782-03	ColE10.1__X01654, IncFIB_S_.1__FN432031, IncFII_S_.1__CP000858
L01001-10	IncQ1.1__HE654726
S01569-10	

S04797-08	IncQ1.1__HE654726
10177-1993	IncFII_S_.1__CP000858, IncQ1.1__HE654726
10246-1993	
10382-1995	IncFII_S_.1__CP000858
1013-1997	IncFII_S_.1__CP000858, IncQ1.1__HE654726
11671-1996	IncFII_S_.1__CP000858, IncQ1.1__HE654726
4061-1997	IncQ1.1__HE654726
S03512-08	IncFIB_S_.1__FN432031, IncFII_S_.1__CP000858, IncQ1.1__HE654726
S00130-09	IncQ1.1__HE654726
S02412-09	IncFIB_S_.1__FN432031, IncFII_S_.1__CP000858
L00938-09	IncI1.1_Alpha_AP005147, IncQ1.1__HE654726
L00446-08	IncFIB_S_.1__FN432031, IncFII_S_.1__CP000858
L00178-09	IncFIB_S_.1__FN432031, IncFII_S_.1__CP000858
1334-1997	IncQ1.1__HE654726
1731-1999	
6887-2000	IncFIA.1__AP001918, IncFIB_AP001918_.1__AP001918, IncFII_pRSB107_.1_pRSB107_AJ851089
2798-2001	IncFIA.1__AP001918, IncFIB_AP001918_.1__AP001918, IncFII_pRSB107_.1_pRSB107_AJ851089
3543-2002	IncFIA.1__AP001918, IncFIB_AP001918_.1__AP001918, IncFII_pRSB107_.1_pRSB107_AJ851089
L01176-08	IncQ1.1__HE654726
L01189-08	
L1101-10	Col156.1__NC_009781, IncQ1.1__HE654726
S4812-10	IncFIC_FII_.1__AP001918, IncFIB_AP001918_.1__AP001918
S4489-10	IncFIB_S_.1__FN432031, IncFII_S_.1__CP000858
S00814-10	IncQ1.1__HE654726
S03445-08	IncQ1.1__HE654726
S07300-05	IncQ1.1__HE654726
S00065-06	IncQ1.1__HE654726
S01364-10	IncQ1.1__HE654726

4824-10	IncI2.1__KP347127
4797-10	IncI2.1__KP347127
S5712-08	IncFIB_S_.1__FN432031, IncFII_S_.1__CP000858
S5828-08	IncFIB_S_.1__FN432031, IncFII_S_.1__CP000858
S03113-10	IncQ1.1__HE654726
S04698-09	IncQ1.1__HE654726
L00759-09	IncFIA_HI1_.1_HI1_AF250878, IncFIB_S_.1__FN432031, IncFII_S_.1__CP000858, IncHI1A.1__AF250878, IncHI1B_R27_.1_R27_AF250878
S3659-10	IncI2.1__KP347127
S00250-07	IncFIA.1__AP001918, IncFII_pRSB107_.1_pRSB107_AJ851089
S00176-09	Col_BS512_.1__NC_010656, Col_BS512_.1__NC_010656.2, IncQ1.1__HE654726
L00961-04	Col156.1__NC_009781, IncFII_S_.1__CP000858
5102-1999	Col_BS512_.1__NC_010656, Col_BS512_.1__NC_010656.2, IncA_C2.1__JN157804
L01730-06	IncQ1.1__HE654726
S02909-08	IncQ1.1__HE654726
S05893-09	IncQ1.1__HE654726
S05894-09	IncQ1.1__HE654726
H105100366	IncN.1__AY046276
H105260826	IncN.1__AY046276
H105280433	IncQ1.1__HE654726
H103260370	ColRI.1__DQ298019, IncQ1.1__HE654726
H103700509	IncFIB_S_.1__FN432031, IncFII_S_.1__CP000858, IncX1.1__EU370913, IncX1.2__CP003417
H103720606	Col_BS512_.1__NC_010656, Col_BS512_.1__NC_010656.2, IncA_C2.1__JN157804
H103920583	Col_BS512_.1__NC_010656, IncQ1.1__HE654726
H1041406001	IncI2.1__KP347127
H104240404	IncFIC_FII_.1__AP001918, IncFIB_AP001918_.1__AP001918, IncQ1.1__HE654726
H104680513	IncQ1.1__HE654726
H105000301	IncHI2.1__BX664015, IncHI2A.1__BX664015, IncQ1.1__HE654726
2200/2	Col156.1__NC_009781, IncQ1.1__HE654726

2448/2	Col156.1__NC_009781, ColRI.1__DQ298019, IncQ1.1__HE654726
1038/2	Col_BS512_.1__NC_010656, Col_BS512_.1__NC_010656.2, IncA_C2.1__JN157804
496/10	IncQ1.1__HE654726
1115/25	IncA_C2.1__JN157804
1686/1	ColE10.1__X01654, IncQ1.1__HE654726
1790/1	Col156.1__NC_009781, ColRI.1__DQ298019, IncI1.1_Alpha_AP005147
45/16	IncQ1.1__HE654726
2617/20	Col156.1__NC_009781, IncI2.1_Delta_AP002527, IncQ1.1__HE654726
1948/2	Col156.1__NC_009781, ColRI.1__DQ298019
1693/1	Col156.1__NC_009781
242/2	Col_BS512_.1__NC_010656, IncQ1.1__HE654726
3046/11	IncQ1.1__HE654726
1365/1	Col_BS512_.1__NC_010656, Col_BS512_.1__NC_010656.2
2841/2	IncQ1.1__HE654726
692/26	Col156.1__NC_009781, ColRI.1__DQ298019, IncI1.1_Alpha_AP005147, IncQ1.1__HE654726
2117/2	IncQ1.1__HE654726
629/2	Col156.1__NC_009781, Col_BS512_.1__NC_010656.2, IncQ1.1__HE654726
2223/2	IncQ1.1__HE654726
H07 246 0339	IncFII.1__AY458016
H07 362 0321	Col156.1__NC_009781, ColRI.1__DQ298019, IncQ1.1__HE654726
H090260055	IncFIA_HI1_.1_HI1_AF250878, IncFIB_S_.1__FN432031, IncFII_S_.1__CP000858, IncHI1A.1__AF250878, IncHI1B_R27_.1_R27_AF250878
H100120548	
H100420171	IncFIB_S_.1__FN432031, IncFII_S_.1__CP000858
H100760028	IncFIB_S_.1__FN432031, IncFII_S_.1__CP000858, IncI1.1_Alpha_AP005147
H100800267	IncQ1.1__HE654726
H101020440	IncFIA_HI1_.1_HI1_AF250878, IncFIB_S_.1__FN432031, IncFII_S_.1__CP000858, IncHI1A.1__AF250878, IncHI1B_R27_.1_R27_AF250878, IncI1.1_Alpha_AP005147
H101560198	IncFIB_S_.1__FN432031, IncFII_S_.1__CP000858
H07 016 0417	Col156.1__NC_009781, IncFIA.1__AP001918, IncFIB_AP001918_.1__AP001918, IncFII_pRSB107_.1_pRSB107_AJ851089

H102120667	IncQ1.1__HE654726
H10234093302	IncQ1.1__HE654726, IncX1.1__EU370913
H07 166 0082	IncQ1.1__HE654726
H07 182 0182	IncQ1.1__HE654726
H07 230 0280	IncQ1.1__HE654726
H07 234 0179	IncFIB_S_.1__FN432031, IncFII_S_.1__CP000858
H07 246 0338	IncQ1.1__HE654726
H07 276 0382	IncQ1.1__HE654726
H07 338 0264	IncHI2.1__BX664015, IncHI2A.1__BX664015
H07 394 0379	IncQ1.1__HE654726
VNDSal1	ColRI.1__DQ298019, IncFIB_S_.1__FN432031, IncFII_S_.1__CP000858
VNS10052	IncHI2.1__BX664015, IncHI2A.1__BX664015
VNS20005	
VNS20207	ColRI.1__DQ298019, IncFIA_HI1_.1_HI1_AF250878, IncHI1A.1__AF250878, IncHI1B_R27_.1_R27_AF250878, IncHI2.1__BX664015, IncHI2A.1__BX664015, IncQ2.1__FJ696404
VNS30099	ColRI.1__DQ298019, IncFIA_HI1_.1_HI1_AF250878, IncHI1A.1__AF250878, IncHI1B_R27_.1_R27_AF250878, IncQ1.1__HE654726, IncQ2.1__FJ696404
VNS121 DQT	ColRI.1__DQ298019, IncFIA_HI1_.1_HI1_AF250878, IncHI1A.1__AF250878, IncHI1B_R27_.1_R27_AF250878, IncQ1.1__HE654726
VNS10314	IncI1.1_Alpha_AP005147, IncQ1.1__HE654726
VNB455	IncQ1.1__HE654726
VNS10045	IncFII_S_.1__CP000858, IncHI2.1__BX664015, IncHI2A.1__BX664015, IncQ1.1__HE654726
VNB712	IncHI2.1__BX664015, IncHI2A.1__BX664015
VNB1222	ColRI.1__DQ298019, IncHI2.1__BX664015, IncHI2A.1__BX664015
VNDSal2	ColRI.1__DQ298019, IncFIB_S_.1__FN432031, IncFII_S_.1__CP000858
VNS10068	ColRI.1__DQ298019, IncFIA_HI1_.1_HI1_AF250878, IncHI1A.1__AF250878, IncHI1B_R27_.1_R27_AF250878
VNS20007	IncQ1.1__HE654726
VNS20235	IncQ1.1__HE654726, IncQ2.1__FJ696404
VNS165 VDQ	ColRI.1__DQ298019, IncFIA_HI1_.1_HI1_AF250878, IncHI1A.1__AF250878, IncHI1B_R27_.1_R27_AF250878, IncQ1.1__HE654726
VNS10413	IncHI2.1__BX664015, IncHI2A.1__BX664015

VNB148	ColRI.1__DQ298019, IncFIB_S_.1__FN432031, IncFII_S_.1__CP000858
VNB541	Col8282.1__DQ995353, ColpVC.1__JX133088, IncHI2.1__BX664015, IncHI2A.1__BX664015
VNB745	IncHI2.1__BX664015, IncHI2A.1__BX664015
VNB1264	IncHI2.1__BX664015, IncHI2A.1__BX664015
VNDSal3	ColRI.1__DQ298019, IncFIB_S_.1__FN432031, IncFII_S_.1__CP000858
VNB68	IncFIA_HI1_.1_HI1_AF250878, IncHI1A.1__AF250878, IncHI1B_R27_.1_R27_AF250878, IncQ1.1__HE654726
VNS10124	IncFIB_S_.1__FN432031, IncFII_S_.1__CP000858
VNS20018	ColRI.1__DQ298019, IncFIA_HI1_.1_HI1_AF250878, IncHI1A.1__AF250878, IncHI1B_R27_.1_R27_AF250878
VNS20277	ColRI.1__DQ298019, IncFIA_HI1_.1_HI1_AF250878, IncHI1A.1__AF250878, IncHI1B_R27_.1_R27_AF250878
VNS20150	IncFIB_S_.1__FN432031, IncFII_S_.1__CP000858, IncHI1A.1__AF250878, IncHI1B_CIT_.1_pNDM_CIT_JX182975
VNS30144	IncHI2.1__BX664015, IncHI2A.1__BX664015, IncQ1.1__HE654726
VNSC2442	IncI1.1_Alpha_AP005147, IncQ1.1__HE654726
VNB151	IncHI2.1__BX664015, IncHI2A.1__BX664015
VNB589	IncHI2.1__BX664015, IncHI2A.1__BX664015, IncQ1.1__HE654726
VNB773	ColRI.1__DQ298019, IncFII.1__AY458016, IncFIB_AP001918_.1__AP001918, IncQ1.1__HE654726, IncR.1__DQ449578, IncX1.4__JN935898
VNB1403	IncFIB_S_.1__FN432031, IncFII_S_.1__CP000858, IncX1.4__JN935898
VNDSal4	ColRI.1__DQ298019, IncFIB_S_.1__FN432031, IncFII_S_.1__CP000858
VNB1701	IncHI2.1__BX664015, IncHI2A.1__BX664015
VNS10137	ColRI.1__DQ298019, IncFII.1__AY458016, IncQ1.1__HE654726, IncR.1__DQ449578
VNS30015	ColRI.1__DQ298019, IncFIA_HI1_.1_HI1_AF250878, IncHI1A.1__AF250878, IncHI1B_R27_.1_R27_AF250878
VNS20057	ColRI.1__DQ298019
VNS20278	ColRI.1__DQ298019, IncHI1A.1__AF250878, IncHI1B_R27_.1_R27_AF250878, IncI1.1_Alpha_AP005147, IncQ1.1__HE654726
VNS30161	IncFIA_HI1_.1_HI1_AF250878, IncHI1A.1__AF250878, IncHI1B_R27_.1_R27_AF250878, IncI1.1_Alpha_AP005147, IncQ1.1__HE654726
VNSC2045	IncQ1.1__HE654726
VNB170	IncFIA_HI1_.1_HI1_AF250878, IncHI1A.1__AF250878, IncHI1B_R27_.1_R27_AF250878
VNB596	IncFIB_S_.1__FN432031, IncFII_S_.1__CP000858
VNB802	ColRI.1__DQ298019, IncHI2.1__BX664015, IncHI2A.1__BX664015
VNB1428	Col_BS512_.1__NC_010656, Col_BS512_.1__NC_010656.2, IncFIA_HI1_.1_HI1_AF250878, IncHI1A.1__AF250878, IncHI1B_R27_.1_R27_AF250878

VNDSal5	ColRI.1__DQ298019, Col_BS512_.1__NC_010656, Col_BS512_.1__NC_010656.2, IncFIB_S_.1__FN432031, IncFII_S_.1__CP000858
VNS30385	ColRI.1__DQ298019, IncHI2.1__BX664015, IncHI2A.1__BX664015, p0111.1__AP010962
VNS10146	ColRI.1__DQ298019, IncHI2.1__BX664015, IncHI2A.1__BX664015, IncQ2.1__FJ696404
VNDSal8	IncFIB_S_.1__FN432031, IncFII_S_.1__CP000858
VNS20337	ColRI.1__DQ298019, IncFIA_HI1_.1_HI1_AF250878, IncHI1A.1__AF250878, IncHI1B_R27_.1_R27_AF250878
VNS30243	IncQ1.1__HE654726
VNSC2047	ColRI.1__DQ298019, IncQ1.1__HE654726
VNB176	IncFII.1__AY458016, IncHI2.1__BX664015, IncHI2A.1__BX664015, IncQ1.1__HE654726, IncR.1__DQ449578
VNB617	IncHI2.1__BX664015, IncHI2A.1__BX664015
VNB845	IncFIC_FII_.1__AP001918, IncFIB_AP001918_.1__AP001918, IncHI2.1__BX664015, IncHI2A.1__BX664015
VNB1436	Col_BS512_.1__NC_010656, Col_BS512_.1__NC_010656.2, IncFII_S_.1__CP000858, IncHI2.1__BX664015, IncHI2A.1__BX664015, IncN.1__AY046276, IncQ1.1__HE654726
VNSC2362	ColRI.1__DQ298019, IncQ1.1__HE654726
VNDSal6	Col_BS512_.1__NC_010656.2, IncFIB_S_.1__FN432031, IncFII_S_.1__CP000858
VNS10182	IncHI2.1__BX664015, IncHI2A.1__BX664015
VNS20101	IncQ1.1__HE654726
VNS20480	ColRI.1__DQ298019
VNS30267	ColRI.1__DQ298019, Col_BS512_.1__NC_010656, Col_BS512_.1__NC_010656.2, IncFIA_HI1_.1_HI1_AF250878, IncHI1A.1__AF250878, IncHI1B_R27_.1_R27_AF250878, IncQ1.1__HE654726
VNSC2191	IncQ1.1__HE654726
VNB177	Col_BS512_.1__NC_010656, IncHI1B_R27_.1_R27_AF250878, IncHI2.1__BX664015, IncHI2A.1__BX664015
VNB652	ColRI.1__DQ298019, IncFII.1__AY458016, IncFIB_AP001918_.1__AP001918, IncQ1.1__HE654726, IncR.1__DQ449578
VNB198	Col_BS512_.1__NC_010656, Col_BS512_.1__NC_010656.2, IncFII.1__AY458016, IncFIA.1__AP001918, IncI1.1_Alpha_AP005147, IncQ1.1__HE654726
VNB922	ColRI.1__DQ298019, IncHI2.1__BX664015, IncHI2A.1__BX664015, IncQ1.1__HE654726, IncR.1__DQ449578
VNB1479	IncFIB_K_.1_Kpn3_JN233704, IncFII_S_.1__CP000858, IncQ1.1__HE654726
VNDSal7	IncFIB_S_.1__FN432031, IncFII_S_.1__CP000858
VNS20099	Col_BS512_.1__NC_010656, IncHI2A.1__BX664015, IncQ1.1__HE654726
VNS30012	ColRI.1__DQ298019, Col_BS512_.1__NC_010656.2
VNS30356	ColRI.1__DQ298019, Col_BS512_.1__NC_010656, Col_BS512_.1__NC_010656.2, IncQ1.1__HE654726

VNSC2235	Col_BS512_.1__NC_010656, Col_BS512_.1__NC_010656.2
VNB692	IncHI2.1__BX664015, IncHI2A.1__BX664015
VNB184	Col_BS512_.1__NC_010656, Col_BS512_.1__NC_010656.2, IncFIA_HI1_.1_HI1_AF250878, IncHI1A.1__AF250878, IncHI1B_R27_.1_R27_AF250878, IncQ1.1__HE654726
VNB664	ColRI.1__DQ298019, Col_BS512_.1__NC_010656, Col_BS512_.1__NC_010656.2, IncFII.1__AY458016, IncFIB_AP001918_.1__AP001918, IncQ1.1__HE654726, IncR.1__DQ449578
VNB1140	Col_BS512_.1__NC_010656, Col_BS512_.1__NC_010656.2, IncHI2.1__BX664015, IncHI2A.1__BX664015
VNB1505	IncHI2.1__BX664015, IncHI2A.1__BX664015
VNS20081	IncHI2.1__BX664015, IncHI2A.1__BX664015, IncI1.1_Alpha_AP005147
VNB1166	IncQ1.1__HE654726
S15	
S23	
S7	
ST111849	ColRI.1__DQ298019, IncFIB_S_.1__FN432031, IncFIB_AP001918_.1__AP001918, IncFII_S_.1__CP000858
ST1489	
ST1660	IncFIB_S_.1__FN432031, IncFIB_AP001918_.1__AP001918, IncFII_S_.1__CP000858, IncX1.1__EU370913, IncX1.2__CP003417
ST2143	IncFIB_S_.1__FN432031, IncFIB_AP001918_.1__AP001918, IncFII_S_.1__CP000858, IncX1.1__EU370913, IncX1.2__CP003417
ST2286	IncFIA_HI1_.1_HI1_AF250878, IncHI1A.1__AF250878, IncHI1B_R27_.1_R27_AF250878, IncI2.1__KP347127, IncQ1.1__HE654726
ST2287	IncFIB_S_.1__FN432031, IncFIB_AP001918_.1__AP001918, IncFII_S_.1__CP000858
ST2533	ColRI.1__DQ298019, IncFIA_HI1_.1_HI1_AF250878, IncHI1A.1__AF250878, IncHI1B_R27_.1_R27_AF250878, IncQ1.1__HE654726
ST2850	ColRI.1__DQ298019, IncFIB_S_.1__FN432031, IncFII_S_.1__CP000858, IncHI2.1__BX664015, IncHI2A.1__BX664015, IncI1.1_Alpha_AP005147
ST3363	
ST372	IncFIB_S_.1__FN432031, IncFIB_AP001918_.1__AP001918, IncFII_S_.1__CP000858, IncX1.1__EU370913, IncX1.2__CP003417
ST3858	IncHI2.1__BX664015, IncHI2A.1__BX664015
ST4024	IncHI2.1__BX664015, IncHI2A.1__BX664015
ST4038	
ST4329	
ST4650	
ST4848	IncFIB_S_.1__FN432031, IncFIB_AP001918_.1__AP001918, IncFII_S_.1__CP000858

ST486	
ST6988	IncFIA.1__AP001918, IncFIB_S_.1__FN432031, IncFIB_AP001918_.1__AP001918, IncFII_S_.1__CP000858, IncFII_pHN7A8_.1_pHN7A8_JN232517
ST728	
ST8493	IncFIA_HI1_.1_HI1_AF250878, IncFIB_S_.1__FN432031, IncFII_S_.1__CP000858, IncHI1A.1__AF250878, IncHI1B_R27_.1_R27_AF250878, IncQ1.1__HE654726
