# Supplementary material for: New Variant of Multidrug-Resistant Salmonella enterica Serovar Typhimurium Associated with Invasive Disease in Immunocompromised Patients in Vietnam
Source: mBio. 2018 Sep 4;9(5):e01056-18. doi: 10.1128/mBio.01056-18 (PMC6123440; doi:10.1128/mBio.01056-18)
Supplement: TABLE S7 [file mbo004184053st7.pdf]

Table S7. Genes reported as being associated with adaptation to an extraintestinal lifestyle in salmonellae by Okoro *et al.* (26) and Nuccio and Baumler (27), and the results for each from the pseudogene analysis on the 71 ST34 *S.* Typhimurium/*S.* I:4,[5],12:i:- genomes from Vietnam.

CDS	Reference genome	Product	Gene Category	Category of common disruption^	Reference	MDR (31 total)*	ancestral/ monophasic (34 total)*	transition (6 total)*
SL1873	SL1344	putative exported protein	Surface/Secreted proteins	n/a	(26)	31 intact	34 intact	6 intact
ratB	SL1344	putative outer membrane protein (RatB) (pseudogene)	Surface/Secreted proteins	n/a	(26)	28 with NS SNP	33 with NS SNP	6 with NS SNP
SL2747A	SL1344	putative transposase (pseudogene)	Phage/IS elements	n/a	(26)	20 intact; multiple hits suggesting collapsed repeats	0 intact; multiple hits suggesting collapsed repeats	6 intact; multiple hits suggesting collapsed repeats
ttdA	SL1344	tartrate dehydratase	Energy Metabolism	n/a	(26)	30 intact	33 intact	6 intact
SL2990	SL1344	possible regulatory protein	Regulators	n/a	(26)	31 intact	33 intact	6 intact
SL3733	SL1344	putative transferase	Central/Intermediary/ miscellaneous metabolism	n/a	(26)	30 intact	33 intact	6 intact
SL1475	SL1344	putative monoxygenase	Miscellaneous functions	n/a	(26)	31 intact	34 intact	6 intact
SL3051	SL1344	possible ABC-transport protein, ATP-binding component	Surface/Secreted proteins	n/a	(26)	30 with NS SNP	34 with NS SNP	6 with NS SNP
SL2653	SL1344	conserved hypothetical protein	Conserved hypothetical protein	n/a	(26)	31 intact	34 intact	6 intact
prfH	SL1344	putative peptide chain release factor	Information transfer	n/a	(26)	30 intact	34 intact	6 intact
phnT	SL1344	probable ATP-binding component of 2-aminoethylphosphonate transporter	Surface/Secreted proteins	n/a	(26)	31 intact	34 intact	6 intact
ybjZ	SL1344	conserved hypothetical ABC transporter	Surface/Secreted proteins	n/a	(26)	28 intact	34 intact	6 intact
SL1567	SL1344	putative membrane protein	Surface/Secreted proteins	n/a	(26)	31 intact	34 intact	6 intact
mdaA	SL1344	oxygen-insensitive NADPH nitroreductase (pseudogene)	Central/Intermediary/ miscellaneous metabolism	n/a	(26)	31 intact	33 intact	6 intact
cedA	SL1344	cell division activator CedA	Pathogenicity/ Adapatation/Chaperones	n/a	(26)	31 intact	34 intact	6 intact
SL3013	SL1344	conserved hypothetical	Conserved hypothetical	n/a	(26)	31 intact	34 intact	6 intact

		protein	protein					
pepT	SL1344	Peptidase T	Degradation of large molecules	n/a	(26)	30 intact	34 intact	6 intact
hilC	SL1344	possible AraC-family transcriptional regulator (SPI1-associated)	Pathogenicity/ Adapatation/Chaperones	n/a	(26)	31 intact	33 intact	6 intact
SL2208	SL1344	putative lipopolysaccharide modification acyltransferase	Surface/Secreted proteins	n/a	(26)	28 intact	33 intact	6 intact
SL4152	SL1344	hypothetical protein	Pathogenicity/ Adapatation/Chaperones	n/a	(26)	31 intact	34 intact	6 intact
SL1688	SL1344	putative secreted protein	Surface/Secreted proteins	n/a	(26)	30 intact	34 intact	6 intact
amyA	SL1344	cytoplasmic alpha-amylase	Degradation of large molecules	n/a	(26)	31 with NS SNP	34 with NS SNP	6 with NS SNP
sspH2	SL1344	secreted effector protein	Pathogenicity/ Adapatation/Chaperones	n/a	(26)	multiple hits suggesting collapsed repeats	multiple hits suggesting collapsed repeats	multiple hits suggesting collapsed repeats
SL4345	SL1344	putative sugar transporter	Surface/Secreted proteins	n/a	(26)	31 intact	34 intact	6 intact
bcfB	SL1344	fimbrial chaperone	Surface/Secreted proteins	n/a	(26)	31 with NS SNP	34 with NS SNP	6 with NS SNP
SL1873	SL1344	putative exported protein	Surface/Secreted proteins	n/a	(26)	31 intact	34 intact	6 intact
adiY	SL1344	putative AraC family regulatory protein	Regulators	n/a	(26)	31 with NS SNP	34 with NS SNP	6 with NS SNP
srfB	SL1344	putative virulence effector protein	Pathogenicity/ Adapatation/Chaperones	n/a	(26)	31 intact	34 intact	6 intact
fliY	SL1344	cystine-binding periplasmic protein *(FliY)*	Surface/Secreted proteins	n/a	(26)	31 intact	34 intact	6 intact
SL3360	SL1344	putative exported protein	Surface/Secreted proteins	n/a	(26)	31 intact	34 intact	6 intact
rpoS	SL1344	RNA polymerase sigma subunit RpoS (sigma-38)	Regulators	n/a	(26)	31 intact	34 intact	6 intact
SL1567	SL1344	putative membrane protein	Surface/Secreted proteins	n/a	(26)	31 intact	34 intact	6 intact
SL2659	SL1344	conserved hypothetical protein	Conserved hypothetical protein	n/a	(26)	31 intact	34 intact	6 intact
yhjU	SL1344	putative membrane protein	Surface/Secreted proteins	n/a	(26)	31 intact	33 intact	5 intact
SL4223	SL1344	putative membrane-bound beta-	Surface/Secreted proteins	n/a	(26)	31 intact	34 intact	6 intact

		hydroxylase						
SL2214	SL1344	putative phage protein	Conserved hypothetical protein	n/a	(26)	31 intact	31 intact	6 intact
yadE	SL1344	conserved hypothetical protein	Surface/Secreted proteins	n/a	(26)	31 intact	33 intact	6 intact
SL4256	SL1344	putative exported protein	Surface/Secreted proteins	n/a	(26)	31 intact	34 intact	6 intact
SL2243	SL1344	putative transmembrane transpot protein	Central/Intermediary/ miscellaneous metabolism	n/a	(26)	31 intact	34 intact	6 intact
hepA	SL1344	probable ATP-dependent helicase HepA	Regulators	n/a	(26)	30 intact	34 intact	6 intact
manZ	SL1344	putative mannose specific permease	Surface/Secreted proteins	n/a	(26)	30 intact	34 intact	6 intact
SL1188	SL1344	hypothetical protein	Surface/Secreted proteins	n/a	(26)	30 intact	34 intact	6 intact
SL1149	SL1344	putative secreted protein	Surface/Secreted proteins	n/a	(26)	31 intact	34 intact	6 intact
rfc	SL1344	O-antigen polymerase	Conserved hypothetical protein	n/a	(26)	30 with NS SNP	34 with NS SNP	6 with NS SNP
SL0878	SL1344	conserved hypothetical protein	Pathogenicity/ Adapatation/Chaperones	n/a	(26)	31 intact	34 intact	6 intact
ratA	SL1344	putative exported protein (RatA)	Information transfer	n/a	(26)	28 intact	34 intact	6 intact
nlpD	SL1344	lipoprotein NlpD precursor	Information transfer	n/a	(26)	31 intact	34 intact	6 intact
ada	SL1344	ADA regulatory protein	Central/Intermediary/ miscellaneous metabolism	n/a	(26)	31 intact	33 intact	6 intact
nrdF	SL1344	ribonucleoside-diphosphate reductase 2 beta chain	Central/Intermediary/ miscellaneous metabolism	n/a	(26)	31 intact	34 intact	6 intact
SL3103	SL1344	possible aldehyde dehydrogenase	Phage/IS elements	n/a	(26)	31 intact	34 intact	6 intact
SeAg_B0155	Agona SL483	Aldo-keto reductase YakC (NADP+) [EC=1.1.1.-]		GI	(27)	0 assembled	0 assembled	0 assembled
SeAg_B0156	Agona SL483	Transcriptional regulator, LysR family		GI	(27)	0 reported	0 reported	0 reported
SEN1331	Enteritidis P125109	Uncharacterized protein		EI	(27)	multiple NS hits	multiple NS hits	multiple NS hits
STM0658	LT2	Putative molecular chaperone, DnaJ family		EI	(27)	29 intact	34 intact	6 intact
STM0810	LT2	Putative inner membrane protein		EI	(27)	31 with frameshift	34 with frameshift	6 with frameshift

STM1304	LT2	Arginine N-succinyltransferase [EC=2.3.1.109]	EI	(27)	31 intact	34 intact	6 intact
STM1543	LT2	Putative transport protein	EI	(27)	31 intact	34 intact	6 intact
STM1544	LT2	PhoPQ-regulated protein	EI	(27)	31 intact	34 intact	6 intact
STM1545	LT2	Putative multidrug efflux protein	EI	(27)	31 intact	33 intact	6 intact
STM1546	LT2	Putative monooxygenase	EI	(27)	31 intact	34 intact	6 intact
STM1547	LT2	Putative marR-family transcriptional regulator	EI	(27)	31 intact	34 intact	6 intact
STM1548.s	LT2	Putative S-adenosylmethionine:tRNA-ribosyltransferase-isomerase	EI	(27)	31 intact	34 intact	6 intact
STM1551.1n	LT2	-	EI	(27)	31 intact	33 intact	6 intact
STM1555	LT2	Putative transcriptional regulator	EI	(27)	31 intact	34 intact	6 intact
STM1556	LT2	Putative Na+/H+ antiporter	EI	(27)	31 intact	34 intact	6 intact
STM1630	LT2	Putative inner membrane protein	EI	(27)	31 intact	34 intact	6 intact
STM2066	LT2	E3 ubiquitin-protein ligase SopA [EC=6.3.2.-]	EI	(27)	31 intact	34 intact	6 intact
STM2189	LT2	Galactose/methyl galactoside import ATP-binding protein MglA [EC=3.6.3.17]	EI	(27)	31 intact	33 intact	6 intact
STM2961	LT2	Putative d-glucarate dehydratase [EC=4.2.1.40]	EI	(27)	31 intact	34 intact	6 intact
STM3858	LT2	Putative phosphotransferase system fructose-specific component IIB [EC=2.7.1.69]	EI	(27)	31 with 2 NS SNPs	34 with 2 NS SNPs	6 with 2 NS SNPs
STM4519	LT2	Putative NAD-dependent aldehyde dehydrogenase [EC=1.2.1.16]	EI	(27)	31 intact	34 intact	6 intact

^ pathovar in which CDS is commonly disrupted according to ref. Nuccio and Baumler (27); extraintestinal (EI) or gastrointestinal (GI)

*in cases where the total in the cell does not match the total number of isolates, the remaining isolates contained varying different ARIBA flags, but are not reported as are rare and do not represent a trend

*NS = nonsynonymous
