# Supplementary material for: New Variant of Multidrug-Resistant Salmonella enterica Serovar Typhimurium Associated with Invasive Disease in Immunocompromised Patients in Vietnam
Source: mBio. 2018 Sep 4;9(5):e01056-18. doi: 10.1128/mBio.01056-18 (PMC6123440; doi:10.1128/mBio.01056-18)
Supplement: TEXT S1 [file mbo004184053s1.pdf]

Supplemental Text

Supplemental methods

Vietnamese collection of Salmonella *Typhimurium*

The 41 isolates from human bloodstream infections are described in Lan *et al.* (1). Briefly, these isolates were collected between 2008 and 2013 from HIV infected patients admitted to the Hospital for Tropical Diseases (HTD) in Ho Chi Minh City (HCMC), Vietnam whom presented with febrile illness. The 36 isolates from human diarrheal patients were collected between 2009 and 2011 from children attending hospitals in HCMC with acute diarrhea (2). The eight isolates from asymptomatic humans were collected in 2010 (2), with one isolate each from 1997 and 1998 originating from a historical diarrheal disease study (3). The origins of the 113 animal isolates derive from two studies which are described in (4-6). Briefly, one study visited >200 multi-animal farms in one province over a period of a year (6), and the other visited >300 multi-animal farms in one province over a five-month period (4, 5).

Assembly and annotation of Vietnamese S. *Typhimurium isolates*

The pipeline described in Page *et al.* (7) was used to assemble and annotate the draft genomes of the *Salmonella* isolates from Vietnam. Sequence reads were used to create multiple assemblies using VelvetOptimiser v2.2.5 (8) and Velvet v1.2 (9). Assembly improvement was undertaken using the assembly with the best N50, contigs were scaffolded using SSPACE (10), and sequence gaps filled using GapFiller (11). Automated annotation of the assembled genomes was performed using PROKKA v1.11 (12), and the *Salmonella* database from RefSeq (13). Sequence types (STs)

were again determined using MLSTcheck (14), which was used to compare the assembled genomes against the MLST database for *Salmonella* (15).

Assessment of potential confounding of phylogenetic signal by date of isolation

Given that the animal isolates were sampled over a shorter temporal range (2011-2013) than the human isolates (one isolate each from 1997 and 1998, the rest from 2008-2013), we assessed whether or not the observed clustering of isolates by host population was confounded by year of isolation. To do this, we extracted the phylogeny of the human isolates only from the phylogenetic tree represented in Figure 1 using drop.tip in the ape package (16). The phylosig function of the R phytools package (17) was used to calculate Pagel's lambda, which estimates the phylogenetic signal of a continuous trait (here, year of isolation). When lambda=1, the structure of the phylogeny alone can explain changes in the trait (i.e., isolates cluster by year of isolation), and when lambda=0, the phylogeny has to lose all its structure to be able to explain trait evolution under a Brownian motion model (i.e., isolates do not cluster by year of isolation).

Phase switching

The genotypic identification of biphasic or monophasic *S.* Typhimurium of the Vietnam ST34 isolates were confirmed using phase-changing assays as previously described in Baker *et al.* (18). Briefly, Craigie tubes were prepared using sterile glass vessels containing semi-solid nutrient agar and a small inner tube open at both ends with the upper end projecting above the agar. Bacterial swim media was prepared using Luria-Bertani (LB) broth containing 0.3% agar and then either not supplemented or supplemented with rabbit anti-H:i (1:100 dilution) or anti-H:1 (1:100 dilution) antiserum (19). Several colonies of *S.* Typhimurium were inoculated into the tubes, which were

incubated without agitation at 37°C until motile bacteria were observed on the exterior of the tubing and/or the surface of the media. Bacterial suspensions taken from outside the inner tube were agglutinated with anti-H:i and anti-H:1 antiserum to confirm the expression of Phase 2 flagella.

For phase switching visualization, *S.* Typhimurium expressing flagella were prepared for immunofluorescence imaging using a modified version of a previously described method (20). Bacterial suspensions taken from the surface of the Craigie tube were diluted 1/100 in PBS and 10µl placed on a glass slide. Bacteria were heat fixed (50°C for 15 minutes) on slides, washed twice with PBS and fixed in 4% paraformaldehyde in PBS for 30 minutes at ambient temperature. After washing, bacterial cells were incubated in either rabbit H:i or rabbit H:1 antiserum (diluted 1/200 in PBS containing 3% BSA and 0.1% Triton X-100) for 30 minutes at ambient temperature in a humid atmosphere. Slides were washed and secondary antibody (Alexa-Fluor 488 and 594 labeled goat anti-rabbit IgG antibodies (Invitrogen, USA), diluted 1/2000 in PBS containing 3% BSA and 0.1% Triton X-100) was applied for 30 minutes at ambient temperature in the dark in a humid atmosphere. Following incubation, slides were washed, stained with 300nM 4',6-Diamidino-2-Phenylindole (DAPI) (Sigma), and visualized using a fluorescence microscope (Ni-E, Nikon).

Long-read sequencing

Sequence reads for the VNB151 isolate additionally sequenced using the Pacific Biosciences platform were assembled using HGAP v3 (21) of the SMRT analysis software v2.3.0 (22); the fold coverage to target when selecting the minimum fragment length for assembly was set to 30 and

the approximate genome size was set to 3Mbp. The assembly was circularized using Circlator v1.1.3 (23) and the pre-assembled (corrected) reads. The circularized assembly was polished using the PacBio RS_Resequencing protocol, Quiver v1 of the SMRT analysis software v2.3.0 (22), followed by Pilon (24) using the Illumina short reads. Automated annotation of the assembled genome was performed using PROKKA v1.11 (12) and the *Salmonella* database from RefSeq (13).

Identification of pseudogenes in ST34 isolates

Putative pseudogenes were identified using ARIBA (25) with the coding sequences of the SL1344 chromosome (accession FQ312003) as the reference database. From each of the detailed reports produced, all genes which were reported as having a deletion, frameshift, indel, insertion, truncation, or multiple effect compared to the reference gene were extracted ('ref_ctg_effect' = DEL, FSHIFT, INDELS, INS, MULTIPLE or TRUNC). Genes which had no reported change or had non-synonymous SNPs were excluded, as the functional effects of non-synonymous SNPs could not be verified. For each of these effect categories, the numbers of isolates in each of the three sub-groups (ancestral/monophasic, transitional, MDR) were tabulated. In addition, pseudogenes which have been found to be associated with adaptation of *S.* Typhimurium ST313 to the extraintestinal lifestyle by Okoro *et al.* (26) (Table S3 of that publication) and genes found commonly and differentially disrupted or deleted in *Salmonella* associated with either extraintestinal or gastrointestinal lifestyles by Nuccio and Baumler (27) (Table S5 of that publication) were examined in each of the Vietnamese ST34 isolates using the results from the ARIBA analysis. For genes listed in Okoro *et al.*'s table that are pseudogenes in SL1344 and

therefore excluded from the ARIBA analysis, the equivalent intact genes from *S.* Typhimurium LT2 (accession AE006468) were used; for the genes listed in Nuccio and Baumler's table, the indicated genes were taken from the relevant reference genome (accessions LT2 AE006468, *S.* Agona SL483 CP001138, *S.* Enteritidis P125109 AM933172) and used to make a database with which to run ARIBA.

Supplemental results

Using the described approaches, we additionally report the first incidence of *S.* Typhimurium ST313 in Asia (1), the clone that has emerged as a leading cause of iNTS infections in Africa (28). In contrast to the African setting, this ST313 (isolate Hue_98) came from a human patient in Vietnam with diarrhea, and was susceptible to all tested antimicrobials.

We additionally identified a chicken isolate (71_G_169) with the transferable colistin resistance gene *mcr-1*; the presence of this gene in Vietnam has been previously reported in *Escherichia coli* from pigs (29). This isolate also carried multiple additional AMR genes, namely *qnrS1*, *blaTEM-1*, *aadA1*, *aadA2*, *cmlA1*, *dfrA12*, *floR*, *tetA*, *tetM*, *sul2*, and *sul3*.

References

1. Phu Huong Lan N, Le Thi Phuong T, Nguyen Huu H, Thuy L, Mather AE, Park SE, Marks F, Thwaites GE, Van Vinh Chau N, Thompson CN, Baker S. 2016. Invasive non-typhoidal *Salmonella* infections in Asia: clinical observations, disease outcome and dominant serovars from an infectious disease hospital in Vietnam. PLoS Negl Trop Dis 10:e0004857.
2. Thompson CN, Phan MV, Hoang NV, Minh PV, Vinh NT, Thuy CT, Nga TT, Rabaa MA, Duy PT, Dung TT, Phat VV, Nga TV, Tu le TP, Tuyen HT, Yoshihara K, Jenkins C, Duong VT, Phuc HL, Tuyet PT, Ngoc NM, Vinh H, Chinh NT, Thuong TC, Tuan HM, Hien TT, Campbell JI, Chau NV, Thwaites G, Baker S. 2015. A prospective multi-center

observational study of children hospitalized with diarrhea in Ho Chi Minh City, Vietnam. Am J Trop Med Hyg 92:1045-52.

3. Vinh H, Nhu NT, Nga TV, Duy PT, Campbell JI, Hoang NV, Boni MF, My PV, Parry C, Nga TT, Van Minh P, Thuy CT, Diep TS, Phuong le T, Chinh MT, Loan HT, Tham NT, Lanh MN, Mong BL, Anh VT, Bay PV, Chau NV, Farrar J, Baker S. 2009. A changing picture of shigellosis in southern Vietnam: shifting species dominance, antimicrobial susceptibility and clinical presentation. BMC Infect Dis 9:204.
4. Tu LT, Hoang NV, Cuong NV, Campbell J, Bryant JE, Hoa NT, Kiet BT, Thompson C, Duy DT, Phat VV, Hien VB, Thwaites G, Baker S, Carrique-Mas JJ. 2015. High levels of contamination and antimicrobial-resistant non-typhoidal *Salmonella* serovars on pig and poultry farms in the Mekong Delta of Vietnam. Epidemiol Infect 143:3074-3086.
5. Rabaa MA, Tue NT, Phuc TM, Carrique-Mas J, Saylors K, Cotten M, Bryant JE, Nghia HD, Cuong NV, Pham HA, Berto A, Phat VV, Dung TT, Bao LH, Hoa NT, Wertheim H, Nadjm B, Monagin C, van Doorn HR, Rahman M, Tra MP, Campbell JI, Boni MF, Tam PT, van der Hoek L, Simmonds P, Rambaut A, Toan TK, Van Vinh Chau N, Hien TT, Wolfe N, Farrar JJ, Thwaites G, Kellam P, Woolhouse ME, Baker S. 2015. The Vietnam Initiative on Zoonotic Infections (VIZIONS): A strategic approach to studying emerging zoonotic infectious diseases. Ecohealth 12:726-735.
6. Trung NV, Carrique-Mas JJ, Nghia NH, Tu LT, Mai HH, Tuyen HT, Campbell J, Nhung NT, Nhung HN, Minh PV, Chieu TT, Hieu TQ, Mai NT, Baker S, Wagenaar JA, Hoa NT, Schultsz C. 2017. Non-typhoidal *Salmonella* colonization in chickens and humans in the Mekong Delta of Vietnam. Zoonoses Public Health 64:94-99.
7. Page AJ, De Silva N, Hunt M, Quail MA, Parkhill J, Harris SR, Otto TD, Keane JA. 2016. Robust high throughput prokaryote *de novo* assembly and improvement pipeline for Illumina data. Microb Genom 2:doi: 10.1099/mgen.0.000083.
8. Gladman S, Seemann T. 2008. Velvet Optimiser: For automatically optimising the primary parameter options for the Velvet *de novo* sequence assembler, http://bioinformatics.net.au/software.velvetoptimiser.shtml.
9. Zerbino DR, Birney E. 2008. Velvet: algorithms for *de novo* short read assembly using de Bruijn graphs. Genome Res 18:821-9.
10. Boetzer M, Henkel CV, Jansen HJ, Butler D, Pirovano W. 2011. Scaffolding pre-assembled contigs using SSPACE. Bioinformatics 27:578-9.
11. Boetzer M, Pirovano W. 2012. Toward almost closed genomes with GapFiller. Genome Biology 13.
12. Seemann T. 2014. Prokka: rapid prokaryotic genome annotation. Bioinformatics 30:2068-9.
13. Pruitt KD, Tatusova T, Brown GR, Maglott DR. 2012. NCBI Reference Sequences (RefSeq): current status, new features and genome annotation policy. Nucleic Acids Res 40:D130-5.
14. Page AJ, Taylor B, Keane JA. 2016. Multilocus sequence typing by blast from *de novo* assemblies against PubMLST. JOSS:doi: http://dx.doi.org/10.21105/joss.00118.
15. Jolley KA, Maiden MC. 2010. BIGSdb: Scalable analysis of bacterial genome variation at the population level. BMC Bioinformatics 11:595.
16. Paradis E, Claude J, Strimmer K. 2004. APE: Analyses of phylogenetics and evolution in R language. Bioinformatics 20:289-290.

17. Revell LJ. 2012. phytools: an R package for phylogenetic comparative biology (and other things). Methods in Ecology and Evolution 3:217-223.
18. Baker S, Holt K, Whitehead S, Goodhead I, Perkins T, Stocker B, Hardy J, Dougan G. 2007. A linear plasmid truncation induces unidirectional flagellar phase change in H:z66 positive *Salmonella* Typhi. Mol Microbiol 66:1207-18.
19. Craigie J. 1931. Studies on the serological reactions of the flagella of *B. Typhosus*. J Immunol 21:417-511.
20. Wain J, House D, Zafar A, Baker S, Nair S, Kidgell C, Bhutta Z, Dougan G, Hasan R. 2005. Vi antigen expression in *Salmonella enterica* serovar Typhi clinical isolates from Pakistan. J Clin Microbiol 43:1158-65.
21. Chin CS, Alexander DH, Marks P, Klammer AA, Drake J, Heiner C, Clum A, Copeland A, Huddleston J, Eichler EE, Turner SW, Korlach J. 2013. Nonhybrid, finished microbial genome assemblies from long-read SMRT sequencing data. Nat Methods 10:563-9.
22. PacBio. SMRT-Analysis: A software suite for analyzing single molecule, real-time DNA sequencing data, https://github.com/PacificBiosciences/SMRT-Analysis.
23. Hunt M, Silva ND, Otto TD, Parkhill J, Keane JA, Harris SR. 2015. Circlator: automated circularization of genome assemblies using long sequencing reads. Genome Biol 16:294.
24. Walker BJ, Abeel T, Shea T, Priest M, Abouelliel A, Sakthikumar S, Cuomo CA, Zeng Q, Wortman J, Young SK, Earl AM. 2014. Pilon: an integrated tool for comprehensive microbial variant detection and genome assembly improvement. PLoS One 9:e112963.
25. Hunt M, Mather AE, Sanchez-Buso L, Page AJ, Parkhill J, Keane JA, Harris SR. 2017. ARIBA: rapid antimicrobial resistance genotyping directly from sequencing reads. Microb Genom:doi: 10.1099/mgen.0.000131.
26. Okoro CK, Barquist L, Connor TR, Harris SR, Clare S, Stevens MP, Arends MJ, Hale C, Kane L, Pickard DJ, Hill J, Harcourt K, Parkhill J, Dougan G, Kingsley RA. 2015. Signatures of adaptation in human invasive *Salmonella* Typhimurium ST313 populations from sub-saharan Africa. PLoS Negl Trop Dis 9:e0003611.
27. Nuccio SP, Baumler AJ. 2014. Comparative analysis of *Salmonella* genomes identifies a metabolic network for escalating growth in the inflamed gut. MBio 5:e00929-14.
28. Okoro CK, Kingsley RA, Connor TR, Harris SR, Parry CM, Al-Mashhadani MN, Kariuki S, Msefula CL, Gordon MA, de Pinna E, Wain J, Heyderman RS, Obaro S, Alonso PL, Mandomando I, MacLennan CA, Tapia MD, Levine MM, Tennant SM, Parkhill J, Dougan G. 2012. Intracontinental spread of human invasive *Salmonella* Typhimurium pathovariants in sub-Saharan Africa. Nat Genet 44:1215-21.
29. Malhotra-Kumar S, Xavier BB, Das AJ, Lammens C, Hoang HT, Pham NT, Goossens H. 2016. Colistin-resistant *Escherichia coli* harbouring *mcr-1* isolated from food animals in Hanoi, Vietnam. Lancet Infect Dis 16:286-7.
